# Teleophthalmology-enabled and artificial intelligence-ready referral pathway for community optometry referrals of retinal disease (HERMES): a Cluster Randomised Superiority Trial with a linked Diagnostic Accuracy Study—HERMES study report 1—study protocol

**DOI:** 10.1136/bmjopen-2021-055845

**Published:** 2022-02-01

**Authors:** Ji Eun Diana Han, Xiaoxuan Liu, Catey Bunce, Abdel Douiri, Luke Vale, Ann Blandford, John Lawrenson, Rima Hussain, Gabriela Grimaldi, Annastazia E Learoyd, Ashleigh Kernohan, Christiana Dinah, Evangelos Minos, Dawn Sim, Tariq Aslam, Praveen J Patel, Alastair K Denniston, Pearse A Keane, Konstantinos Balaskas

**Affiliations:** 1 University of Birmingham Institute of Inflammation and Ageing, Birmingham, UK; 2 University Hospitals Birmingham NHS Foundation Trust, Birmingham, UK; 3 RM CTU, Royal Marsden Hospital NHS Trust, London, UK; 4 School of Population Health and Environmental Sciences, King’s College London, London, UK; 5 Health Economics Group, Institute of Health and Society, Newcastle University, Newcastle upon Tyne, Tyne and Wear, UK; 6 UCLIC, University College London, London, UK; 7 Dvision of Optometry and Visual Science, City University of London, London, UK; 8 NIHR Biomedical Research Centre, Moorfields Eye Hospital NHS Foundation Trust, London, London, UK; 9 Moorfields Ophthalmic Reading Centre and Artificial Intelligence Lab, Moorfields Eye Hospital NHS Foundation Trust, London, UK; 10 Medical Retina Service, Moorfields Eye Hospital NHS Foundation Trust, London, UK; 11 Population Health Sciences Institute, Newcastle University, Newcastle upon Tyne, Tyne and Wear, UK; 12 Ophthalmology, London North West Healthcare NHS Trust, Harrow, UK; 13 North West Anglia NHS Foundation Trust, Peterborough, Cambridgeshire, UK; 14 Institute of Ophthalmology, University College London, London, UK; 15 University of Manchester, Manchester, UK

**Keywords:** ophthalmology, medical retina, health services administration & management, health economics, telemedicine

## Abstract

**Introduction:**

Recent years have witnessed an upsurge of demand in eye care services in the UK. With a large proportion of patients referred to Hospital Eye Services (HES) for diagnostics and disease management, the referral process results in unnecessary referrals from erroneous diagnoses and delays in access to appropriate treatment. A potential solution is a teleophthalmology digital referral pathway linking community optometry and HES.

**Methods and analysis:**

The HERMES study (Teleophthalmology-enabled and artificial intelligence-ready referral pathway for community optometry referrals of retinal disease: a cluster randomised superiority trial with a linked diagnostic accuracy study) is a cluster randomised clinical trial for evaluating the effectiveness of a teleophthalmology referral pathway between community optometry and HES for retinal diseases. Nested within HERMES is a diagnostic accuracy study, which assesses the accuracy of an artificial intelligence (AI) decision support system (DSS) for automated diagnosis and referral recommendation. A postimplementation, observational substudy, a within-trial economic evaluation and discrete choice experiment will assess the feasibility of implementation of both digital technologies within a real-life setting. Patients with a suspicion of retinal disease, undergoing eye examination and optical coherence tomography (OCT) scans, will be recruited across 24 optometry practices in the UK. Optometry practices will be randomised to standard care or teleophthalmology. The primary outcome is the proportion of false-positive referrals (unnecessary HES visits) in the current referral pathway compared with the teleophthalmology referral pathway. OCT scans will be interpreted by the AI DSS, which provides a diagnosis and referral decision and the primary outcome for the AI diagnostic study is diagnostic accuracy of the referral decision made by the Moorfields-DeepMind AI system. Secondary outcomes relate to inappropriate referral rate, cost-effectiveness analyses and human–computer interaction (HCI) analyses.

**Ethics and dissemination:**

Ethical approval was obtained from the London—Bromley Research Ethics Committee (REC 20/LO/1299). Findings will be reported through academic journals in ophthalmology, health services research and HCI.

**Trial registration number:**

ISRCTN18106677 (protocol V.1.1).

**Strengths and limitations of this study**

- The HERMES study (Teleophthalmology-enabled and artificial intelligence-ready referral pathway for community optometry referrals of retinal disease: a cluster randomised superiority trial with a linked diagnostic accuracy study) is a prospective, multicentre implementation science study assessing clinical utility, cost-effectiveness and human–computer interaction of Tele-Medicine and Artificial Intelligence Decision Support Systems in eye care referral pathways.
- The HERMES study incorporates three intertwined implementation science designs for digital eye care: an interventional, cluster randomised controlled trial of telemedicine, an observational postimplementation study of telemedicine and a prospective diagnostic accuracy study of artificial intelligence decision support systems.
- The study includes an embedded comprehensive economic evaluation, coupled with a human–computer Iiteraction analysis, generating evidence of enablers and barriers to real-life adoption of digital pathways.
- One limitation of the study is that the assessed care pathways pertain to the UK healthcare setting and may not be directly generalisable to other health systems, although reflecting a global trend towards digital transformation of healthcare.

## Background

Ophthalmology outpatient attendances account for 10% of all outpatient activities in the UK, more than any other individual medical specialty.^1 2^ Modern ophthalmic practice in the UK is faced by the challenges of an ageing population, increasing prevalence of degenerative disease and emergent treatments that are revolutionary but dependent on timely diagnosis. This represents a huge strain on diagnostic services and adversely impacts on timely access to care. Concurrently, there have been exponential increases in computing power and artificial intelligence (AI), expansions in the strength and ubiquity of communications technologies and developments in imaging capabilities, including in the community optometry setting.^3^

In the UK, primary care for ophthalmology is delivered by community-based optometry practices (high street opticians). A large proportion of patients diagnosed with a suspicion of retinal disease, including common conditions such as neovascular (‘wet’) age-related macular degeneration (AMD), are referred to Hospital Eye Services (HES) for diagnostics and disease management.^4 5^ The referral process results in unnecessary referrals (which can cause inconvenience and distress for patients), erroneous diagnoses, misclassification in terms of urgency, duplication of imaging tests and delays in access to treatment.

An increase in 30% in eye clinic attendances has been observed within the last 5 years throughout the UK.^6^ Further increases are likely because of the increasing availability of imaging technology, and especially optical coherence tomography (OCT), which is becoming ubiquitous in community optometry practices.^7^ OCT is a non-invasive imaging modality that uses light to generate micrometre-resolution three-dimensional images of the retina and provides the best way to diagnose a number of common retinal pathologies including wet AMD.

This study focuses on two potentially complementary digital technologies that have the potential to revolutionise the interface between community optometrists and hospital-based eye clinics: the teleophthalmology platform and the Moorfields-DeepMind AI DSS. The technologies will be assessed through two complementary and linked quantitative studies:

1. Cluster Superiority Randomised Trial (RCT) of teleophthalmology.
2. Prospective diagnostic accuracy study of AI (machine learning) DSS (the Moorfields-DeepMind algorithm).

### Cluster superiority randomised trial (RCT) of teleophthalmology pathway

Telemedicine in ophthalmology can help face this challenge of provision of optimal and expert care to people attending for routine eye tests in community optometry practices, through a digital referral pathway relying on teleophthalmology links between community optometry and HES. This could optimise the referral process by allowing remote review of imaging and clinical data captured at the community level, by human experts based in HES. We will perform a cluster randomised superiority trial to assess the impact on service delivery metrics (such as proportion of unnecessary referrals and time from referral to treatment for urgent maculopathies) of a digital link between community optometry practices and HES using a teleophthalmology platform. We will use a device-agnostic, teleophthalmology platform to enable a digital referral pathway of patients with a suspicion of retinal disease. The pilot data produced by our research team have demonstrated the potential of teleophthalmology to drastically improve the efficiency of the referral pathway between community optometry and HES while reducing unnecessary referrals to HES^8^ and; hence, we propose a randomised trial powered to demonstrate superiority of the digital referral pathway against standard care.

### Diagnostic accuracy study of AI diagnosis support system

AI DSSs have recently been developed and shown to have good diagnostic accuracy against human experts in interpreting ocular imaging tests, such as OCT scan.^9^ The collaboration between Moorfields Eye Hospital and Google DeepMind produced the arguably most advanced deep learning DSS in ophthalmology, capable of interpreting OCT scans, providing diagnosis for retinal disease and suggesting urgency of referral. In silico analysis using retrospectively collected data has validated the tool against human experts for the diagnosis of retinal disease and referral recommendations, and it has been shown to be non-inferior.^9^ The algorithm is uniquely and independently used in Moorfields Eye Hospital for research purposes. Thus, while such work has demonstrated promising results, a prospective study is required to demonstrate its value in practice.

We will perform a prospective study to assess diagnostic (referral) accuracy of the Moorfields-DeepMind AI DSS when applied on the OCT scans collected in the context of the teleophthalmology RCT. This will allow maximum utilisation of collected data from the trial and will provide estimates and CIs of diagnostic (referral) accuracy. All cases included in the RCT will be reviewed by the Moorfields-DeepMind AI DSS within 48 hours of obtaining the OCT scans, and a referral decision (refer routinely, refer urgently, don’t refer) will be made by the algorithm for each case and recorded. The referral decisions made by the AI DSS will not be implemented in practice, yet data of the time required to obtain these decisions and any technical issues encountered with its use will be captured. These data will be incorporated into the implementation science models, including human–computer interaction (HCI) analysis, value-based economic evaluation and discrete choice experiment (DCE), to identify the potential opportunities and gaps in advancing the adoption of the Moorfields-DeepMind AI DSS.

### Impact of the COVID-19 pandemic: postimplementation observational pragmatic subtudy

National Health Service (NHS) services underwent rapid and significant adjustments across the board in response to extreme challenges presented by the COVID-19 pandemic. Changes to healthcare services driven by necessity are not always underpinned by a robust evidence base for efficiency and safety. In ophthalmology, as a response to the need for social distancing and minimising unnecessary hospital visits, teleophthalmology pathways were commissioned recently in some areas of England using digital link to facilitate referrals between community optometry and HESs. Greater Manchester was an early adopter of this approach and a majority of the optometry practices in that area are now referring to NHS eye units via a teleophthalmology link. This local change in standard care provides a unique opportunity to examine whether teleophthalmology works under usual conditions within the NHS. This substudy will allow us to record and measure variation in quality of healthcare within a local region to inform our inferences from the RCT on how the teleophthalmology pathway will perform within a real-life setting. We will, thus, perform a pragmatic, observational, postimplementation study involving community optometry practices in the Greater Manchester area. This will also serve as a safety analysis allowing us to identify potential safety signals of the teleophthalmology pathways and adding granularity to the economic and qualitative evaluations of the RCT.

#### Trial aims and objectives

There are two complementary aims (aims 1 and 2) pertaining to the linked quantitative studies assessing the two digital technologies (‘teleophthalmology’ and the ‘Moorfields-DeepMind’ AI). The qualitative research element (aim 3) using HCI methodology will run across both studies to provide evidence on implementation (table 1).

**Table 1:** Aims and objectives of the HERMES study

Aims	Objectives
1. To assess the effectiveness and efficiency of a digital referral pathway between community optometry and Hospital Eye Services for referral of retinal disease enabled by a device-agnostic, tele-ophthalmology platform (superiority C-RCT).	Primary objective: To compare the proportion of referrals classified as unnecessary (cases that can be safely managed without a HES consultation) between current standard care and tele-ophthalmology digital referral pathway. Secondary objectives: To estimate the relative efficiency of the tele-ophthalmology digital pathway compared with standard care in both within trial-based evaluation. To compare the rate of inappropriate referrals (defined as wrong diagnosis or wrong level of urgency) between standard care and the tele-ophthalmology digital pathway. To capture the number of uncommon/complex retinal referrals to secondary care and the proportion that can be safely triaged through the tele-ophthalmology platform. To compare time from referral to review and/or treatment in HES for urgent referrals (such as Wet AMD and Retinal Vein Occlusions) between standard care and tele-ophthalmology digital pathway. To assess the number of false negatives (number of patients that would have benefited from a HES consultation but were deemed suitable for continued care in the community) (safety assessment)
2. To estimate the diagnostic (referral) accuracy and assess the ‘real-life’ performance of an Artificial Intelligence Decision Support System (the Moorfields-DeepMind AI) in the context of referral pathways between community optometry and HES.	To estimate the diagnostic (referral) accuracy of the Moorfields-DeepMind AI for recommending referral to HES from community optometry practices. To estimate the diagnostic accuracy of the Moorfields-DeepMind AI for the diagnosis of retinal disease. To assess the cost-effectiveness of the introduction of the DeepMind algorithm in the referral pathway between community optometry and HES. To assess the technical feasibility of using the Moorfields-DeepMind AI for real-time analysis of retinal OCT scan images. To assess real-time operational performance of the Moorfields-DeepMind AI in the tele-ophthalmology referral pathway.
3. To assess patient and healthcare professional acceptability as well as the barriers and enablers for the adoption of these digital technologies in the context of referral pathways between community optometry and HES through a human–computer interaction approach.	To understand current workflows and practices of staff and patients in community optometry and HES so as to identify key user requirements for tele-ophthalmology tools from the perspectives of both practitioners and patients (working with care settings with diverse established practices). To oversee the deployment of a digital referral platform at selected participating sites to ensure acceptability and acceptance by all user groups, and to understand the adoption process. To identify factors that shape professionals’ and patients’ attitudes to, and trust in, the Moorfields-DeepMind AI, and how to present information in ways that instil appropriate confidence. To observe workflows and practices of staff and patients in community optometry practices and HES with already established tele-ophthalmology pathways, aiding identification of technical, logistical and human factors affecting implementation of tele-ophthalmology in real-life (pragmatic sub-study).
4. To estimate the effectiveness and efficiency of a digital referral pathway between community optometry (High Street Opticians) and the Hospital Eyes Services for referral of retinal diseases enabled by a tele-ophthalmology platform in a real-life, observational post-implementation sub-study.	To compare the proportion of referrals classified as unnecessary (cases that can be safely managed without a HES consultation) against Reference Standard and the intervention arm of the RCT. To compare the rate of inappropriate referrals (defined as wrong diagnosis or wrong level of urgency) against the Reference Standard and the intervention arm of the RCT. To assess the number of false negatives (number of patients that would have benefitted from a HES consultation but were deemed suitable to continued care in the community) (Safety assessment). To compare time from referral to review and/or treatment in HES for urgent referrals (such as Wet AMD and Retinal Vein Occlusions) between post-implementation real-life tele-ophthalmology digital pathway and the intervention arm of the RCT. To estimate the relative efficiency of the real-life tele-ophthalmology digital pathway compared with the RCT tele-ophthalmology pathway.

AMD, Age-related Macular Degeneration; C-RCT, cluster randomised clinical trial; HERMES, Teleophthalmology-enabled and artificial intelligence-ready referral pathway for community optometry referrals of retinal disease: a cluster randomised superiority trial with a linked diagnostic accuracy study; HES, Hospital Eye Services; OCT, optical coherence tomography.

## Methods and analysis

### Study design

#### Superiority cluster randomised clinical trial of tele-ophthalmology pathway

An interventional superiority cluster randomised trial (RCT) will be performed comparing standard practice for referral of suspicious retinal disease with teleophthalmology between community optometry and HES. This part of the study will be reported according to the Consolidated Standards of Reporting Trials extension for cluster randomised clinical trial (C-RCTs).^10^

#### Diagnostic accuracy study of AI DSS

A prospective study will be conducted using the data of the above RCT to assess the diagnostic (referral) accuracy of an advanced AI DSS (the Moorfields-DeepMind Algorithm) for the automated diagnosis and referral recommendation for retinal disease. OCT scans transferred to the Moorfields Reading Centre in the course of the study will be assessed by the DeepMind algorithm in ‘real-time’ and its referral recommendations will be recorded and analysed for diagnostic (referral) accuracy and compared against the performance of human experts in the standard care and teleophthalmology arms of the RCT. This part of the study will be reported according to the STARD (Standards for Reporting Diagnostic accuracy studies) 2015 statement,^11^ or STARD-AI.^12^ A within-trial-based economic evaluation will estimate the efficiency of alternative referral models for retinal disease. A HCI analysis using qualitative methods will assess feasibility of implementation of both digital technologies.

#### Observational, postimplementations, pragmatic substudy

Community optometry practices in the Greater Manchester area will continue to refer patients with suspicious retinal disease to HES using the locally established teleophthalmology digital pathway. Referral recommendations will be compared against a reference standard provided by Moorfields Reading Centre to inform the assessment of real-life effectiveness and efficiency of the teleophthalmology referral pathway.

### Setting

For the C-RCT and diagnostic accuracy study, patients will be recruited at 24 optometry practices (clusters) in the catchment areas of four HES sites: Moorfields Eye Hospital NHS Foundation Trust (8–10 practices), Birmingham University Hospitals NHS Foundation Trust (4–6 practices), Central Middlesex Hospital at London North West University Healthcare NHS Trust (4–6 practices) and North West Anglia NHS Foundation Trust (4–6 practices). Twelve clusters (each cluster is an optometry practice) will be randomised to standard care and 12 clusters to the intervention (teleophthalmology). This selection of sites includes urban, suburban and rural locations within the UK allowing the inferences made from this study to be applicable to more of the UK population. Two additional optometry practices (clusters) will be randomised (1:1) in a reserve capacity in case of a cluster drop-out or in order to accelerate the recruitment process (online supplemental appendix 1)

10.1136/bmjopen-2021-055845.supp1 Supplementary data

For the pragmatic, observational, postimplementation substudy, patients will be recruited at 12 optometry practices (clusters) in the catchment area of Manchester University NHS Foundation Trust. These practices have adopted a teleophthalmology referral pathway as standard practices.

The enrolment start date is September 2020 with anticipated primary completion date in August 2023. Eligible practices need to have OCT devices and the activity volume and track record of referral to HES that will allow achieving the per practice recruitment target.

All OCT scans and clinical vignettes from each case will be transferred to the Moorfields Reading Centre that will provide the reference standard (diagnosis and referral recommendation). All suitable OCT scans will be processed by the DeepMind algorithm at the Moorfields Reading Centre in ‘real-time’ for the AI diagnostic accuracy study.

### Participants

Adults (≥18 years) attending for an eye examination at the participating community optometry practices who undergo an OCT scan will be considered for participation in the study. Only people with a suspicion of retinal disease in the opinion of the community optometrist will be recruited in the RCT and diagnostic accuracy study. As entire optometry practices (clusters) will be randomised into standard care or teleophthalmology, patients who are approached and agree to take part in the study will consent (online supplemental appendix 2) to data collection and analysis—there will not be patient-level randomisation. The patient-level inclusion criteria are outlined in box 1.

Box 1 **Patient-level selection criteria**

**Inclusion criteria**

- Able to give consent and understand the study.
- Able to cooperate by following study-specific instructions.
- Adults (≥18 years) attending the involved community optometry practices who underwent an optical coherence tomography (OCT) scan.
- Individuals who at the opinion of the community optometrist have any suspicion of a retinal condition (including atrophic (‘dry’) age-related Macular Degeneration (AMD), wet AMD, diabetic retinopathy, macular oedema, macular holes, epiretinal membranes, central serous chorioretinopathy, genetic eye disease).
- Macular OCT scan performed at community optometry.

**Exclusion criteria**

- Individuals with any non-retinal ocular comorbidities in either eye other than cataract.
- Individuals with media opacities, inability to position or fixate or any other reason that prevents acquisition of good-quality OCT scans (at the discretion of the community optometrist).

### Allocation to trial groups

Simple randomisation will be performed for involved optometry practices into the intervention and control arms. Randomisation will be performed with the unit of allocation being the cluster rather than the individual and allocation concealment will be at the cluster level. Optometry practices will be randomised 1:1 to standard care or teleophthalmology stratified by the hospital site. Optometry practices are committed to the allocated study arm for the duration of the recruitment period or until they have recruited the minimum of the per cluster recruitment range (10 patients).

### Superiority C-RCT of teleophthalmology pathway

**Figure 1:**
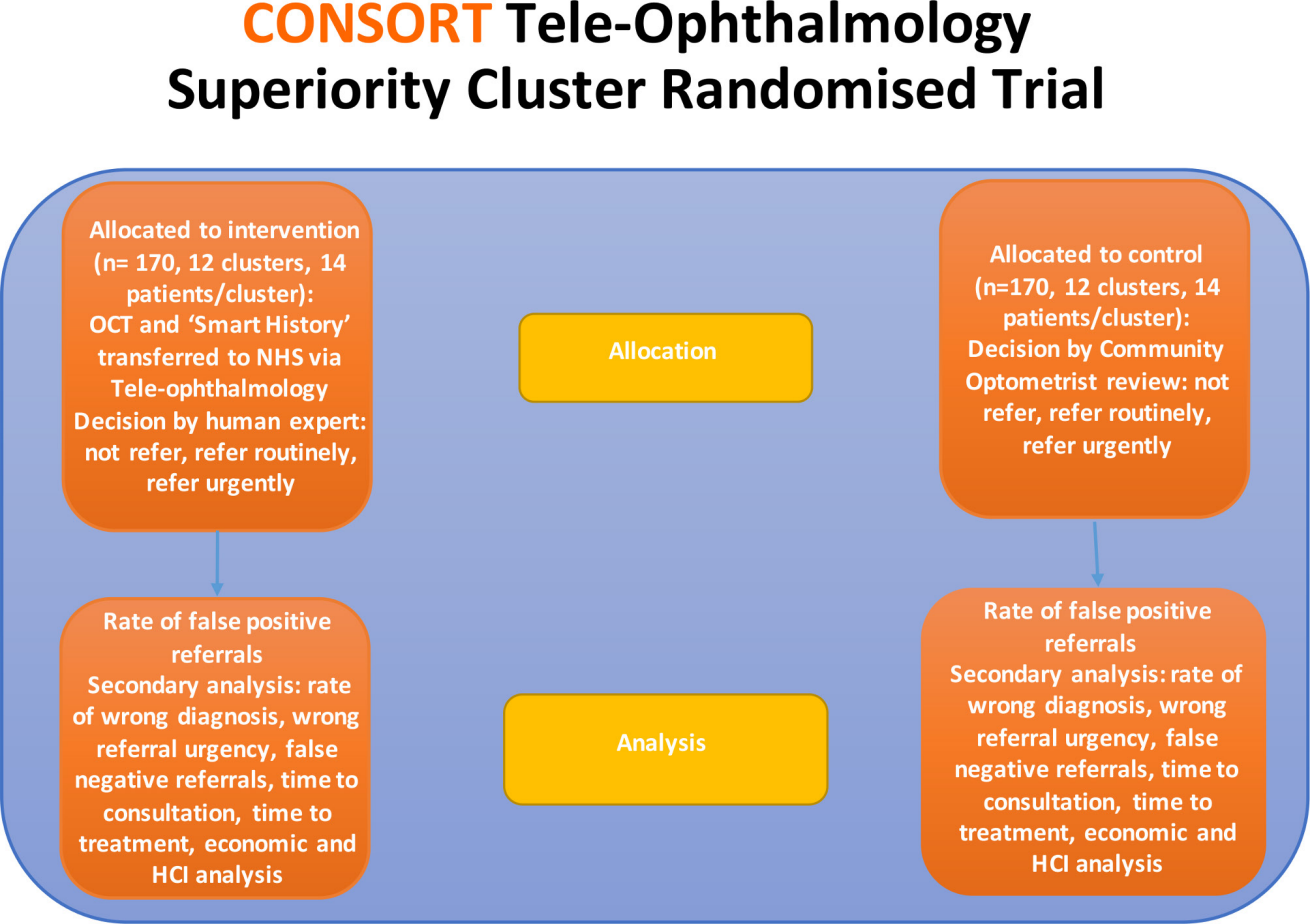
Superiority cluster randomised trial arms. HCI, human–computer interaction; OCT, optical coherence tomography.

Community optometry practices will be randomised to either continue with standard care for referral of retinal disease to hospital-based eye clinics or move to the teleophthalmology digital referral pathway (figure 1).

### Intervention arm for superiority C-RCT: digital pathway

The intervention pathway is the teleophthalmology model for referral of patients with suspicion of retinal disease from community optometry to HES using a digital referral platform. Patients who attend at participating community optometry practices will undergo a clinical assessment and OCT scan.

Participants’ OCT scan and clinical information will be transferred via the digital referral platform to corresponding HES. In each case, human expert clinicians based in HES (ophthalmologists or specialist optometrists with a minimum of 2 years experience of independent practice in the context of HES retinal clinics) will make a referral decision remotely (‘tele-HES’) after review of OCT scan and clinical information on the digital referral platform. A referral recommendation by the community optometrist will also be recorded but not acted on, to measure the proportion of correct/incorrect referrals in each arm (online supplemental appendix 3).

The remote review of OCT scans and clinical data at ‘tele-HES’ will be performed by expert clinicians (medics or specialist optometrists) experienced in retinal clinics (minimum of 2 years experience of independent practice in the context of retinal clinics in HES) based at Moorfields Eye Hospital, Central Middlesex Hospital, North West Anglia NHS Foundation Trust Hospitals or Queen Elizabeth Hospital Birmingham with access to senior advice by Consultant Ophthalmologists specialising in retinal disease.

### Control arm for superiority C-RCT: standard practice

The control pathway is standard practice for referral of patients with suspicion of retinal disease from community optometry to HES. Patients who attend a participating community optometry practice will undergo a clinical assessment and OCT scan. Patients with a suspicion of any retinal disease in the opinion of the community optometrist will be included in the study and will receive a referral decision (refer urgently to HES; refer routinely to HES; don’t refer to HES) by the community optometrist. All OCT scans and a clinical vignette from each case will be transferred to the Moorfields Reading Centre that will provide the reference standard for referral recommendations.

### Diagnostic accuracy study

**Figure 2:**
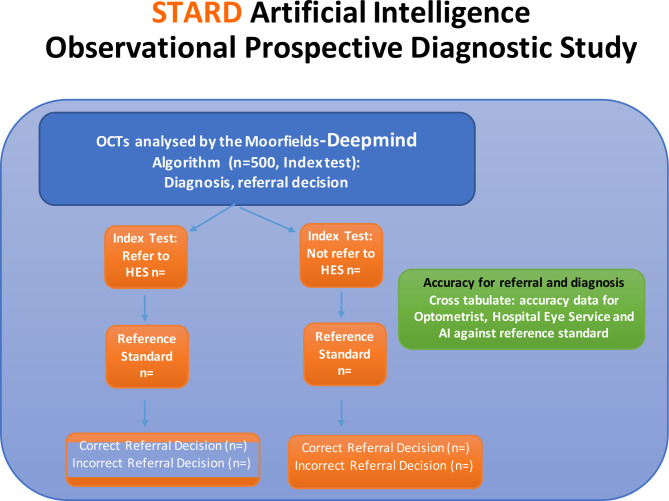
Diagnostic accuracy study arms. HES, Hospital Eye Services; OCT, optical coherence tomography; STARD, Standards for Reporting Diagnostic accuracy studies.

All referred and non-referred cases (from the standard care and teleophthalmology arm above; figure 2 will be included in the AI diagnostic study. All suitable OCT scans will be transferred prospectively on a weekly basis to the Moorfields Ophthalmic Reading Centre. OCT scans will be processed by the Moorfields-DeepMind AI system and end-to-end timing of the process will be captured for each case. For each case, the Moorfields-DeepMind AI will provide a:

1. Diagnosis.
2. Decision to refer or not.
3. Urgency of referral (routine or urgent).

#### Safety net

In cases where ‘tele-HES’ decision is ‘don’t refer’, the patient will be provided with additional information and alerts for clinical symptoms that should prompt a visit directly to the Accidents and Emergencies (A&E) department of the corresponding secondary care site (Moorfields Eye Hospital, Central Middlesex Hospital, North West Anglia NHS Foundation Trust Hospitals or Queen Elizabeth Hospital Birmingham). Additionally, in cases where a disagreement is found between the decision by the community optometrist and the one made in ‘tele-HES’, patients will be offered a follow-up appointment at the community optometry practice within 4 weeks.

#### Standard

OCT scans from the standard care and teleophthalmology arms will be transferred to the Moorfields Ophthalmic Reading Centre. The reference standard will be provided by the expert Ophthalmic Reading Centre for the C-RCT, diagnostic accuracy study and the pragmatic substudy. The reference standard will be the referral decisions and disease diagnosis made at the reading centre on the basis of review of images and clinical history and will apply to the RCT, the AI diagnostic accuracy study and the postimplementation, pragmatic substudy. Specifically, for each patient, the OCT scan (including b scans and colour fundus image) and a clinical vignette including visual acuity, age, symptoms, ocular and systemic history will be reviewed by two expert graders. The process to be followed is double grading with adjudication by a senior retinal specialist at the Moorfields Reading Centre.

#### Outcome measures

The outcomes for the superiority C-RCT and diagnostic accuracy study are outlined in table 2.

**Table 2:** Study outcomes

Superiority C-RCT	Diagnostic accuracy study	Pragmatic sub study
Primary outcome C-RCT: Proportion of false-positive referrals in the current referral pathway and the tele-ophthalmology referral pathway.	Primary outcome diagnostic accuracy study: Diagnostic accuracy of the referral decision made by the Moorfields-DeepMind AI (refer to HES, do not refer to HES) against the Reference Standard (Moorfields Reading Centre).	
Secondary outcomes C-RCT: Proportion of wrong diagnosis and wrong referral urgency in standard and tele-ophthalmology pathways against the reference standard Proportion of false-negative referrals, as well as sensitivity and specificity in standard and tele-ophthalmology pathways against the reference standard Time from referral to consultation for urgent and routine referrals in standard and tele-ophthalmology pathways Time from referral to treatment for urgent maculopathies in standard and tele-ophthalmology pathways Number of uncommon referrals (rare disease) that can be safely triaged in the tele-ophthalmology pathway Within trial cost-effectiveness and cost-consequences of the tele-ophthalmology digital pathway compared with standard care Modelled cost-consequences and net benefits of alternative diagnostic and referral strategies	Secondary outcomes diagnostic accuracy study: Sensitivity and specificity of Moorfields-DeepMind AI for the diagnosis of retinal disease Sensitivity and specificity of Moorfields-DeepMind AI for referral urgency Proportion of false-positive referrals in the standard and tele-ophthalmology pathways when human assessors are replaced by the AI DSS Proportion of wrong diagnosis and wrong referral urgency in the standard and tele-ophthalmology pathways when human assessors are replaced by AI DSS Uptime and end-to-end inference speed of technical infrastructure supporting the AI DSS Average time of end-to-end output (referral recommendation) by the AI DSS Cost-consequences and net benefits of AI-enabled digital referral pathway	Secondary outcomes of pragmatic sub-study: Proportion of false-positive referrals in the tele-ophthalmology referral pathway against the Reference Standard and the intervention arm in the main RCT. Proportion of wrong diagnosis and wrong referral urgency in the tele-ophthalmology pathway compared against the Reference Standard and the intervention arm in the main RCT study Proportion of false-negative referrals compared against the Reference Standard and the intervention arm in the main RCT study Time from referral to review and/or treatment in HES for urgent referral) in the post-implementation real-life tele-ophthalmology digital pathway

AI DSS, artificial intelligence decision support system; C-RCT, cluster randomised clinical trial; HES, Hospital Eye Services.

#### Patient and public involvement

Patient and public members were consulted prior to the trial protocol design on perception of teleophthalmology and issues of data privacy, impersonal care, trust in the technology, confidence in the quality of care provided through digital means were explored. A patient and public involvement (PPI) group based at Moorfields Eye Hospital was consulted during the trial design to advise on barriers to recruitment, issues around geographical spread of study sites and patient information material. After study commencement, the PPI group is planned to meet yearly and an end-of-study debrief is planned with all PPI contributors, which will include discussions of the prioritisation and dissemination of study results both to the public as well as relevant healthcare professionals.

## Data analysis plan

### Statistical analysis

In the superiority C-RCT of teleophthalmology pathway study, the primary analysis will be conducted following an intention-to-treat principle, where all randomised patients are analysed in their allocated group whether or not they receive their randomised management plan. The primary outcome is the proportion of false referrals, measured at the patient level. This will be compared between management groups using logistic regression adjusting for clustered centres. Outcomes will be reported as adjusted ORs.

In the AI diagnostic accuracy study, we will report estimates of sensitivity and specificity of the DeepMind algorithm for referral decisions with 95% CIs. Our primary analysis will combine urgent and standard referral to HES and compare against no referral to HES, but a sensitivity analysis will be conducted to evaluate urgent referrals. The full statistical consideration and analysis is provided in online supplemental appendix 4.

### Implementation science study components

#### Focus of analysis

The primary focus for analysis will be on facilitators and barriers to implementation of the teleophthalmology system and the introduction of AI decision support across clinical contexts, along with accounts of how it changes workflow and patient experience. Evaluation will be formative, so as to inform future implementations and also to contextualise the analyses of clinical effectiveness and cost-effectiveness. For the AI DSS, questions to be included in interviews will involve whether the AI is to be used as decision aid (eg, as a filter for disease/no disease) or as a completely independent decision-making tool, issues around trust in the technology, perceptions of medicolegal concerns (who is responsible for the decisions?), the optimal place in the care pathway for positioning the AI (high street optician or hospital-based eye services or both), concerns such as deskilling of practitioners (as diagnostic decisions may be devolved to AI), reduced employment opportunities, the need for a ‘safety net’/quality check to oversee and ‘sanity check’ the performance of the AI system and impersonal care for patients and perceived benefits such as more efficient and appropriate care, greater confidence in the process, etc. We will also particularly focus on the question of ‘interpretability’ of AI DSS and the ‘black box’ phenomenon and whether it influences trust and potential uptake of this technology. The ‘interpretability’ of AI DSS is a major factor in technology uptake and may influence the direction of AI DSS developers towards more interpretable technologies.

The data collected from sites with established teleophthalmology pathways (Greater Manchester) will be particularly valuable for identifying barriers to implementation in a real-life context that would not be picked up in the controlled environment of the RCT such as technical, staffing, training and human factors. Such barriers can be consequential with respect to patient safety as they have the potential to lead to delays in clinical review or missed cases and, therefore, the postimplementation substudy offers an opportunity to explore potential safety signals of the teleophthalmology pathways not typically observable in the context of RCT trials.

#### HCI analysis

As noted above, the aims of the HCI analyses are to assess the barriers and enablers for the adoption of the proposed digital technologies in the context of referral pathways between community optometry and HES through a HCI approach.

In order to capture patient and staff perspectives of teleophthalmology models of care as well as AI DSS, we will take a qualitative approach, conducting interviews and observations in both community optometry and HES. A full account on sampling, recruitment and analysis methods is provided in online supplemental appendix 1. We will compare people’s expectations (what they believe they will want and use) with their experiences when they have access to the relevant technology. In order to compare expectations against experiences, we will gather data in a variety of settings over the course of the project:

- *In the first 6* months of the project, the focus will be on understanding the adoption process and factors that contribute to success in adoption. Longitudinal data will be gathered at three selected sites: two optometry practices and the Birmingham HES. Data gathering will focus on expectations and current work practices before implementation; barriers, facilitators and experiences during implementation and perceptions and practices postimplementation.
- *Over the subsequent 12* months (months 7–18), similar data gathering and analysis methods will be adopted in two optometry practices that are already experienced in using teleophthalmology (sites that have already adopted teleophthalmology in the Greater Manchester area); two practices that are not using teleophthalmology and have no immediate plans to transition (control sites for the quantitative studies described above) and a second HES (Moorfields Eye Hospital). The focus will be on understanding workflows, practices and user requirements, including facilitators and barriers to adoption; and identifying factors that shape attitudes to the AI DSS, and how to present information to instil appropriate confidence.

#### Economic analysis

##### Cost-consequence analysis

The economic evaluation will comprise a within trial cost-consequence analysis (CCA) directly comparing the teleophthalmology pathway with the current referral pathway. This analysis highlights the choices and trade-offs between the modalities of care provision without an explicit synthesis of data into a single measure of efficiency. The results for the CCA will be presented as a balance sheet, which will include point estimates and appropriate measures of variance. From an NHS perspective, costs such as hospital visits, medications and community General Practitioner (GP) visits will be costed. Unit costs for resource use will be derived from published sources, eg, NHS Reference Costs and Unit Costs of Health and Social Care.^13^ When considering the addition of the community optometry perspective, the costs to purchase and maintain an OCT scanner will be considered. The costs of acquisition will be derived from market prices and converted into a cost per patient using standard economic methodology.^14^ In addition to this, the costs of the Moorfields-DeepMind algorithm will be considered within trial analysis. We will base this cost on advice from the algorithm owners as well as consideration of analogous algorithms. We expect there to be considerable uncertainty around the price to the NHS as a market price is not available. Therefore, we will explore the impact on efficiency of a range of prices. This will help decision-makers consider the maximum price they might be willing to pay for this algorithm given the benefits it may provide. In addition to the costs of running the algorithm in terms of hardware, software and staff, required will be considered. This will be based on its use within the study and advice from members of the study. A sensitivity analysis will be carried out to explore how the adoption of different perspectives (ie, who is bearing the costs) will affect the cost-effectiveness of the intervention. Outcomes which may be included in this CCA are false positives, false negatives, unnecessary hospital visits and duration of the time spent with an untreated macular disorder. These will be compared with the costs of provision and of the intervention and with the results of the DCE described below. Costs that will be included will be those that fall on the NHS and community optometry practices. Deterministic sensitivity analysis for example, variations in unit costs, will also be conducted. The consequences for each of the comparators will be based on a further consideration of outcomes (for example necessary referrals missed, correct referrals, individuals correctly not referred). The likelihood of these different outcomes (given as percentages) will be described.

To include insights derived from the pragmatic post-implementation study, the economic evaluation will also provide the following additional elements:

1. The Manchester sub-study group will inform estimates of the cost of the intervention, as delivered in a ‘real world’ application which may be more realistic than those estimated from a trial setting.
2. The Manchester sub-study group will be used to inform an exploratory analysis. In this, the costs and consequences of the real life sub-study group will be compared with the results from the trial group to identify if there is any meaningful difference between the two sets of data and identify what the driving factors are. As a safety analysis will be carried out as part of a sub-study, the cost and consequences of any unexpected adverse events that are recorded will be included in the cost consequence analysis. If any safety events become apparent during the design of the DCE then these may be used as the basis of different attributes and levels in the study design.

#### Discrete choice experiment

In addition to the CCA economic evaluation a DCE will be carried out to assess preferences of the general public about the teleophthalmology pathway. A DCE is an attribute-based survey method for measuring benefits. It offers participants at least two alternative choices, which vary across several attributes of interest. These can include several attributes of how the intervention is provided and its effect on health and other outcomes. Each of these attributes can vary over a range of levels. The choice of DCE attributes for this intervention will be informed from the existing literature on macular disease and provision of eye care service. The output of the qualitative study will also be examined for any attributes, which could affect the preferences of the users of the service. The DCE will also be used to value in monetary terms the relative importance of the different consequences included in the CCA. To do this, it will use methods previously successfully used in other NIHR-funded studies.^15 16^ The results of this monetary value will be used to inform a further cost benefit analysis.

#### Cost-benefit analysis

The results of the DCE will be used to value the outcomes described in the CCA. Outcomes will be expressed as a net monetary benefit by combining the differences in each outcome by the willingness to pay (WTP) for a unit change in that outcome. These values are known as WTP values and will be derived from the DCE described above. Costs will be included as part of the attributes that participants consider so that participants can express their WTP values for a described outcome. The cost of providing the outcome will be derived from the unit costs described earlier. A net benefit in monetary terms for these outcomes will be derived be subtracting the cost of the outcome from the WTP value (including a negative net benefit).

The values for the cost attribute will be based on pilot work and reviews of prior studies in this area, for example, Burr *et al* valued an intervention to monitor ocular hypertension to prevent glaucoma using a DCE. The range of values was between £15 and £70 (GBP 2012). Similarly, Shih *et al* assessed the WTP for a diabetic retinopathy screening service and reported a narrower range of between US$4 and US$24 (USD 2007).^17^ After the attributes have been established, then the piloting stages will occur. A survey company will be utilised to gain a large enough sample (www.researchnow.co.uk). The participants will be offered a small incentive (£1–£2) to complete the survey. The overall sample will be representative as closely as possibly for factors such as age, sex and ethnic background for the UK population. Optimal sample size requirements for the limited dependent variable models estimated in DCEs depend on knowledge of the true choice probabilities, which are not known prior to undertaking this research. However, previous DCE studies have shown that robust choice models can be estimated from sample sizes between 50 and 100 respondents. As such, a small pilot sample of 100 participants will be used as a sample to monitor the rate of completion and to carry out preliminary analysis and change any parts of the survey that are necessary. After the preliminary analysis is carried out, then a further sample of 300 participants will be surveyed, which will be sample size comparable to other HTAs in this area. The results of the DCE will be analysed using conditional logit regression analysis, which will measure the direction and strength of the participant preferences. Subgroup analysis will also be carried out to see if factors such as age, sex or ethnic background have any effect on the resulting preferences. Probabilistic and deterministic sensitivity analyses will be carried out to vary parameter uncertainty for both the costs and the effects. Results of the probabilistic sensitivity analysis will be presented as point estimates of net benefits, plots of costs and benefits net benefit curves, which show the likelihood of each intervention being most likely to have the highest net benefit.

### Dissemination of results

Findings will be reported through academic journals in ophthalmology, health services research and HCI; some will focus on findings from the HCI studies, and some will relate findings to those of the parallel studies covering other themes. PPI workshops will be organised in Birmingham and London before the main study period, with a focus on designing the study adapting the model from an earlier project (the ‘before’ study is reported by Furniss *et al*).^18^

## Ethics and dissemination

The research project will adhere to the UK Framework for Health and Social Care research. Ethics approval has been obtained for this project. No particular challenges are expected given the low-risk nature of the intervention of the RCT, the safety net arrangements for cases not referred to HES from community optometry, the observational design of the AI diagnostic accuracy study and the relatively low personal sensitivity of the topics to be investigated in the HCI studies.

With respect to confidentiality of patient records and case report forms (CRF; online supplemental appendix 5), identifiable patient data will not be accessed outside the care team without prior consent at any stage of the project. The OCT scans will be pseudoanonymised and no personal data will be included on the scans. No personal identifiers such as the patients’ name will be sent to the sponsor, and a unique identification code will be assigned to each OCT scan. This log of subject codes will be kept at each research site but not shared with the sponsor. Regarding data handling, the senior data manager in Moorfields Eye Hospital CRF will independently ask the IT application team to run missing data query and perform range check, logic check and data quality checks of the Electronic Database on a monthly basis. Active project data are stored in the dedicated secure Reading Centre drive with appropriate back up arrangements. Access to the drive is restricted only to Reading Centre staff with permission and access will be monitored, granted, revoked on a per user basis. This means that only individuals with prior authorisation can access the data. Further details on the handling of trial documents and subject records are found in appendix 6.

### Planned outputs

- High-impact peer-reviewed publications in Ophthalmology and Health Service Research journals.
- Presentations in conferences, including the Royal College of Ophthalmologists, the Association for Research in Vision and Ophthalmology, the American Academy of Ophthalmology conference, The College of Optometrists.
- The output of this programme has the potential to influence the healthcare landscape for eye care by validating digital care pathways for patients with retinal disease. The outcomes of this research will be communicated to NHS England and NHSx to inform policy on the role of digital technologies, including telemedicine and AI DSS.

The research team and the sponsor will actively approach and engage key parties such as the

College of Optometrists, stakeholders in community optometry and Clinical Commissioning Groups.

A detailed engagement plan will be formulated to disseminate the results of this research in order to inform policy decisions for optimising patient care.

## Supplementary Material

Reviewer comments

Author's manuscript
