# Supplementary material for: Teleophthalmology-enabled and artificial intelligence-ready referral pathway for community optometry referrals of retinal disease (HERMES): a Cluster Randomised Superiority Trial with a linked Diagnostic Accuracy Study—HERMES study report 1—study protocol
Source: BMJ Open. 2022 Feb 1;12(2):e055845. doi: 10.1136/bmjopen-2021-055845 (PMC8808461; doi:10.1136/bmjopen-2021-055845)
Supplement: Supplementary data [file bmjopen-2021-055845supp001.pdf]

Appendix

Table of Contents

Appendix 1. Sampling, Recruitment and Analysis Methods
Appendix 2. Participant Information Sheet and Consent Form
Appendix 3. Digital Pathway Decisions
Appendix 4. Statistical Consideration
4.1 Sample Size Calculation
Recruitment plan
Pragmatic Sub-Study
4.2 Statistical analysis
Appendix 5. Case Report Form
Appendix 6. Trial Documents and Subject Records
6.1 CRFs and Source Document Identification
6.2 Confidentiality of Trial Documents and Subject Records
6.3 Procedures for validation and securing of electronic clinical data systems
6.4 Data handling and record keeping
Appendix 7. References

Appendix 1. Sampling, Recruitment and Analysis Methods

Purposive sampling techniques will be applied to recruit participants who are representative of all key patient and professional groups across all sites, including both “power users” and reluctant users. In each setting, the aim will be to interview 15 patients and up to 10 clinicians and other professionals (settings: secondary care clinics; pre-; and post-transitioning optometry practices), depending on the sizes of the clinics selected for inclusion. The interviews will be conducted by a Process Evaluation Specialist, they will be semi-structured and will be designed to address the research objectives outlined above. Probes such as anonymised screenshots from the digital referral platform and illustrative information presentation prototypes from the DeepMind algorithm will be used in interviews to support the exploration of the themes. Interview data will be transcribed and analysed by a qualitative methods expert using inductive Thematic Analysis, with a particular focus on facilitators and barriers to change, and the factors that contribute to successful change. These will include questions around trust in technology and data privacy as well as efficiency and effectiveness and changes in clinician workflow and patient experience. Data gathering and analysis will be interleaved, so that later data gathering is informed by the findings from earlier analysis.

Small-scale ethnographic observations will be conducted in all settings, observing both selected clinician-patient interactions around the diagnostic process (community optometry and HES) and clinician tele-care practices (HES). 3-5 clinician-patient consultations will be observed per setting; debrief interviews with patients will cover the same themes as the interviews with practitioners but be sensitive to the different perspectives of patients and professionals. Detailed field notes will be kept of all observations. This data will also be subjected to thematic analysis, focusing on workflows, variability in workflows, and any problems experienced during the interaction (particularly related to technology use).

Patients will be invited to participate at the time that they receive their appointment letter, so that they have time to consider whether they wish to do so (for informed consent), and to plan their clinic visit time to accommodate a short interview (15 mins approx.) after their appointment. On the day of the visit the investigator will provide the patient information leaflet (PIS; **Appendix 1**) to the patient and go through it highlighting what the purpose of the study is, what it entails if the patient decides to take part and possible advantages and disadvantages and risks of taking part. When the patient has had amble time to read the PIS and ask questions regarding the study, the patient will be asked to sign an informed consent form (ICF; **Appendix 1**). Once the informed consent process is complete a copy of the ICF will be provided to the participant, and the signed form will be filed in the participant’s study records. Once the informed consent process is complete, the investigator will record the decision in the case history form.

As this is a cluster randomised clinical trial, randomisation applies at the level of entire community optometry practices. The practices randomised to the intervention arm (tele-ophthalmology) will adopt this pathway for all patient referrals to secondary care as standard practice. Patient-level consent for this study pertains to allowing use of collected data for analysis but participation in the study will not

affect patient-level care. Given the urgent presentation of the patient population we will approach for participation in this study and the fact that patient management will not be influenced by randomisation as described above, a 24-hour minimum period of consideration for patient participation is not warranted. Patients approached for participation will be given the study-specific PIS and adequate time to have any queries addressed by the clinical team before deciding on participation to the study.

Appendix 2. Participant Information Sheet and Consent Form

Patient Information Sheet

Study title: Tele-op**H**thalmology- enabl**E**d and A**R**tificial Intelligence-ready referral pathway for co**M**munity optom**E**try referral**S** or retinal disease: acluster Randomised Superiority trial with a linked Observational Diagnostic Accuracy Study

Short title: HERMES

Protocol Reference Number: BALK1006

We would like to invite you to take part in our research study. Before you decide we would like you to understand why the research is being done and what it would involve for you. One of our team will go through the information sheet with you and answer any questions you have. We'd suggest this should take about 15 minutes. Talk to others about the study if you wish.

Part 1 tells you the purpose of this study and what will happen to you if you take part. Part 2 gives you more detailed information about the conduct of the study.

Ask us if there is anything that is not clear. Take time to decide whether or not you wish to take part.

Part 1

1. What is the purpose of the study?

Early diagnosis of retinal condition (including Age-related Macular degeneration) is classified using imaging technology called Optical coherence tomography (OCT). This technology is becoming more available in community based optometry practices (high street optometry practices); however, interpreting the imaging scans can require hospital level expertise.
As a result of this, a large proportion of patients with retinal disease are incorrectly referred to hospital based eye clinics for diagnostics and disease management. This has led to an increasing pressure on hospital eye services and can cause delay in access for patients with sight threatening disease.

In this study we will involve patients with suspected retinal condition who have attended an eye examination appointment at a participating community practice. Patients that want to take part will be referred to the hospital eye service via the standard pathway for either: urgent care, routine care or not referred at all depending on the clinical assessment and OCT scan taken by the community optometrist.

The study will seek to show that we can improve patient care by using tele-Ophthalmology technologies to manage the proportion of referrals that do not need to attend hospital eye service for consultations and can be managed safely by community based optometry practices. Half of the optometry practices involved in this study will do the referrals through a 'tele-ophthalmology' process. This means that your eye scans and other clinical information will be reviewed remotely by expert clinicians at corresponding NHS eye hospitals and they will make the referral decision instead of the optometrists.

Han JED, *et al. BMJ Open* 2022; 12:e055845. doi: 10.1136/bmjopen-2021-055845

Your eye scans may also be processed at a later time by an Artificial Intelligence algorithm that can read the scans and also make a referral decision. The Artificial Intelligence algorithm has been developed previously through the collaboration between Moorfields Eye Hospital and Google/Deepmind. This algorithm will be introduced in the Reading Centre (Moorfields Eye Hospital NHS Trust), all data analysis will be performed within the Reading Centre (Moorfields Eye Hospital NHS Trust) and no research data will be shared or analysed externally to the research team. During this study, the Artificial Intelligence algorithm will analyse the OCT and make a recommendation on whether a referral to the hospital is needed or not; this will then be compared with the referral recommendation made by an expert clinician for your care and will not impact on your care in any way. The actual decision to refer or not will be made by a human expert in every case and not by the algorithm.

Additionally, in a sub-study we want to involve patients with suspicion of retinal disease who are already being referred to the hospital eye services via a tele-ophthalmology platform. The patient's clinical history and OCT scan will be reviewed by experts at the participating hospital eye service and a referral decision will be made remotely. This sub-study is seeking to understand what impact the introduction of tele-ophthalmology has in a real-life setting where tele–ophthalmology is already used to refer patients to the hospital eye services and also in terms of patient care and managing unnecessary referral that are made by the community based practices to the hospital based eye services.

2. Why have I been invited?

You have been invited to take part in this study because you are attending an eye examination appointment at a participating community optometry practice that undergo OCT scans and have been diagnosed with suspicious retinal disease.

3. Do I have to take part?

It is up to you to decide to join the study. We will describe the study and go through this information sheet. If you agree to take part, we will then ask you to sign a consent form. You are free to withdraw at any time without giving a reason. This would not affect the standard of care you receive. If you decide not to take part in the study, you will still have your normal eye examination and an eye scan.

4. What will happen to me if I take part?

If you decide to take part in this study, you (and if applicable a witness) will be asked to sign and date a consent form. The consent form will be signed in the presence of a trained healthcare professional who will also sign and date it. You will be provided a copy of this to keep. After the consent process you will undergo a clinical assessment and have an eye scan (OCT). If you take part in the routine care arm of the study, a referral decision will be made as usual by your community optometrist who will decide whether you will need to be; referred urgently to a hospital eye service,

routinely or not referred at all. The referral decision will be sent as per standard practice to the corresponding hospital eye service. If you take part in the tele-ophthalmology arm of the study, expert clinicians at the corresponding NHS eye hospital will review your eye scan and other clinical information remotely and will make the referral decision.

You will not have a choice about which arm of the study you will participate in, as the optometry practice will already have been pre-assigned either the routine referral arm or the tele-ophthalmology arm. However this will not influence in any aspect the standard of care you will receive during this visit. You will receive the same care whether you are in the routine care arm or in the tele-ophthalmology arm.
Only your community optometrist or an expert clinician will make a referral decision after reviewing your eye scan and clinical information, however you will not be able to decide who will make the referral decision. Additionally, a computer program (AI) may analyse your eye scan and give its own clinical diagnosis, however this will not influence the referral decision that will be made. By using this computer program we only want to obtain information that will help clinicians to make a better clinical decision and diagnosis in the future for people with retinal disease.

If you decide to take part in the sub-study, you will undergo the consent process as described above. After the consent process you will undergo a clinical assessment and have an eye scan (OCT). Your community optometrist will make a referral decision as usual who will decide whether you need to be referred urgently to a hospital eye service, routinely or not referred at all. After reviewing your eye scan, your community optometrist will send the referral via tele-ophthalmology to the corresponding NHS eye hospital. Expert clinicians at the corresponding NHS eye hospital will review your eye scan and other clinical information remotely and will make the referral decision.

5. What are the possible risks and benefits of taking part?

This study has no invasive testing and no therapeutic interventions; therefore there is minimal risk to patients if they choose to participate in this study. Your eyes may be dilated during your visit for the OCT scan. This will make you sensitive to light, blur your vision and may make it difficult to focus on close up objects for 4-6 hours.

We cannot promise the study will help you, but the information we get from this study will help improve the experience of care of people with retinal disease and improve the referral pathway between community optometry practices and the hospital eye service.

6. What if there is a problem?

Any complaint about the way you have been dealt with during the clinical trial or any possible harm you might suffer will be addressed. The detailed information concerning this is given in Part 2 of this information sheet. If you have any concerns or complaints you should contact your study doctor in the first instance.

7. Will my taking part in the study be kept confidential?

Yes. We will follow ethical and legal practice and all information about you will be handled in confidence. The details are included in Part 2.

8. Contact Details

Principal investigator

Name: Tel. Number:

Research Project Manager

Name: Tel. Number:

This completes Part 1 of the Information Sheet.

If the information in Part 1 has interested you and you are considering participation, please read the additional information in Part 2 before making any decision.

Part 2

9. What if relevant new information becomes available?

On receiving new information, we might consider it to be in your best interests to withdraw you from the study. If so, we will explain the reasons and arrange for your care to continue. If the study is stopped for any other reason, we will tell you why and arrange your continuing care.

10. What will happen if I don't want to carry on with the study?

If you withdraw from the study, we will destroy all your identifiable information, but we will need to use the data collected up to your withdrawal.

11. What if there is a problem?

If you have a concern about any aspect of this study, you should ask to speak to the researchers who will do their best to answer your questions …. If you remain unhappy and wish to complain formally, you can do this by contacting the PALS team at ….
If taking part in this research project harms you, there are no special compensation arrangements. If you are harmed due to someone's negligence, then you may have grounds for legal action but you may have to pay for it. Regardless of this, if you wish to complain about any aspect of the way you have been approached or treated during the course of this study, the normal National Health Service complaints mechanisms will be available to you.

12. Will use of my data meet the GDPR rules?

Yes, all data will be handled in accordance with the General Data Protection Regulations (GDPR) and UK Data Protection Act 2018, The Research Governance

Framework for Health and Social Care and the conditions for the Research Ethics Committee favorable opinion.

Moorfields Eye Hospital NHS Trust is the Sponsor for this study based in the United Kingdom. We will be using information from you and/or your medical records in order to undertake this study and will act as the data controller for this study. This means that we are responsible for looking after your information and using it properly.

Your rights to access, change or move your information are limited, as we need to manage your information in specific ways in order for the research to be reliable and accurate. If you withdraw from the study, we will keep the information about you that we have already obtained.

You can find out more about how we use your information by visiting www.moorfields.nhs.uk/content/how-we-use-your-information or please contact your research team (study team contact details can be found in your participant information sheet).

(SITE NAME) will use your name, and contact details to contact you about the research study, and make sure that relevant information about the study is recorded for your care, and to oversee the quality of the study. Moorfields Eye Hospital and their study collaborators (which may include Universities) will receive information from your medical record for the purposes of the study but without your name or any other personal details. (SITE NAME) will pass to Moorfields Eye Hospital this information collected from you and/or your medical records. The only people in (SITE NAME) who will have access to information that identifies you will be people who need to contact you to arrange appointments or audit the data collection process. The people who analyse the information will not be able to identify you and will not be able to find out your name or contact details. Data collected for this study, without your name or any other personal details, can be used for future research. If you agree to take part in this study, we will also specifically ask for your permission to use your data for future research. If you don't want your data to be used for any future research, you can still participate in this study.

Moorfields Eye Hospital will keep identifiable information about you from this study without your personal details for 15 years.

13. Will my GP be informed of my involvement?

With your permission, your GP, and other doctors who may be treating you, will be notified that you are taking part in this study.

14. What will happen to the results of the research study?

The results of the study will be available after it finishes and will usually be published in a medical journal or be presented at a scientific conference. The data will be anonymous and none of the patients involved in the trial will be identified in any report or publication.

Should you wish to see the results, or the publication, please ask your study doctor.

15. Who is organising and funding the research?

The study is organized by Moorfields Eye Hospitals NHS Foundation Trust and funded by the National Institute for Health Research.

16. Who has reviewed the study?

All research in the NHS is looked at by independent group of people, called a Research Ethics Committee, to protect your interests. This study has been reviewed and given favourable opinion by London – Bromley Research Ethics Committee.

17. Further information and contact details

You are encouraged to ask any questions you wish, before, during or after your treatment. If you have any questions about the study, please speak to your study Optometrists or doctor, who will be able to provide you with up to date information about the procedure(s) involved. If you wish to read the research on which this study is based, please ask your study optometrists or doctor. If you require any further information or have any concerns while taking part in the study please contact one of the following people:

Principle Investigator

Name: Tel. Number:

Research Project Manager

Name: Tel. Number:

If you decide you would like to take part then please read and sign the consent form. You will be given a copy of this information sheet and the consent form to keep. A copy of the consent form will be filed in your patient notes, one will be filed with the study records and one may be sent to the Research Sponsor.

You can have more time to think this over if you are at all unsure.

Thank you for taking the time to read this information sheet and to consider this study.

Appendix 3. Digital Pathway Decisions

The following scenarios can occur in the intervention arm:

1. Community optometrist recommendation: **Refer urgently to HES**—> OCT scan and clinical data are transferred to 'tele-HES' and reviewed within 48h remotely by human expert —> Referral Decision is made in 'tele-HES' (refer urgently, refer routinely, don't refer) and fed-back to the community optometry practice to be implemented.
2. Community optometrist recommendation: **Refer routinely to HES**—> OCT scan and clinical data are transferred to 'tele-HES' and reviewed within 48h remotely by human expert —> Referral Decision is made in 'tele-HES' (refer urgently, refer routinely, don't refer) and fed-back to the community optometry practice to be implemented.
3. Community optometrist recommendation: **Don't refer to HES**—> OCT scan and clinical data are transferred to 'tele-HES' and reviewed within 48h remotely by human expert —> Referral Decision is made in 'tele-HES' (refer urgently, refer routinely, don't refer) and fed-back to the community optometry practice to be implemented.

Appendix 4. Statistical Consideration

The trial statisticians based at King's Clinical Trial Unit will write the statistical analysis plan before database lock and will perform the analysis using the Stata software (StataCorp, College Station, TX, USA).

4.1 Sample Size Calculation

The primary outcome is the proportion of false positive referrals. Under the current system an audit conducted at Moorfields Eye Hospital NHS Foundation Trust in September 2018 showed that 70 % of retinal referrals were false positive.[1] A pilot study on 40 patients conducted in three optometry practices showed that this could be reduced by 60 %.[1] A 95 % confidence interval computed by the modified Wald Method as advised by Agresti and Coull would extend 44.6% to 73.7%.[2] There is consensus amongst clinicians however that given the savings to the NHS and benefit to patients, slightly smaller differences would be important to detect and we have powered the study to examine a reduction to 40 % false referrals. Whilst smaller differences might yet be important it would seem unethical to power for lower than 40 % based on the observed data and clinical expertise in this area.

Although decisions for patients are made on an individual basis each patient cannot be assumed to generate independent information since they will be clustered within optometry practices. The correlation of information from patients within a cluster (the intracluster correlation) is estimated to be 0.15. We have based this intracluster correlation on previous work conducted in ophthalmology [3, 4] with a clinical outcome similar to this study. Since this is an estimate we have increased the intracluster correlation slightly to allow for the potential that patients within the same optometry practice may be more similarly managed than patients within different practices although clinical consensus is that clinical signs are more likely to impact upon decision making for referral than individual optometrist attitudes. Using nQuery software version 8.3.10, a hierarchical 2-level mixed effects model was used to calculate the required sample size. 24/26 clusters split between the study arms in a 1:1 ratio need to recruit an average of 12/10 patients per clusters (12 patients if 24 clusters, 10 patients if 26 clusters) in order to achieve 89.27% power to detect a difference in the proportion of false positive referrals of 30% (a drop from the current rate of 70% to the clinically relevant rate of 40%). This calculation assumes an intracluster correlation of 0.15 and the test is performed at the 5% significance level.

A total of 288 patients (based on an average of 12 patients recruited at 24 clusters, 144 per study arm) would therefore be needed to complete the data analysis with sufficient statistical power. To allow for an anticipated 15% drop our rate (patients are likely to be elderly and have comorbidity causing motion artefacts and some images may be ungradable), the total sample size is 340 patients (170 per study arm).

The sample size of the RCT and pragmatic sub-study combined will also enable the AI observational diagnostic accuracy study to obtain robust estimates of sensitivity and specificity. All 500 patients (accounting for the anticipated drop-out rate) will be included in the AI study. Classifications will be

made without additional clinical information. Research from our group suggests that the diagnostic accuracy of the Moorfields-DeepMind AI will be as high as 95%.[5] This combined sample of 500 patients with 475 patients being correctly diagnosed would produce a two-sided 95.0% confidence interval with a width of 0.039. The sample from the RCT alone -288 patients with 274 being correctly diagnosed - produce a two-sided 95.0% confidence interval with a width of 0.052. PASS has been used to calculated these widths.

Recruitment plan

On the basis of feedback provided by optometry practices already identified and interested in participating in the study, an average of 3 eligible patients can be approached to consider participation in the study per month per cluster, with a range of 2-5 patients based on the size of the optometry practice. However, it is also expected that 35% of potential patients will decline to participate. Currently, different sites are at different stages of readiness for commencing recruitment and therefore a staggered start to recruitment over 3 months is embedded in the recruitment plan. Based on these conservative estimates a recruitment period of 12 months with a staggered initiation over the first 3 months will be sufficient to approach 521 patients, of whom it is expected that 340 patients will be recruited to the study. A smaller practice only approaching 2 patients per month will require 11 months to recruit 12 patients (accounting for drop out and decline to participate). If the practice was one of the last to start recruitment and so started 3 months into the recruitment window, they would still manage to recruit 10 patients in 9 months (accounting for drop out and decline to participate). A larger practice in the same catchment area will be able to compensate by over-recruiting up to a set maximum of 16 patients. The range of cluster size will thus be 10-16 patients.

The 2 additional randomised community practices allow the potential to increase the number of clusters to 26 if further acceleration of recruitment is required. 26 clusters will be required to recruit 306 patients overall with an average of 10 patients per cluster (and a minimum of 8 patients per cluster) in order to achieve 89.27% power to detect a difference in the proportion of false positive referrals of 30% - using the same parameters as the sample size calculation for the 24 clusters.

Pragmatic Sub-Study

Manchester Eye Hospital and its local area, a site included in the original protocol, has already moved to a tele-ophthalmology referral pathway as part of a commissioning change across the local region. This change in standard care provides a unique opportunity to examine whether tele-ophthalmology works under usual conditions within the NHS. This sub-study will allow us to measure and visualise variation in quality of health care within a local region to inform our inferences from the RCT on how the tele- ophthalmology pathway will perform within a real-life setting.

From this study, key estimate statistics will be calculated including the overall rate of referral to HES, the false positive (referral) and false negative rate against the Reference Standard and the proportion of wrong diagnosis and wrong referral urgency. These shall be compared to the rates found for the intervention arm of the main RCT.

Recruiting 18 patients from each of 12 tele-optometry practices (for a total of 216 patients) will allow the proportion of false positive referrals to be produced with a 95% confidence interval with a width less than 0.187. This was calculated based on confidence intervals for one proportion within a cluster-randomised design with an intracluster correlation of 0.15. A total of 216 patients (based on an average of 18 patients recruited from 12 clusters) would therefore provide a certain degree of precision. To allow for an anticipated 15% drop our rate (patients are likely to be elderly and have comorbidity causing motion artefacts and some images may be ungradable), the total sample size is 254 patients. It is expected that 35% of participants will decline to participate and so 390 patients would need to be approached. These patients can be recruited over a period of 18 months with an average of 3 patients approached by each practice per month.

4.2 Statistical analysis

The primary analysis will be conducted following an intention to treat principle where all randomised patients are analysed in their allocated group whether or not they receive their randomised management plan. Baseline characteristics will be summarised for each management group (standard care or tele-ophthalmology). We will report the number of clusters in each group and the size of clusters. Continuous data will be summarised using means and standard deviations if data appear Gaussian or medians and interquartile ranges. Categorical data will be reported as proportions and percentages.

The primary outcome is the proportion of false referrals. The outcome is measured at the patient level. This will be compared between management groups using logistic regression adjusting for clustered centres. Outcomes will be reported as adjusted odds ratios. We will also report the difference in proportions with a 95 % confidence interval as per the CONSORT extension for cluster randomised controlled trials. We will report false referral rates with 95 % confidence intervals computed by the exact binomial method by diagnosis and by level of urgency. The results will be presented at the cluster level and overall.

Secondary outcomes such as time from referral to review in HES and treatment will be analysed in a similar fashion. The percentage of patients experiencing adverse events in the two groups will be reported with 95 % confidence intervals computed by the exact binomial method.

Loss to follow-up will be examined by study arm. Reasons for missingness may be important and these will be investigated using logistic regression of covariates based on an indicator of missingness. An available case analysis will be reported along with an analysis using imputed data based on best- and worst-case scenarios. Since this is a cluster RCT we will also examine and report missingness by cluster.

No formal interim analysis is planned but reports concerning patient safety will be prepared for review by the Independent Data Monitoring Committee. All tests will be two sided and will be assessed at the 5 % significance level unless otherwise specified. All confidence intervals will be 95 % and two sided. A detailed statistical analysis plan will be agreed with the Trial Steering Group prior to any analysis of locked data. All statistical analysis will be performed using Stata (StataCorp, College Station, TX, USA).

Statisticians analysing the data will be masked to the management group status of the practise and patient.

In the AI diagnostic accuracy study, we will report estimates of sensitivity and specificity of the DeepMind algorithm for referral decisions with 95 % confidence intervals. Our primary analysis will combine urgent and standard referral to HES and compare against no referral to HES but a sensitivity analysis will be conducted to evaluate urgent referrals. The referral outcome (refer routinely, refer urgently, don't refer) will be cross tabulated for the DeepMind algorithm and each of the RCT treatment arms (community optometry and 'tele-HES'), the pragmatic sub-study, and for the Reference Standard.

Appendix 5. Case Report Form

Study No: ☐☐ - ☐☐☐

HERMES Study
Completing Case Report Forms (CRFs)

This document has been created to provide guidelines about completing clinical trial case report forms at Moorfields Eye Hospital (MEH). The information has been extracted from the standard operating procedures (SOPs) 'Completing, Correcting & Signing off Case Report Forms (CRF_S07)' that have been developed by the Research & Development department at Moorfields.

1. The CRF must be completed as soon as possible after the patient has been assessed or during the assessment if the CRF is the source data.

2. CRFs must be completed using a black ink ballpoint pen.

3. If the CRF is printed on carbonless duplication paper, a suitable separator must be inserted under the form being completed.

4. Data entry into the CRF must be complete as without omissions. If data are unavailable then 'unknown', 'missing', 'test not done' etc. should be inserted. The ambiguous phrase, 'not available' should be avoided.

5. All entries into the CRF must be accurate, legible and verifiable with the source data in the medical records (unless the CRF is the source data). Data must not be invented – this is fraud.

   N.B. Whenever a subject has been seen by clinical staff for the purposes of a clinical trial, the time, date and reason for visit must always be entered into the subject's corresponding hospital notes. Copies of trial investigations/results that are clinically significant or have an impact on the patient's clinical care must also be filed in the medical notes.

6. Any discrepancies between the CRF and the source data should be explained and the significance noted in the CRF and/or patient's medical records.

7. All CRF data derived from source documents must be transcribed exactly.

8. For laboratory values that fall outside the laboratory's reference range or trial specific range or when a value shows a significant variation from one assessment to the next, this should be commented on and the significance noted in the CRF and/or patient's medical records.

9. The subject's identity should remain confidential at all times and as such the trial subject must only be identified in the CRF using a trial number or code.

10. Entries into the CRF must never be overwritten.

11. Corrections to the CRF must be made as follows:

    - An incorrect entry must be deleted with a single line through the text allowing the incorrect entry to remain legible. Correction fluid must never be used and entries must not be obliterated.
    - The correct data must be entered.
    - The correction must be initialled and dated and an explanation given of the correction, if applicable.

12. The CRF must be signed and dated where indicated, by the chief/principal investigator or designee (for example, research nurse at the end of an assessment) to assert that he/she believes the data is completed and correct.

Moorfields Eye Hospital, Case Report Form
BALK1006

Moorfields Eye Hospital NHS Foundation Trust NHS

Study No: ☐☐ - ☐☐☐

HERMES Study

PATIENT DEMOGRAPHICS (Community Optometry) **Page 1/2**

**ALL FIELDS ARE MANDATORY**			
**Patient Details**			
Study site	MEH ☐<br>Central Middlesex ☐	Manchester ☐	Birmingham ☐<br>North West Anglia ☐
Optometrist site			
Randomisation Cluster number			
Randomisation Arm	Control ☐		Intervention ☐
Age			(≥18) ☐☐☐
Sex	Male ☐		Female ☐
Medical history	Yes No<br>Heart attack ☐ ☐<br>COPD ☐ ☐<br>Diabetes ☐ ☐<br>Hypertension ☐ ☐		Yes No<br>TIA/Stroke ☐ ☐<br>Impaired Mobility ☐ ☐<br>Asthma ☐ ☐<br>Other ☐ ☐
If other please specify			
Medication for eye condition	Yes ☐		No ☐
If yes specify glaucoma drops?	Prostaglandins ☐	CA inhibitors ☐	B-blockers ☐
If yes specify AREDS	Yes ☐		No ☐
Smoker?	Ex-smoker ☐	Smoker ☐	Non-smoker ☐

	**Right Eye**	**Left eye**
Ocular history	Yes No<br>Wet AMD ☐ ☐<br>Dry AMD ☐ ☐<br>Central Serous Chorioretinopathy ☐ ☐<br>Central Retinal Vein Occlusion ☐ ☐<br>Branch Retinal Vein Occlusion ☐ ☐<br>Diabetic Macular Oedema ☐ ☐<br>Inherited Eye Disease ☐ ☐<br>Other ☐ ☐	Yes No<br>Wet AMD ☐ ☐<br>Dry AMD ☐ ☐<br>Central Serous Chorioretinopathy ☐ ☐<br>Central Retinal Vein Occlusion ☐ ☐<br>Branch Retinal Vein Occlusion ☐ ☐<br>Diabetic Macular Oedema ☐ ☐<br>Inherited Eye Disease ☐ ☐<br>Other ☐ ☐
If other, specify		

Han JED, *et al. BMJ Open* 2022; 12:e055845. doi: 10.1136/bmjopen-2021-055845

Moorfields Eye Hospital, Case Report Form
BALK1006

Moorfields Eye Hospital NHS Foundation Trust NHS

Study No: ☐☐ - ☐☐☐

HERMES Study

PATIENT DEMOGRAPHICS (Community Optometry) **Page 2/2**

ALL FIELDS ARE MANDATORY

**Patient Details**						
		Yes	No		Yes	No
Previous eye procedures	Cataract surgery	☐	☐	Cataract surgery	☐	☐
	Glaucoma surgery	☐	☐	Glaucoma surgery	☐	☐
	Eyelid surgery	☐	☐	Eyelid surgery	☐	☐
	Other	☐	☐	Other	☐	☐
If other specify						

Comments

I can confirm that the patient meets all eligibility criteria for the study and I have completed this form in full and take full responsibility for any missing data.

**Signed:**	**Print:**	**Date:**
		☐☐ / ☐☐ / ☐☐☐☐
**Office use only, data entry completed by:**		
**Signed:**	**Print:**	**Date:**
		☐☐ / ☐☐ / ☐☐☐☐

Han JED, *et al. BMJ Open* 2022; 12:e055845. doi: 10.1136/bmjopen-2021-055845

Moorfields Eye Hospital, Case Report Form
BALK1006

Moorfields Eye Hospital NHS Foundation Trust NHS

Study No: ☐☐ - ☐☐☐

HERMES Study

BASELINE VISIT (Community Optometry) **Page 1/2**

**ALL FIELDS ARE MANDATORY**				
**Baseline Exam**				
Date of visit	*(dd mm yyyy)* ☐☐ / ☐☐ / ☐☐☐☐			
Years since diagnosis of any retinal disease?	☐☐			
OCT Device	Topcon 3D OCT-2000 ☐ Heidelberg OCT1 ☐ Other ☐			
If other specify				
	**Right Eye**		**Left eye**	
Visual acuity (ETDRS)	*(ETDRS letters, range 0-95)* ☐☐		*(ETDRS letters, range 0-95)* ☐☐	
Visual acuity (Snellen)	☐ / ☐☐		☐ / ☐☐	
Diagnosis by the Optometrist		Yes / No		Yes / No
	Wet AMD	☐ ☐	Wet AMD	☐ ☐
	Dry AMD	☐ ☐	Dry AMD	☐ ☐
	Central Serous Chorioretinopathy	☐ ☐	Central Serous Chorioretinopathy	☐ ☐
	Central Retinal Vein Occlusion	☐ ☐	Central Retinal Vein Occlusion	☐ ☐
	Branch Retinal Vein Occlusion	☐ ☐	Branch Retinal Vein Occlusion	☐ ☐
	Diabetic Macular Oedema	☐ ☐	Diabetic Macular Oedema	☐ ☐
	Inherited Eye Disease	☐ ☐	Inherited Eye Disease	☐ ☐
	Other	☐ ☐	Other	☐ ☐
If other specify				
Clinical findings		Yes / No		Yes / No
	Macular Haemorrhage	☐ ☐	Macular Haemorrhage	☐ ☐
	Other RetinalHaemorrhage	☐ ☐	Other RetinalHaemorrhage	☐ ☐
	Exudates	☐ ☐	Exudates	☐ ☐
	Disc Swelling	☐ ☐	Disc Swelling	☐ ☐
	Macular Atrophy	☐ ☐	Macular Atrophy	☐ ☐
	Cotton Wool Spot	☐ ☐	Cotton Wool Spot	☐ ☐
	Other	☐ ☐	Other	☐ ☐
If other specify				

Han JED, *et al. BMJ Open* 2022; 12:e055845. doi: 10.1136/bmjopen-2021-055845

Moorfields Eye Hospital, Case Report Form
BALK1006

Moorfields Eye Hospital NHS Foundation Trust NHS

Study No: ☐☐ - ☐☐☐

HERMES Study

BASELINE VISIT (Community Optometry) **Page 2/2**

**ALL FIELDS ARE MANDATORY**				
**Baseline Exam**	**Right Eye**		**Left eye**	
Intraocular pressure		*(0-60)* ☐☐		*(0-60)* ☐☐
OCT taken?	Yes ☐	No ☐	Yes ☐	No ☐
OCT qualitative	SRF ☐<br>PED ☐	IRF ☐<br>SHRM ☐	SRF ☐<br>PED ☐	IRF ☐<br>SHRM ☐
Referral Recommendation by Optometrist (both arms)	Urgent ☐	Routine ☐	No referral ☐	
If not referred, specify reason				

Comments:

I have completed this form in full and take full responsibility for any missing data.

**Signed:**	**Print:**	**Date:**
		☐☐ / ☐☐ / ☐☐☐☐
**Office use only, data entry completed by:**		
**Signed:**	**Print:**	**Date:**
		☐☐ / ☐☐ / ☐☐☐☐

Han JED, *et al. BMJ Open* 2022; 12:e055845. doi: 10.1136/bmjopen-2021-055845

Moorfields Eye Hospital, Case Report Form
BALK1006

Moorfields Eye Hospital NHS Foundation Trust NHS

Study No: ☐☐ - ☐☐☐

HERMES Study

REFERRALS (Community Optometry) **Page 1/3**

**ALL FIELDS ARE MANDATORY**	
**Referral Details**	
Has referral been generated?	***(control arm)*** Yes ☐ No ☐
If yes please specify date	***(dd mm yyyy)*** ☐☐ / ☐☐ / ☐☐☐☐
Referral reference number	***(control arm)***
Referral system	***(control arm)*** Post ☐ Electronic ☐ Other ☐
If other, please specify	
Have OCT scans been uploaded on eCRF?	***(both arms)*** Yes ☐ No ☐
If no, specify reason	
Has clinical data been uploaded on eCRF?	***(intervention arm)*** Yes ☐ No ☐
If no, specify reason	
Optometrist referral recommendation accepted after HES triage?	***(both arms)*** Yes ☐ No ☐
If yes, eye on which referral decision was based	Right ☐ Left ☐ Both ☐
If no, referral decision after HES triage	Urgent ☐ Routine ☐ No referral ☐
Reason why recommendation was not accepted ***(field to be completed by HES)***	

Han JED, *et al. BMJ Open* 2022; 12:e055845. doi: 10.1136/bmjopen-2021-055845

Moorfields Eye Hospital, Case Report Form
BALK1006

Moorfields Eye Hospital NHS Foundation Trust NHS

Study No: ☐☐ - ☐☐☐

HERMES Study
REFERRALS (tele-HES) **Page 2/3**

**ALL FIELDS ARE MANDATORY**		
**Tele - HES Review**		
OCT from referral reviewed by tele-HES?	Yes ☐	No ☐
	**Right Eye**	**Left eye**
OCT from referral *qualitative by tele-HES*	SRF ☐ IRF ☐<br>PED ☐ SHRM ☐	SRF ☐ IRF ☐<br>PED ☐ SHRM ☐
Clinical findings	Yes No<br>Macular Haemorrhage ☐ ☐<br>Other Retinal Haemorrhage ☐ ☐<br>Exudates ☐ ☐<br>Disc Swelling ☐ ☐<br>Macular Atrophy ☐ ☐<br>Cotton Wool Spot ☐ ☐<br>Other ☐ ☐	Yes No<br>Macular Haemorrhage ☐ ☐<br>Other Retinal Haemorrhage ☐ ☐<br>Exudates ☐ ☐<br>Disc Swelling ☐ ☐<br>Macular Atrophy ☐ ☐<br>Cotton Wool Spot ☐ ☐<br>Other ☐ ☐
If other specify		
**Tele-HES Review**	**Right Eye**	**Left eye**
Diagnosis from referring optometrist confirmed by tele-HES	Yes ☐	No ☐
Diagnosis by tele-HES	Yes No<br>Wet AMD ☐ ☐<br>Dry AMD ☐ ☐<br>Central Serous Chorioretinopathy ☐ ☐<br>Central Retinal Vein Occlusion ☐ ☐<br>Branch Retinal Vein Occlusion ☐ ☐<br>Diabetic Macular Oedema ☐ ☐<br>Inherited Eye Disease ☐ ☐<br>Other ☐ ☐	Yes No<br>Wet AMD ☐ ☐<br>Dry AMD ☐ ☐<br>Central Serous Chorioretinopathy ☐ ☐<br>Central Retinal Vein Occlusion ☐ ☐<br>Branch Retinal Vein Occlusion ☐ ☐<br>Diabetic Macular Oedema ☐ ☐<br>Inherited Eye Disease ☐ ☐<br>Other ☐ ☐
If other specify		

Han JED, *et al. BMJ Open* 2022; 12:e055845. doi: 10.1136/bmjopen-2021-055845

Moorfields Eye Hospital, Case Report Form
BALK1006

Moorfields Eye Hospital NHS Foundation Trust NHS

Study No: ☐☐ - ☐☐☐

HERMES Study

REFERRALS (tele-HES) **Page 3/3**

ALL FIELDS ARE MANDATORY	
**Tele - HES Review**	
Referral recommendation by referring optometrist confirmed by tele-HES	Yes ☐ No ☐
If yes, eye on which referral decision was based	Right ☐ Left ☐ Both ☐
Referral Decision by tele-HES	Urgent ☐ Routine ☐ No referral ☐

Comments:

I have completed this form in full and take full responsibility for any missing data.

Signed:	Print:	Date:
		☐☐ / ☐☐ / ☐☐☐☐
**Office use only, data entry completed by:**		
**Signed:**	**Print:**	**Date:**
		☐☐ / ☐☐ / ☐☐☐☐

Han JED, *et al. BMJ Open* 2022; 12:e055845. doi: 10.1136/bmjopen-2021-055845

Moorfields Eye Hospital, Case Report Form
BALK1006

Moorfields Eye Hospital NHS Foundation Trust NHS

Study No: ☐☐ - ☐☐☐

HERMES Study

HES First Visit **Page 1/2**

ALL FIELDS ARE MANDATORY				
**HES Review**				
Follow up status	Attended ☐	Cancelled ☐	DNA ☐	
Date of consultation	(dd mm yyyy) ☐☐/☐☐/☐☐☐☐			
Change in eye medication	Yes ☐	No ☐		
If yes, specify				
Date of first treatment (if applicable)	(dd mm yyyy) ☐☐/☐☐/☐☐☐☐			
	**Right Eye**		**Left eye**	
Intraocular pressure	(0-60) ☐☐		(0-60) ☐☐	
OCT taken?	Yes ☐	No ☐	Yes ☐	No ☐
OCT qualitative	SRF ☐ PED ☐	IRF ☐ SHRM ☐	SRF ☐ PED ☐	IRF ☐ SHRM ☐
Visual acuity (ETDRS)	(ETDRS letters, range 0-95) ☐☐		(ETDRS letters, range 0-95) ☐☐	
Visual acuity (Snellen)	☐ / ☐☐		☐ / ☐☐	
Clinical findings by HES		Yes / No		Yes / No
	Macular Haemorrhage	☐ ☐	Macular Haemorrhage	☐ ☐
	Other Retinal Haemorrhage	☐ ☐	Other Retinal Haemorrhage	☐ ☐
	Exudates	☐ ☐	Exudates	☐ ☐
	Disc Swelling	☐ ☐	Disc Swelling	☐ ☐
	Macular Atrophy	☐ ☐	Macular Atrophy	☐ ☐
	Cotton Wool Spot	☐ ☐	Cotton Wool Spot	☐ ☐
	Other	☐ ☐	Other	☐ ☐
If other specify				
Diagnosis by HES		Yes / No		Yes / No
	Wet AMD	☐ ☐	Wet AMD	☐ ☐
	Dry AMD	☐ ☐	Dry AMD	☐ ☐
	Central Serous Chorioretinopathy	☐ ☐	Central Serous Chorioretinopathy	☐ ☐
	Central Retinal Vein Occlusion	☐ ☐	Central Retinal Vein Occlusion	☐ ☐
	Branch Retinal Vein Occlusion	☐ ☐	Branch Retinal Vein Occlusion	☐ ☐
	Diabetic Macular Oedema	☐ ☐	Diabetic Macular Oedema	☐ ☐
	Inherited Eye Disease	☐ ☐	Inherited Eye Disease	☐ ☐
	Other	☐ ☐	Other	☐ ☐
If other specify				

Han JED, et al. BMJ Open 2022; 12:e055845. doi: 10.1136/bmjopen-2021-055845

Moorfields Eye Hospital, Case Report Form
BALK1006

Moorfields Eye Hospital NHS Foundation Trust NHS

Study No: ☐☐ - ☐☐☐

HERMES Study

HES First Visit **Page 2/2**

ALL FIELDS ARE MANDATORY		
**HES Review**	**Right Eye**	**Left eye**
	Yes No	Yes No
Additional Diagnostic Procedures	OCT - Angio ☐ ☐<br>IGCA ☐ ☐<br>FA ☐ ☐<br>Optos ☐ ☐<br>Ultrasound B Scan ☐ ☐<br>Visual Field Test ☐ ☐<br>Other ☐ ☐	OCT - Angio ☐ ☐<br>IGCA ☐ ☐<br>FA ☐ ☐<br>Optos ☐ ☐<br>Ultrasound B Scan ☐ ☐<br>Visual Field Test ☐ ☐<br>Other ☐ ☐
If other specify		

Comments:

I have completed this form in full and take full responsibility for any missing data.

**Signed:**	**Print:**	**Date:**
		☐☐ / ☐☐ / ☐☐☐☐
**Office use only, data entry completed by:**		
**Signed:**	**Print:**	**Date:**
		☐☐ / ☐☐ / ☐☐☐☐

Han JED, *et al. BMJ Open* 2022; 12:e055845. doi: 10.1136/bmjopen-2021-055845

Moorfields Eye Hospital, Case Report Form
BALK1006

Moorfields Eye Hospital NHS Foundation Trust NHS

Study No: ☐☐ - ☐☐☐

HERMES Study

READING CENTRE **Page 1/2**

ALL FIELDS ARE MANDATORY		
**Reference Standard**	**Right Eye**	**Left eye**
Diagnosis by the reference standard	Yes / No<br>Wet AMD ☐ ☐<br>Dry AMD ☐ ☐<br>Central Serous Chorioretinopathy ☐ ☐<br>Central Retinal Vein Occlusion ☐ ☐<br>Branch Retinal Vein Occlusion ☐ ☐<br>Diabetic Macular Oedema ☐ ☐<br>Inherited Eye Disease ☐ ☐<br>Other ☐ ☐	Yes / No<br>Wet AMD ☐ ☐<br>Dry AMD ☐ ☐<br>Central Serous Chorioretinopathy ☐ ☐<br>Central Retinal Vein Occlusion ☐ ☐<br>Branch Retinal Vein Occlusion ☐ ☐<br>Diabetic Macular Oedema ☐ ☐<br>Inherited Eye Disease ☐ ☐<br>Other ☐ ☐
If other specify		
Referral decision by reference standard	Urgent ☐ Routine ☐	No referral ☐
**Artificial Intelligence Moorfields DeepMind**	**Right Eye**	**Left eye**
Deep-Mind Diagnosis of retinal disease	Yes / No<br>Wet AMD ☐ ☐<br>Dry AMD ☐ ☐<br>Central Serous Chorioretinopathy ☐ ☐<br>Central Retinal Vein Occlusion ☐ ☐<br>Branch Retinal Vein Occlusion ☐ ☐<br>Diabetic Macular Oedema ☐ ☐<br>Inherited Eye Disease ☐ ☐<br>Other ☐ ☐	Yes / No<br>Wet AMD ☐ ☐<br>Dry AMD ☐ ☐<br>Central Serous Chorioretinopathy ☐ ☐<br>Central Retinal Vein Occlusion ☐ ☐<br>Branch Retinal Vein Occlusion ☐ ☐<br>Diabetic Macular Oedema ☐ ☐<br>Inherited Eye Disease ☐ ☐<br>Other ☐ ☐
If other specify		
Referral decision by Moorfields DeepMind	Urgent ☐ Routine ☐	No referral ☐

Han JED, *et al. BMJ Open* 2022; 12:e055845. doi: 10.1136/bmjopen-2021-055845

Moorfields Eye Hospital, Case Report Form
BALK1006

Moorfields Eye Hospital NHS Foundation Trust NHS

Study No: ☐☐ - ☐☐☐

HERMES Study

READING CENTRE **Page 2/2**

ALL FIELDS ARE MANDATORY	
**Artificial Intelligence Moorfields-DeepMind**	
Time from receiving the OCT scans and a referral decision (hours)	*(hours)* ☐☐
End-to-end inference speed of technical infrastructure supporting the AI DSS	*(minutes)* ☐☐
Any technical issues	Yes ☐ No ☐
If yes, specify details	

Comments:

I have completed this form in full and take full responsibility for any missing data.

**Signed:**	**Print:**	**Date:**
		☐☐ / ☐☐ / ☐☐☐☐
**Office use only, data entry completed by:**		
**Signed:**	**Print:**	**Date:**
		☐☐ / ☐☐ / ☐☐☐☐

Study No: ☐☐-☐☐☐

HERMES Study

APPENDIX 1 – PROTOCOL DEVIATION

ALL FIELDS ARE MANDATORY	
**Deviation Details**	
Deviation date	*(dd mm yyyy)* ☐☐/☐☐/☐☐☐☐
Type of deviation	
Any outcomes or actions	
Deviation date	*(dd mm yyyy)* ☐☐/☐☐/☐☐☐☐
Type of deviation	
Any outcomes or actions	
Deviation date	*(dd mm yyyy)* ☐☐/☐☐/☐☐☐☐
Type of deviation	
Any outcomes or actions	
Deviation date	*(dd mm yyyy)* ☐☐/☐☐/☐☐☐☐
Type of deviation	
Any outcomes or actions	

I have completed this form in full and take full responsibility for any missing data.

**Signed:**	**Print:**	**Date:**
		☐☐/☐☐/☐☐☐☐
**Office use only, data entry completed by:**		
**Signed:**	**Print:**	**Date:**
		☐☐/☐☐/☐☐☐☐

Han JED, *et al. BMJ Open* 2022; 12:e055845. doi: 10.1136/bmjopen-2021-055845

Moorfields Eye Hospital, Case Report Form
BALK1006

Moorfields Eye Hospital NHS Foundation Trust NHS

Study No: ☐☐ - ☐☐☐

HERMES Study

APPENDIX 2 – EARLY STUDY WITHDRAWAL

ALL FIELDS ARE MANDATORY	
**Withdrawal Details**	
Did the patients discontinue the trial prematurely for reasons other than being referred back to hospital care?	Yes ☐ No ☐
Date of premature Study Discontinuation	*(dd mm yyyy)* ☐☐ / ☐☐ / ☐☐☐☐
Primary reason for discontinuation *(tick one box only)*	Patient withdraws consent ☐<br>*If known, state reason:*<br>Patient is non-compliant ☐<br>Patient is lost to follow up ☐<br>Investigator feels that it is in the patient's best interest due to adverse event ☐<br>*Related AE No:*<br>Other reason for discontinuation ☐<br>*If Other specify:*
Does the patient still agree to have their data collected and analysed as part of intent to treat analysis?	Yes ☐ No ☐

I have completed this form in full and take full responsibility for any missing data.

**Signed:**	**Print:**	**Date:**
		☐☐ / ☐☐ / ☐☐☐☐
**Office use only, data entry completed by:**		
**Signed:**	**Print:**	**Date:**
		☐☐ / ☐☐ / ☐☐☐☐

Han JED, *et al. BMJ Open* 2022; 12:e055845. doi: 10.1136/bmjopen-2021-055845

Appendix 6. Trial Documents and Subject Records

6.1 CRFs and Source Document Identification

We will establish a hub and spoke structure, where each community optometry practice liaises with its local hospital site (Moorfields Eye Hospital NHS Foundation Trust, Birmingham University Hospitals NHS Foundation Trust, Central Middlesex Hospital at London North West University Healthcare NHS Trust, and North West Anglia NHS Foundation Trust) for the day-to-day operation of the trial, through the site coordinator located at each site. 8-10 optometry practices will be located in the catchment area of Moorfields Eye Hospital NHS Foundation Trust (4-5 control and 4-5 intervention), 4-6 in the catchment area of North West London NHS Foundation Trust (Central Middlesex Hospital) (2-3 control and 2-3 intervention), 4-6 in the catchment area of North West Anglia NHS Foundation Trust (2-3 control and 2-3 intervention) and 4-6 in the catchment area of Birmingham University Hospitals NHS Foundation Trust (2-3 control and 2-3 intervention). All sites, including community optometry and hospital sites transfer data (OCT and clinical data) to Moorfields Reading Centre. The digital referral platform will be used in the 12 intervention optometry practices and the 4 HES; OCTs and clinical data from patients in the intervention optometry practices will be transferred to HES via a digital referral platform for remote review ('tele-HES') by local human experts.

Both the control arm and interventional arm will use the trial database to complete the eCRF and securely upload OCT scans. The interventional arm also transfers OCT's to the patient's hub hospital via a secure tele-ophthalmology platform. The scans and data will then be matched with the relevant trial data in the eCRF database.

For the AI Diagnostic Accuracy study, the pseudonymised OCT scans will be securely transmitted from the Moorfields Reading Centre to a secure Google Cloud Healthcare DICOM store over an encrypted connection, where it will be analysed by the DeepMind algorithm. Results from this analysis will be logged in the eCRF database. The study's use of cloud computing infrastructure adheres to January 2018 guidance from NHS Digital regarding cloud computing for health and social care. All data will be handled in accordance with the Data Protection Act 2018.

6.2 Confidentiality of Trial Documents and Subject Records

The eCRF will not bear the subject's name or other personal identifiable data. A trial number will be used for identification on the CRFs. A separate log file which links the study ID and the patient's details, screening log and recruitment information will be kept on a protected NHS computer at hub sites. The key log will be kept at the recruitment site and will not be shared with the Sponsor. It will be the responsibility of the chief investigator or delegated trial member to ensure the accuracy of all data recorded on the CRFs. CRFs will be completed and signed off by the Chief Investigator or delegated/authorised individual as outlined in the delegation log, the completed CRFs will be checked for accuracy and completion by the trial co-ordinator prior to data entry.

6.3 Procedures for validation and securing of electronic clinical data systems

The eCRF will be developed by the Moorfields Eye Hospital database development team. The front end will use a bespoke web application and the back end (data storage) will be hosted on Moorfields Eye Hospital Research Database SQL servers. All servers are backed up daily and with multiple restore points every day and backup copies exist in more than one All MEH clinical trial databases are part of the MEH disaster recovery strategy and have a 5 day Recovery Time Objective.

6.4 Data handling and record keeping

Data queries will be sent to trial co-ordinators for clarification and confirmation whenever picked up. After all data queries are resolved and all errors are corrected, the database will then be locked with the agreement of King's CTU statistician and data will be exported by the applications manager and sent to trial statistician for data analysis. Pre-existing mechanisms for data transfer between Moorfields Eye Hospital CRF and King's CTU will be utilised.

Appendix 7. References

1 Kortuem K, Fasler K, Charnley A, et al. Implementation of medical retina virtual clinics in a tertiary eye care referral centre. *British Journal of Ophthalmology* 2018;102(10):1391-1395.

2 Agresti A and Coull B. Approximate is Better than “Exact” for Interval Estimation of Binomial Proportions. *The American Statistician* 1998;52(2):119-126.

3 Day A, Burr J, Bunce C, et al. Randomised, single-masked non-inferiority trial of femtosecond laser-assisted versus manual phacoemulsification cataract surgery for adults with visually significant cataract: the FACT trial protocol. *BMJ Open* 2015;5(11):p.e010381.

4 Theodossiades J, Murdoch I, and Cousens S. Glaucoma case finding: a cluster-randomised intervention trial. *Eye* 2004;18(5):483-490.

5 De Fauw J, Ledsam J, Romera-Paredes B, et al. Clinically applicable deep learning for diagnosis and referral in retinal disease. *Nature Medicine* 2018;24(9):1342-1350.
