# Supplementary material for: Teleophthalmology-enabled and artificial intelligence-ready referral pathway for community optometry referrals of retinal disease (HERMES): a Cluster Randomised Superiority Trial with a linked Diagnostic Accuracy Study—HERMES study report 1—study protocol
Source: BMJ Open. 2022 Feb 1;12(2):e055845. doi: 10.1136/bmjopen-2021-055845 (PMC8808461; doi:10.1136/bmjopen-2021-055845)
Supplement: Author's manuscript [file bmjopen-2021-055845.draft_revisions.pdf]

BMJ Open is committed to open peer review. As part of this commitment we make the peer review history of every article we publish publicly available.

When an article is published we post the peer reviewers' comments and the authors' responses online. We also post the versions of the paper that were used during peer review. These are the versions that the peer review comments apply to.

The versions of the paper that follow are the versions that were submitted during the peer review process. They are not the versions of record or the final published versions. They should not be cited or distributed as the published version of this manuscript.

BMJ Open is an open access journal and the full, final, typeset and author-corrected version of record of the manuscript is available on our site with no access controls, subscription charges or pay-per-view fees (http://bmjopen.bmj.com).

If you have any questions on BMJ Open's open peer review process please email info.bmjopen@bmj.com

BMJ Open

Tele-opHthalmology-enablEd and ARtificial Intelligence-ready referral pathway for coMmunity optomEtry referralS of retinal disease (HERMES): A Cluster Randomised Superiority Trial with a linked Diagnostic Accuracy Study Protocol

Journal:	*BMJ Open*
Manuscript ID	bmjopen-2021-055845
Article Type:	Protocol
Date Submitted by the Author:	28-Jul-2021

Complete List of Authors:	Han, Ji Eun; University of Birmingham Institute of Inflammation and Ageing,<br>Liu, Xiaoxuan; University Hospitals Birmingham NHS Foundation Trust,<br>Bunce, Catey; Royal Marsden Hospital NHS Trust, RM CTU<br>Douiri, Abdel; King's College London, Primary Care And Public Health Sciences<br>Vale, Luke; Newcastle University, Health Economics Group, Institute of Health and Society<br>Blandford, Ann; University College London, UCLIC<br>Lawrenson, John; City University London, Dvision of Optometry and Visual Science<br>Hussain, Rima; Moorfields Eye Hospital NHS Foundation Trust, NIHR Biomedical Research Centre; UCL, Institute of Ophthalmology<br>Grimaldi, Gabriela; Moorfields Eye Hospital NHS Foundation Trust, NIHR Biomedical Research Centre; UCL, Institute of Ophthalmology<br>Learoyd, Annastazia; King's College London, School of Population Health & Environmental Sciences<br>Kernohan, Ashleigh; Newcastle University, Population Health Sciences Institute<br>Dinah, Christiana; London North West Healthcare NHS Trust, Ophthalmology<br>Minos, Evangelos; North West Anglia NHS Foundation Trust<br>Sim, Dawn; Moorfields Eye Hospital NHS Foundation Trust,<br>Aslam, Tariq; University of Manchester,<br>Patel, Praveen; Moorfields Eye Hospital NHS Foundation Trust, NIHR Biomedical Research Centre; UCL, Institute of Ophthalmology<br>Denniston, Alastair; University Hospitals Birmingham NHS Foundation Trust<br>Keane, Pearse; Moorfields Eye Hospital NHS Foundation Trust<br>Balaskas, Konstantinos; Moorfields Eye Hospital NHS Foundation Trust, ; University of Manchester , School of Biological Sciences

Keywords:	OPHTHALMOLOGY, Medical retina < OPHTHALMOLOGY, HEALTH SERVICES ADMINISTRATION & MANAGEMENT, HEALTH ECONOMICS, Telemedicine < BIOTECHNOLOGY & BIOINFORMATICS

SCHOLARONE™
Manuscripts

Tele-opHthalmology-enablEd and ARtificial Intelligence-ready referral pathway for coMmunity optomEtry referralS of retinal disease (HERMES): A Cluster Randomised Superiority Trial with a linked Diagnostic Accuracy Study Protocol

Authors
Ji Eun Diana Han, Xiaoxuan Liu, Catey Bunce, Abdel Douiri, Luke Vale, Ann Blandford, John G Lawrenson, Rima Hussain, Gabriela Grimaldi, Annastazia Learoyd, Ashleigh Kernohan, Christiana Dinah, Evangelos Minos, Dawn Sim, Tariq Aslam, Praveen J. Patel, Alastair K Denniston, Pearse A Keane, Konstantinos Balaskas

Affiliations
NIHR Biomedical Research Centre at Moorfields Eye Hospital NHS Foundation Trust and UCL Institute of Ophthalmology, London. (Pearse Keane, Praveen J. Patel, Dawn Sim, Rima Hussain, Gabriela Grimaldi)
University College London (Pearse Keane, Prof Ann Blandford, Gabriela Grimaldi)
King's College London (Drs Abdel Douiri, Annastazia Learoyd)
City, University of London (Prof John Lawrenson)
University of Newcastle (Prof Luke Vale, Ashleigh Kernohan)
Manchester University NHS Foundation Trust (Prof Tariq Aslam)
University Hospitals Birmingham (Prof Alastair Denniston, Ji Eun Diana Han, Xiaoxuan Liu)
Central Middlesex Hospital (Christiana Dinah)
North West Anglia NHS Foundation Trust (Evangelos Minos)
Royal Marsden NHS Foundation Trust (Dr Catey Bunce)

Corresponding Author:
Konstantinos Balaskas
Moorfields Eye Hospital NHS Foundation Trust
162 City Road
London
EC1V 2PD
k.balaskas@nhs.net

Keywords: Telemedicine, tele-ophthalmology, artificial intelligence, machine learning, ophthalmology, retinal disease, randomised controlled trial, diagnostic accuracy

For peer review only

ABSTRACT

Introduction

Recent years have witnessed an upsurge of demand in eye care services in the UK. With a large proportion of patients referred to Hospital Eye Services (HES) for diagnostics and disease management, the referral process results in unnecessary referrals from erroneous diagnoses, and delays in access to appropriate treatment. A potential solution is a tele-ophthalmology digital referral pathway linking community optometry and HES.

Methods and analysis

The HERMES study is a cluster randomised clinical trial (C-RCT) for evaluating the effectiveness of a tele-ophthalmology referral pathway between community optometry and HES for retinal diseases. Nested within HERMES is a diagnostic accuracy study, which assesses the accuracy of an artificial intelligence (AI) decision support system (DSS) for automated diagnosis and referral recommendation. A post-implementation, observational sub-study, a within-trial economic evaluation and discrete choice experiment will assess the feasibility of implementation of both digital technologies within a real-life setting. Patients with a suspicion of retinal disease, undergoing eye examination and optical coherence tomography (OCT) scans, will be recruited across 24 optometry practices in the UK. Optometry practices will be randomised to standard care or tele-ophthalmology. The primary outcome is the proportion of false positive referrals (unnecessary HES visits) in the current referral pathway compared to the tele-ophthalmology referral pathway. OCT scans will be interpreted by the AI DSS which provides a diagnosis and referral decision and the primary outcome for the AI diagnostic study is diagnostic accuracy of the referral decision made by the Moorfields-DeepMind AI system. Secondary outcomes relate to inappropriate referral rate, cost-effectiveness analyses and human-computer interaction analyses.

Ethics and dissemination

Ethical approval was obtained from the London - Bromley Research Ethics Committee (REC 20/LO/1299). Findings will be reported through academic journals in ophthalmology, health services research and HCI.

Trial registration number: ISRCTN18106677

For peer review only

STRENGTHS AND LIMITATIONS OF THIS STUDY

- The HERMES study aims to generate multi-centre evidence to evaluate the effectiveness and cost effectiveness of a tele-ophthalmology referral system, providing the potential to streamline the referral pathway between community optometry and HES for patients with retinal disease.
- Our study design involves five major eye hospitals and clusters of community optometrists. Such a shared care system has the potential to relieve overloaded outpatient activity and, by extension, allow HES to reallocate resources towards “more-specialist” ophthalmic care.
- A key strength of HERMES is its potential to provide evidence-based implementation strategies that will be necessary for the adoption of Artificial Intelligence Decision Support System by incorporating the community-based interventional study.
- Our study will involve a comprehensive economic evaluation, coupled with a Human-Computer Interaction analysis, which allows exploration of the opportunities and barriers for the adoption of the proposed digital technologies in real-world practice settings.
- This pathway is designed for the UK health setting and may not be immediately generalisable for world-wide health systems. However, interventions such as this are timely and relevant to the global trend towards modernisation of health care delivery.

For peer review only

BACKGROUND

Ophthalmology outpatient attendances account for 10% of all outpatient activity in the United Kingdom (UK), more than any other individual medical specialty.[1, 2] Modern ophthalmic practice in the UK is faced by the challenges of an ageing population, increasing prevalence of degenerative disease, and emergent treatments that are revolutionary but dependent on timely diagnosis. This represents a huge strain on diagnostic services and adversely impacts on timely access to care. Concurrently, there have been exponential increases in computing power and artificial intelligence, expansions in the strength and ubiquity of communications technologies, and developments in imaging capabilities, including in the community optometry setting.[3]

In the UK, primary care for ophthalmology is delivered by community-based optometry practices (High Street opticians). A large proportion of patients diagnosed with a suspicion of retinal disease, including common conditions such as neovascular ('wet') age-related macular degeneration are referred to Hospital Eye Services (HES) for diagnostics and disease management.[4, 5] The referral process results in unnecessary referrals (which can cause inconvenience and distress for patients), erroneous diagnoses, misclassification in terms of urgency, duplication of imaging tests and delays in access to treatment.

An increase of 30% in eye clinic attendances has been observed within the last 5 years throughout the UK.[6] Further increases are likely because of the increasing availability of imaging technology, and especially Optical Coherence Tomography (OCT), which is becoming ubiquitous in community optometry practices.[7] OCT is a non-invasive imaging modality that uses light to generate micrometre-resolution three-dimensional images of the retina and provides the best way to diagnose a number of common retinal pathologies including wet AMD.

This study focuses on two potentially complementary digital technologies that have the potential to revolutionise the interface between community optometrists and hospital-based eye clinics: the tele-ophthalmology platform and the Moorfields-DeepMind Artificial Intelligence Decision Support System (DSS). The technologies will be assessed through two complementary and linked quantitative studies:

1. Cluster Superiority Randomised Trial (RCT) of tele-ophthalmology
2. Prospective diagnostic accuracy study of Artificial Intelligence (Machine Learning) Diagnosis Support (the Moorfields-DeepMind algorithm)

1. Cluster Superiority Randomised Trial (RCT) of Tele-ophthalmology Pathway

Tele-medicine in Ophthalmology can help face this challenge of provision of optimal and expert care to people attending for routine eye tests in community optometry practices, through a digital referral pathway relying on a tele-ophthalmology link between community optometry and HES. This could optimise the referral process by allowing remote review of imaging and clinical data captured at the community level, by human experts based in HES. We will perform a cluster randomised superiority trial to assess the impact on service delivery metrics (such as proportion of unnecessary referrals and time from referral to treatment for urgent maculopathies) of a digital link between community

For peer review only

optometry practices and HES using a tele-ophthalmology platform. We will use a device-agnostic, tele-ophthalmology platform to enable a digital referral pathway of patients with a suspicion of retinal disease. The pilot data produced by our research team has demonstrated the potential of tele-ophthalmology to drastically improve the efficiency of the referral pathway between community optometry and HES while reducing unnecessary referrals to HES[8] and hence we propose a randomised trial powered to demonstrate superiority of the digital referral pathway against standard care.

2. Diagnostic Accuracy Study of Artificial Intelligence Diagnosis Support System

Artificial Intelligence Decision Support Systems (AI DSS) have recently been developed and shown to have good diagnostic accuracy against human experts in interpreting ocular imaging tests, such as OCT scan.[9] The collaboration between Moorfields Eye Hospital and Google DeepMind produced the arguably most advanced Deep Learning decision support system in Ophthalmology, capable of interpreting OCT scans, providing diagnosis for retinal disease and suggesting urgency of referral. In silico analysis using retrospectively collected data has validated the tool against human experts for the diagnosis of retinal disease and referral recommendations and it has been shown to be non-inferior.[9] The algorithm is uniquely and independently used in Moorfields Eye Hospital for research purposes. Thus, while such work has demonstrated promising results, a prospective study is required to demonstrate its value in practice.

We will perform a prospective study to assess diagnostic (referral) accuracy of the Moorfields-DeepMind AI DSS when applied on the OCT scans collected in the context of the tele-ophthalmology RCT. This will allow maximum utilisation of collected data from the trial and will provide estimates and confidence intervals of diagnostic (referral) accuracy. All cases included in the RCT will be reviewed by the Moorfields-DeepMind AI DSS within 48 hours of obtaining the OCT scans and a referral decision (refer routinely, refer urgently, don't refer) will be made by the algorithm for each case and recorded. The referral decisions made by the AI DSS will not be implemented in practice, yet data of the time required to obtain these decisions and any technical issues encountered with its use will be captured. These data will be incorporated into the implementation science models, including Human-Computer Interaction analysis, value-based economic evaluation, and discrete choice experiment, to identify the potential opportunities and gaps in advancing the adoption of the Moorfields-DeepMind AI DSS.

3. Impact of the Covid-19 pandemic: Post-implementation Observational Pragmatic Sub-Study

NHS services underwent rapid and significant adjustments across the board in response to extreme challenges presented by the Covid-19 pandemic. Changes to healthcare services driven by necessity are not always underpinned by a robust evidence base for efficiency and safety. In Ophthalmology, as a response to the need for social distancing and minimising unnecessary hospital visits, tele-ophthalmology pathways were commissioned recently in some areas of England using digital link to facilitate referrals between community optometry and Hospital Eye Services. Greater Manchester was an early adopter of this approach and a majority of the optometry practices in that area are now referring to NHS eye units via a tele-ophthalmology link. This local change in standard care provides a

unique opportunity to examine whether tele-ophthalmology works under usual conditions within the NHS. This sub-study will allow us to record and measure variation in quality of health care within a local region to inform our inferences from the RCT on how the tele-ophthalmology pathway will perform within a real-life setting. We will thus perform a pragmatic, observational, post-implementation study involving community optometry practices in the Greater Manchester area. This will also serve as a safety analysis allowing us to identify potential safety signals of the tele-ophthalmology pathways and adding granularity to the economic and qualitative evaluations of the RCT.

Trial aims and objectives

There are two complementary aims (aims 1&2) pertaining to the linked quantitative studies assessing the two digital technologies ('tele-ophthalmology' and the 'Moorfields-DeepMind' AI). The qualitative research element (aim 3) using Human-Computer Interaction (HCI) methodology will run across both studies to provide evidence on implementation **(Box 1)**.

For peer review only

Box 1. Aims and objectives of the HERMES study

Aims	Objectives
1. To assess the effectiveness and efficiency of a digital referral pathway between community optometry and Hospital Eye Services for referral of retinal disease enabled by a device-agnostic, tele-ophthalmology platform (superiority C-RCT).	1. Primary Objective: To compare the proportion of referrals classified as unnecessary (cases that can be safely managed without a HES consultation) between current standard care and tele-ophthalmology digital referral pathway.<br>2. Secondary Objectives:<br>● To estimate the relative efficiency of the tele-ophthalmology digital pathway compared with standard care in both within trial-based evaluation.<br>● To compare the rate of inappropriate referrals (defined as wrong diagnosis or wrong level of urgency) between standard care and the tele-ophthalmology digital pathway.<br>● To capture the number of uncommon/complex retinal referrals to secondary care and the proportion that can be safely triaged through the tele-ophthalmology platform.<br>● To compare time from referral to review and/or treatment in HES for urgent referrals (such as Wet AMD and Retinal Vein Occlusions) between standard care and tele-ophthalmology digital pathway.<br>● To assess the number of false negatives (number of patients that would have benefited from a HES consultation but were deemed suitable for continued care in the community) (Safety assessment)
2. To estimate the diagnostic (referral) accuracy and assess the 'real-life' performance of an Artificial Intelligence Decision Support System (the Moorfields-DeepMind AI) in the context of referral pathways between community optometry and HES.	1. To estimate the diagnostic (referral) accuracy of the Moorfields-DeepMind AI for recommending referral to HES from community optometry practices.<br>2. To estimate the diagnostic accuracy of the Moorfields-DeepMind AI for the diagnosis of retinal disease.<br>3. To assess the cost-effectiveness of the introduction of the DeepMind algorithm in the referral pathway between community optometry and HES.<br>4. To assess the technical feasibility of utilising the Moorfields-DeepMind AI for real-time analysis of retinal OCT scan images.<br>5. To assess real-time operational performance of the Moorfields-DeepMind AI in the tele-ophthalmology referral pathway.
3. To assess patient and healthcare professional acceptability as well as the barriers and enablers for the adoption of these digital technologies in the context of referral pathways between community optometry and HES through a Human-	1. To understand current workflows and practices of staff and patients in community optometry and HES so as to identify key user requirements for tele-ophthalmology tools from the perspectives of both practitioners and patients (working with care settings with diverse established practices).<br>2. To oversee the deployment of a digital referral platform at selected participating sites to ensure acceptability and acceptance by all user groups, and to understand the adoption process.<br>3. To identify factors that shape professionals' and patients' attitudes to, and trust in, the Moorfields-DeepMind AI, and how

Computer Interaction approach.	to present information in ways that instil appropriate confidence.<br>4. To observe workflows and practices of staff and patients in community optometry practices and HES with already established tele-ophthalmology pathways, aiding identification of technical, logistical and human factors affecting implementation of tele-ophthalmology in real-life (pragmatic sub-study).
4. To estimate the effectiveness and efficiency of a digital referral pathway between community optometry (High Street Opticians) and the Hospital Eyes Services for referral of retinal diseases enabled by a tele-ophthalmology platform in a real-life, observational post-implementation sub-study.	1. To compare the proportion of referrals classified as unnecessary (cases that can be safely managed without a HES consultation) against Reference Standard and the intervention arm of the RCT.<br>2. To compare the rate of inappropriate referrals (defined as wrong diagnosis or wrong level of urgency) against the Reference Standard and the intervention arm of the RCT.<br>3. To assess the number of false negatives (number of patients that would have benefitted from a HES consultation but were deemed suitable to continued care in the community) (Safety assessment).<br>4. To compare time from referral to review and/or treatment in HES for urgent referrals (such as Wet AMD and Retinal Vein Occlusions) between post-implementation real-life tele-ophthalmology digital pathway and the intervention arm of the RCT.<br>5. To estimate the relative efficiency of the real-life tele-ophthalmology digital pathway compared with the RCT tele-ophthalmology pathway.

For peer review only

STUDY DESIGN AND PATIENT POPULATION

Study Design

Superiority C-RCT of Tele-Ophthalmology Pathway

An interventional superiority cluster randomised trial (RCT) will be performed comparing standard practice for referral of suspicious retinal disease with tele-ophthalmology between community optometry and HES. This part of the study will be reported according to the CONSORT extension for C-RCTs.[10]

Diagnostic Accuracy Study of Artificial Intelligence Diagnosis Support System

A prospective study will be conducted using the data of the above RCT to assess the diagnostic (referral) accuracy of an advanced AI DSS (the Moorfields-DeepMind Algorithm) for the automated diagnosis and referral recommendation for retinal disease. OCT scans transferred to the Moorfields Reading Centre in the course of the study will be assessed by the DeepMind algorithm in 'real-time' and its referral recommendations will be recorded and analysed for diagnostic (referral) accuracy and compared against the performance of human experts in the standard care and tele-ophthalmology arms of the RCT. This part of the study will be reported according to the STARD 2015 statement,[11] or STARD-AI.[12] A within-trial based economic evaluation will estimate the efficiency of alternative referral models for retinal disease. A Human-Computer Interaction (HCI) analysis using qualitative methods will assess feasibility of implementation of both digital technologies.

Observational, post-implementations, pragmatic sub-study

Community Optometry practices in the Greater Manchester area will continue to refer patients with suspicious retinal disease to HES using the locally established tele-ophthalmology digital pathway. Referral recommendations will be compared against a reference standard provided by Moorfields Reading Centre to inform the assessment of real-life effectiveness and efficiency of the tele-ophthalmology referral pathway.

Setting

For the C-RCT and diagnostic accuracy study, patients will be recruited at 24 optometry practices (clusters) in the catchment areas of 4 HES sites: Moorfields Eye Hospital NHS Foundation Trust (8-10 practices), Birmingham University Hospitals NHS Foundation Trust (4-6 practices), Central Middlesex Hospital at London North West University Healthcare NHS Trust (4-6 practices) and North West Anglia NHS Foundation Trust (4-6 practices). 12 clusters (each cluster is an optometry practice) will be randomised to standard care and 12 clusters to the intervention (tele-ophthalmology). This selection of sites includes urban, sub-urban and rural locations within the UK allowing the inferences made from this study to be applicable to more of the UK population. 2 additional optometry practices (clusters) will be randomised (1:1) in a reserve capacity in case of a cluster drop-out or in order to accelerate the recruitment process. (**Appendix 1**)

For peer review only

For the pragmatic, observational, post-implementation sub-study, patients will be recruited at 12 optometry practices (clusters) in the catchment area of Manchester University NHS Foundation Trust. These practices have adopted a tele-ophthalmology referral pathway as standard practices.

Eligible practices need to have OCT devices and the activity volume and track record of referral to HES that will allow achieving the per practice recruitment target.

All OCT scans and clinical vignettes from each case will be transferred to the Moorfields Reading Centre that will provide the reference standard (diagnosis and referral recommendation). All suitable OCT scans will be processed by the DeepMind algorithm at the Moorfields Reading Centre in 'real-time' for the AI diagnostic accuracy study.

Participants

Adults (≥18 years) attending for an eye examination at the participating community optometry practices who undergo an OCT scan will be considered for participation in the study. Only people with a suspicion of retinal disease in the opinion of the community optometrist will be recruited in the RCT and diagnostic accuracy study. As entire optometry practices (clusters) will be randomised into standard care or tele-ophthalmology, patients who are approached and agree to take part in the study will consent **(Appendix 2)** to data collection and analysis - there will not be patient-level randomisation. The patient-level inclusion criteria are outlined in **Box 2.**

Box 2. Patient-level selection criteria

Inclusion Criteria

- Able to give consent and understand the study
- Able to cooperate by following study specific instructions
- Adults (≥18 years) attending the involved community optometry practices who underwent an OCT scan
- Individuals who at the opinion of the community optometrist have any suspicion of a retinal condition (including atrophic ("dry") AMD, wet AMD, diabetic retinopathy, macular oedema, macular holes, epiretinal membranes, central serous chorio-retinopathy, genetic eye disease)
- Macular OCT scan performed at community optometry

Exclusion Criteria

- Individuals with any non-retinal ocular co-morbidities in either eye other than cataract
- Individuals with media opacities, inability to position or fixate or any other reason that prevents acquisition of good quality OCT scans (at the discretion of the community optometrist)

For peer review only

Allocation to trial groups

Simple randomisation will be performed for involved optometry practices into the intervention and control arms. Randomisation will be performed with the unit of allocation being the cluster rather than the individual and allocation concealment will be at the cluster level. Optometry practices will be randomised 1:1 to standard care or tele-ophthalmology stratified by the hospital site. Optometry practices are committed to the allocated study arm for the duration of the recruitment period or until they have recruited the minimum of the per cluster recruitment range (10 patients).

Superiority C-RCT of Tele-Ophthalmology Pathway

Community optometry practices will be randomised to either continue with standard care for referral of retinal disease to hospital-based eye clinics or move to the tele-ophthalmology digital referral pathway (Figure 1).

Intervention arm for Superiority C-RCT: Digital Pathway The intervention pathway is the tele-ophthalmology model for referral of patients with suspicion of retinal disease from community optometry to HES using a digital referral platform. Patients who attend at participating community optometry practices will undergo a clinical assessment and OCT scan.

Figure 1. Superiority cluster randomised trial arms.

Participants' OCT scan and clinical information will be transferred via the digital referral platform to corresponding HES. In each case, human expert clinicians based in HES (ophthalmologists or specialist optometrists with a minimum of two years' experience of independent practice in the context of HES retinal clinics) will make a referral decision remotely ('tele-HES') after review of OCT scan and clinical information on the digital referral platform. A referral recommendation by the community optometrist will also be recorded but not acted upon, to measure the proportion of correct/incorrect referrals in each arm. **(Appendix 3)**.

The remote review of OCT scans and clinical data at 'tele-HES' will be performed by expert clinicians (medics or specialist optometrists) experienced in retinal clinics (minimum of two years' experience of independent practice in the context of retinal clinics in HES) based at Moorfields Eye Hospital, Central Middlesex Hospital, North West Anglia NHS Foundation Trust Hospitals or Queen Elizabeth Hospital Birmingham with access to senior advice by Consultant Ophthalmologists specialising in retinal disease.

Control arm for Superiority C-RCT: Standard Practice

The control pathway is standard practice for referral of patients with suspicion of retinal disease from community optometry to HES. Patients who attend a participating community optometry practice will undergo a clinical assessment and OCT scan. Patients with a suspicion of any retinal disease in the opinion of the community optometrist will be included in the study and will receive a referral decision (refer urgently to HES; refer routinely to HES; don't refer to HES) by the community optometrist. All OCT scans and a clinical vignette from each case will be transferred to the Moorfields Reading Centre that will provide the reference standard for referral recommendations.

For peer review only

Diagnostic Accuracy Study

All referred and non-referred cases (from the standard care and tele-ophthalmology arm above; **Figure 2**) will be included in the AI diagnostic study. All suitable OCT scans will be transferred prospectively on a weekly basis to the Moorfields Ophthalmic Reading Centre. OCT scans will be processed by the Moorfields-DeepMind AI system and end-to-end timing of the process will be captured for each case. For each case the Moorfields-DeepMind AI will provide a:

1. Diagnosis
2. Decision to refer or not
3. Urgency of referral (routine or urgent)

Figure 2. Diagnostic accuracy study arms.

Safety Net

In cases where 'tele-HES' decision is 'don't refer', the patient will be provided with additional information and alerts for clinical symptoms that should prompt a visit directly to the A&E department of the corresponding secondary care site (Moorfields Eye Hospital, Central Middlesex Hospital, North West Anglia NHS Foundation Trust Hospitals or Queen Elizabeth Hospital Birmingham). Additionally, in cases where a disagreement is found between the decision by the community optometrist and the one made in 'tele-HES', patients will be offered a follow-up appointment at the community optometry practice within 4 weeks.

Reference Standard

OCT scans from the standard care and tele-ophthalmology arms will be transferred to the Moorfields Ophthalmic Reading Centre. The reference standard will be provided by the expert Ophthalmic Reading Centre for the C-RCT, diagnostic accuracy study, and the Pragmatic sub-study. The reference standard will be the referral decisions and disease diagnosis made at the Reading Centre on the basis of review of images and clinical history and will apply to the RCT, the AI Diagnostic Accuracy study and the Post-implementation, Pragmatic sub-study. Specifically, for each patient, the OCT scan (including b-scans and colour fundus image) and a clinical vignette including visual acuity, age, symptoms, ocular and systemic history will be reviewed by two expert graders. The process to be followed is double-grading with adjudication by a senior retinal specialist at the Moorfields Reading Centre.

Outcome Measures

The outcomes for the superiority C-RCT and diagnostic accuracy study are outlined in **Box 3.**

Box 3. Study outcomes

**Superiority C-RCT**	**Diagnostic Accuracy Study**	**Pragmatic Sub Study**

For peer review only

Primary Outcome C-RCT: Proportion of false positive referrals in the current referral pathway and the tele-ophthalmology referral pathway.	Primary outcome diagnostic accuracy study: Diagnostic accuracy of the referral decision made by the Moorfields-DeepMind AI (refer to HES, do not refer to HES) against the Reference Standard (Moorfields Reading Centre).	
Secondary outcomes C-RCT:<br>1. Proportion of wrong diagnosis and wrong referral urgency in standard and tele-ophthalmology pathways against the reference standard<br>2. Proportion of false negative referrals, as well as sensitivity and specificity in standard and tele-ophthalmology pathways against the reference standard<br>3. Time from referral to consultation for urgent and routine referrals in standard and tele-ophthalmology pathways<br>4. Time from referral to treatment for urgent maculopathies in standard and tele-ophthalmology pathways<br>5. Number of uncommon referrals (rare disease) that can be safely triaged in the tele-ophthalmology pathway<br>6. Within trial cost-effectiveness and cost-consequences of the tele-ophthalmology digital pathway compared with standard care<br>7. Modelled cost-consequences and net benefits of alternative	Secondary outcomes diagnostic accuracy study:<br>1. Sensitivity and specificity of Moorfields-DeepMind AI for the diagnosis of retinal disease<br>2. Sensitivity and specificity of Moorfields-DeepMind AI for referral urgency<br>3. Proportion of false positive referrals in the standard and tele-ophthalmology pathways when human assessors are replaced by the AI DSS<br>4. Proportion of wrong diagnosis and wrong referral urgency in the standard and tele-ophthalmology pathways when human assessors are replaced by AI DSS<br>5. Uptime and end-to-end inference speed of technical infrastructure supporting the AI DSS<br>6. Average time of end-to-end output (referral recommendation) by the AI DSS<br>7. Cost-consequences and net benefits of AI-enabled digital referral pathway	Secondary outcomes of pragmatic sub study:<br>1. Proportion of false positive referrals in the tele-ophthalmology referral pathway against the Reference Standard and the intervention arm in the main RCT.<br>2. Proportion of wrong diagnosis and wrong referral urgency in the tele-ophthalmology pathway compared against the Reference Standard and the intervention arm in the main RCT study<br>3. Proportion of false negative referrals compared against the Reference Standard and the intervention arm in the main RCT study<br>4. Time from referral to review and/or treatment in HES for urgent referral) in the post-implementation real-life tele-ophthalmology digital pathway

diagnostic and referral strategies		

Patient and Public Involvement

Patient and public members were consulted prior to the trial protocol design on perception of tele-ophthalmology and issues of data privacy, impersonal care, trust in the technology, confidence in the quality of care provided through digital means were explored. A patient and public involvement group based at Moorfields Eye Hospital was consulted during the trial design to advise on barriers to recruitment, issues around geographical spread of study sites and patient information material. After study commencement, the PPI group is planned to meet yearly and an end-of-study debrief is planned with all PPI contributors, which will include discussions of the prioritisation and dissemination of study results both to the public as well as relevant healthcare professionals.

For peer review only

DATA ANALYSIS PLAN

Statistical Analysis

In the Superiority C-RCT of tele-ophthalmology pathway study, the primary analysis will be conducted following an intention-to-treat principle where all randomised patients are analysed in their allocated group whether or not they receive their randomised management plan. The primary outcome is the proportion of false referrals, measured at the patient level. This will be compared between management groups using logistic regression adjusting for clustered centres. Outcomes will be reported as adjusted odds ratios.

In the AI diagnostic accuracy study, we will report estimates of sensitivity and specificity of the DeepMind algorithm for referral decisions with 95% confidence intervals. Our primary analysis will combine urgent and standard referral to HES and compare against no referral to HES but a sensitivity analysis will be conducted to evaluate urgent referrals. The full statistical consideration and analysis is provided in **Appendix 4.**

Implementation Science Study Components

Focus of Analysis

The primary focus for analysis will be on facilitators and barriers to implementation of the tele-ophthalmology system and the introduction of Artificial Intelligence Decision Support across clinical contexts, along with accounts of how it changes workflow and patient experience. Evaluation will be formative, so as to inform future implementations and also to contextualise the analyses of clinical effectiveness and cost effectiveness. For the AI DSS, questions to be included in interviews will involve whether the AI is to be used as decision aid (e.g., as a filter for disease/no disease) or as a completely independent decision making tool, issues around trust in the technology, perceptions of medicolegal concerns (who is responsible for the decisions?), the optimal place in the care pathway for positioning the AI (high street optician or hospital-based eye services or both), concerns such as de-skilling of practitioners (as diagnostic decisions may be devolved to AI), reduced employment opportunities, the need for a 'safety net'/quality check to oversee and 'sanity check' the performance of the AI system, and impersonal care for patients, and perceived benefits such as more efficient and appropriate care, greater confidence in the process, etc. We will also particularly focus on the question of ‘interpretability’ of AI DSS and the ‘black box’ phenomenon and whether it influences trust and potential uptake of this technology. The ‘interpretability’ of AI DSS is a major factor in technology uptake and may influence the direction of AI DSS developers towards more interpretable technologies.

The data collected from sites with established tele-ophthalmology pathways (Greater Manchester) will be particularly valuable for identifying barriers to implementation in a real-life context that wouldn’t be picked up in the controlled environment of the RCT such as technical, staffing, training and human factors. Such barriers can be consequential with respect to patient safety as they have the potential to lead to delays in clinical review or missed cases and therefore the post-implementation sub-study

For peer review only

offers an opportunity to explore potential safety signals of the tele-ophthalmology pathways not typically observable in the context of RCT trials.

Human-Computer Interaction (HCI) Analysis

As noted above, the aims of the HCI analyses are to assess the barriers and enablers for the adoption of the proposed digital technologies in the context of referral pathways between community optometry and HES through a Human-Computer Interaction approach.

In order to capture patient and staff perspectives of tele-ophthalmology models of care as well as AI DSS, we will take a qualitative approach, conducting interviews and observations in both community optometry and HES. A full account on sampling, recruitment and analysis methods is provided in **Appendix 1**. We will compare people's expectations (what they believe they will want and use) with their experiences when they have access to the relevant technology. In order to compare expectations against experiences, we will gather data in a variety of settings over the course of the project:

- *In the first six months* of the project, the focus will be on understanding the adoption process and factors that contribute to success in adoption. Longitudinal data will be gathered at three selected sites: two optometry practices and the Birmingham HES. Data gathering will focus on expectations and current work practices before implementation; barriers, facilitators and experiences during implementation; and perceptions and practices post-implementation.
- *Over the subsequent 12 months* (months 7-18), similar data gathering and analysis methods will be adopted in two optometry practices that are already experienced in using tele-ophthalmology (sites that have already adopted tele-ophthalmology in the Greater Manchester area); two practices that are not using tele-ophthalmology and have no immediate plans to transition (control sites for the quantitative studies described above); and a second HES (Moorfields Eye Hospital).The focus will be on understanding workflows, practices and user requirements, including facilitators and barriers to adoption; and identifying factors that shape attitudes to the AI DSS, and how to present information to instill appropriate confidence.

Economic Analysis

Cost-Consequence Analysis

The economic evaluation will comprise a within trial cost-consequence analysis (CCA) directly comparing the tele-ophthalmology pathway with the current referral pathway. This analysis highlights the choices and trade-offs between the modalities of care provision without an explicit synthesis of data into a single measure of efficiency. The results for the cost-consequence analysis will be presented as a balance sheet, which will include point estimates and appropriate measures of variance. From an NHS perspective, costs such as hospital visits, medications and community GP visits will be costed. Unit costs for resource use will be derived from published sources e.g. NHS Reference Costs and Unit Costs of Health and Social Care.[13] When considering the addition of the community optometry

For peer review only

perspective, the costs to purchase and maintain an OCT scanner will be considered. The costs of acquisition will be derived from market prices and converted into a cost per patient using standard economic methodology.[14] In addition to this, the costs of the Moorfields-DeepMind algorithm will be considered within trial analysis. We will base this cost on advice from the algorithm owners as well as consideration of analogous algorithms. We expect there to be considerable uncertainty around the price to the NHS as a market price is not available. Therefore, we will explore the impact on efficiency of a range of prices. This will help decision-makers consider the maximum price they might be willing to pay for this algorithm given the benefits it may provide. In addition to the costs of running the algorithm in terms of hardware, software and staff, required will be considered. This will be based upon its use within the study and advice from members of the study. A sensitivity analysis will be carried out to explore how the adoption of different perspectives (i.e. who is bearing the costs) will affect the cost effectiveness of the intervention. Outcomes which may be included in this CCA are; false positives, false negatives, unnecessary hospital visits and duration of the time spent with an untreated macular disorder. These will be compared to the costs of provision and of the intervention and with the results of the Discrete Choice Experiment (DCE) described below. Costs that will be included will be those that fall on the NHS and community optometry practices. Deterministic sensitivity analysis e.g. variations in unit costs, will also be conducted. The consequences for each of the comparators will be based upon a further consideration of outcomes (for example necessary referrals missed, correct referrals, individuals correctly not referred). The likelihood of these different outcomes (given as percentages) will be described.

To include insights derived from the pragmatic post-implementation study, the economic evaluation will also provide the following additional elements:

1. The Manchester sub-study group will inform estimates of the cost of the intervention, as delivered in a ‘real world’ application which may be more realistic than those estimated from a trial setting.
2. The Manchester sub-study group will be used to inform an exploratory analysis. In this the costs and consequences of the real life sub-study group will be compared to the results from the trial group to identify if there is any meaningful difference between the two sets of data and identify what the driving factors are. As a safety analysis will be carried out as part of a sub-study, the cost and consequences of any unexpected adverse events that are recorded will be included in the cost consequence analysis. If any safety events become apparent during the design of the Discrete Choice Experiment then these may be used as the basis of different attributes and levels in the study design.

Discrete Choice Experiment (DCE)

In addition to the CCA economic evaluation a Discrete Choice Experiment (DCE) will be carried out to assess preferences of the general public about the tele-ophthalmology pathway. A DCE is an attribute-based survey method for measuring benefits. It offers participants at least two alternative choices which vary across several attributes of interest. These can include several attributes of how the intervention is provided and its effect on health and other outcomes. Each of these attributes can vary over a range

For peer review only

of levels. The choice of DCE attributes for this intervention will be informed from existing literature on macular disease and provision of eye care service. The output of the qualitative study will also be examined for any attributes which could affect the preferences of the users of the service. The DCE will also be used to value in monetary terms the relative importance of the different consequences included in the CCA. To do this it will use methods previously successfully used in other NIHR funded studies.[15, 16] The results of this monetary value will be used to inform a further cost benefit analysis.

Cost Benefit Analysis

The results of the DCE will be used to value the outcomes described in the cost-consequence analysis. Outcomes will be expressed as a net monetary benefit by combining the differences in each outcome by the willingness to pay for a unit change in that outcome. These values are known as willingness to pay (WTP) values and will be derived from the DCE described above. Cost will be included as part of the attributes that participants choose between so that participants can express their WTP values for a described outcome. The cost of the providing the outcome will be derived from the unit costs described earlier. A net benefit in monetary terms for these outcomes will be derived be subtracting the cost of the outcome from the WTP value (including a negative net benefit).

The values for the cost attribute will be based on pilot work and reviews of prior studies in this area for example Burr et al. (2012) valued an intervention to monitor ocular hypertension to prevent glaucoma using a DCE. The range of values was between £15-70 (GBP 2012). Similarly, Shih et al. (2007) assessed the WTP for a diabetic retinopathy screening service and reported a narrower range of between $4-$24 (USD 2007).[17] After the attributes have been established then the piloting stages will occur. A survey company will be utilised to gain a large enough sample (www.researchnow.co.uk). The participants will be offered a small incentive (£1-£2) to complete the survey. The overall sample will be representative as closely as possibly for factors such as age, sex and ethnic background for the UK population. Optimal sample size requirements for the limited dependent variable models estimated in DCEs depend on knowledge of the true choice probabilities, which are not known prior to undertaking this research. However, previous DCE studies have shown that robust choice models can be estimated from sample sizes between 50-100 respondents. As such, a small pilot sample of 100 participants will be used as a sample to monitor the rate of completion and to carry out preliminary analysis and change any parts of the survey that are necessary. After the preliminary analysis is carried out, then a further sample of 300 participants will be surveyed which will be sample size comparable to other HTAs in this area. The results of the DCE will be analysed using conditional logit regression analysis, which will measure the direction and strength of the participant preferences. Sub-group analysis will also be carried out to see if factors such as age, sex or ethnic background have any effect on the resulting preferences. Probabilistic and deterministic sensitivity analyses will be carried out to vary parameter uncertainty for both the costs and the effects. Results of the probabilistic sensitivity analysis will be presented as point estimates of net-benefits, plots of costs and benefits net benefit curves, which show the likelihood of each intervention being most likely to have the highest net benefit.

For peer review only

Dissemination of Results

Findings will be reported through academic journals in ophthalmology, health services research and HCI; some will focus on findings from the HCI studies, and some will relate findings to those of the parallel studies covering other themes. PPI workshops will be organised in Birmingham and London before the main study period, with a focus on designing the study adapting the model from an earlier project (the “before” study is reported by Furniss et al., 2016).[18]

ETHICAL CONSIDERATIONS

The research project will adhere to the UK Framework for Health and Social Care research. Ethics approval has been obtained for this project. No particular challenges are expected given the low risk nature of the intervention of the RCT, the safety net arrangements for cases not referred to HES from community optometry, the observational design of the AI diagnostic accuracy study, and the relatively low personal sensitivity of the topics to be investigated in the HCI studies.

Patients lacking capacity or unable to understand the study will not be eligible. A record will be kept at each optometry site that captures reasons for patients not recruited on the study. As recruitment will take place at community optometry practices, translator services will not be provided.

FUNDING

The project is funded by an NIHR Health Technology Assessment grant (HTA - Project: 18/182 HTA Digital Technologies to Improve Health and Care).

For peer review only

REFERENCES

1 The Royal College of Ophthalmologists, 2021. *The Way Forward*. [online] Rcophth.ac.uk. Available at: <https://www.rcophth.ac.uk/wp-content/uploads/2015/10/RCOphth-The-Way-Forward-Executive-Summary-300117.pdf> [Accessed 9 June 2021].

2 NHS Digital. 2020. *Hospital Outpatient Activity 2019-20: Main Specialty*. [online] Available at: <https://digital.nhs.uk/data-and-information/publications/statistical/hospital-outpatient-activity/2019-20> [Accessed 9 June 2021].

3 Dabasia P, Edgar D, Garway-Heath D, et al. A survey of current and anticipated use of standard and specialist equipment by UK optometrists. *Ophthalmic and Physiological Optics 2014*;34(5):592-613.

4 Looker H, Nyangoma S, Cromie D, et al. Rates of referable eye disease in the Scottish National Diabetic Retinopathy Screening Programme. *British Journal of Ophthalmology 2014;*98(6):790-795.

5 Konstantakopoulou E, Harper R, Edgar D, et al. Clinical safety of a minor eye conditions scheme in England delivered by community optometrists. *BMJ Open Ophthalmology* 2018;3(1):p.e000125.

6 MacEwen C. 2016. *Increasing demand on hospital eye services risks patients losing vision - The Royal College of Ophthalmologists*. [online] The Royal College of Ophthalmologists. Available at: <https://www.rcophth.ac.uk/2016/03/increasing-demand-on-hospital-eye-services-risks-patients-losing-vision/> [Accessed 9 June 2021].

7 McCormick E. 2017. *OCT rollout in every Specsavers announced*. [online] Aop.org.uk. Available at: <https://www.aop.org.uk/ot/industry/high-street/2017/05/22/oct-rollout-in-every-specsavers-announced> [Accessed 9 June 2021].

8 Kortuem K, Fasler K, Charnley A, et al. Implementation of medical retina virtual clinics in a tertiary eye care referral centre. *British Journal of Ophthalmology* 2018;102(10):1391-1395.

9 De Fauw J, Ledsam J, Romera-Paredes B, et al. Clinically applicable deep learning for diagnosis and referral in retinal disease. *Nature Medicine* 2018;24(9):1342-1350.

10 Campbell M, Piaggio G, Elbourne D, et al. Consort 2010 statement: extension to cluster randomised trials. *BMJ* 2012;345(sep04 1):pp.e5661-e5661.

11 Bossuyt P, Reitsma J, Bruns D, et al. STARD 2015: an updated list of essential items for reporting diagnostic accuracy studies. *BMJ* 2015:p.h5527.

12 Sounderajah V, Ashrafian H, Aggarwal R, et al. Developing specific reporting guidelines for diagnostic accuracy studies assessing AI interventions: The STARD-AI Steering Group. *Nature Medicine* 2020;26(6):807-808.

13 Curtis L and Burns A. *Unit Costs of Health and Social Care 2019*. Canterbury: Personal Social Services Research Unit, University of Kent 2019:p.176.

14 Drummond M. *Methods for the economic evaluation of health care programmes*. Oxford: Oxford University Press 2005.

15 Burr J, Botello-Pinzon P, Takwoingi Y, et al. Surveillance for ocular hypertension: an evidence synthesis and economic evaluation. *Health Technology Assessment* 2012;16(29).

16 McCormack K, Wake B, Perez J, et al. Laparoscopic surgery for inguinal hernia repair: systematic review of effectiveness and economic evaluation. *Health Technology Assessment* 2005;9(14).

17 Shih H, Chou P, Chen S, et al. A Community-based Study of the Willingness to Pay Associated with Screening for Diabetic Retinopathy among Type 2 Diabetes in Kinmen, Taiwan. *Journal of Epidemiology* 2007;17(6):186-193.

18 Furniss D, Iacovides I, Lyons I, et al. Patient and public involvement in patient safety research: a workshop to review patient information, minimise psychological risk and inform research. *Research Involvement and Engagement* 2016;2(1).

For peer review only

CONSORT Tele-Ophthalmology Superiority Cluster Randomised Trial

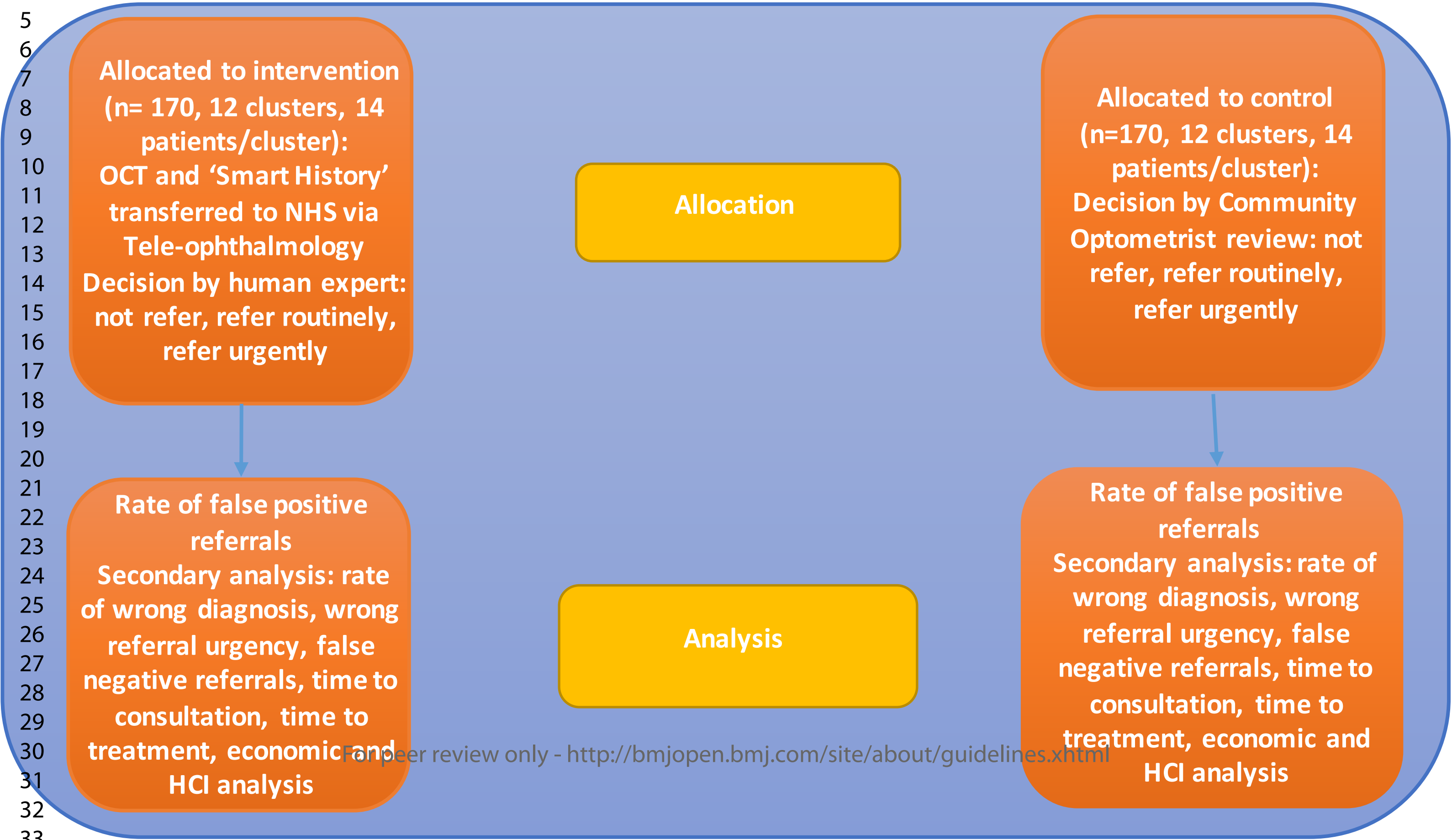

For peer review only - http://bmjopen.bmj.com/site/about/guidelines.xhtml

STARD Artificial Intelligence Observational Prospective Diagnostic Study

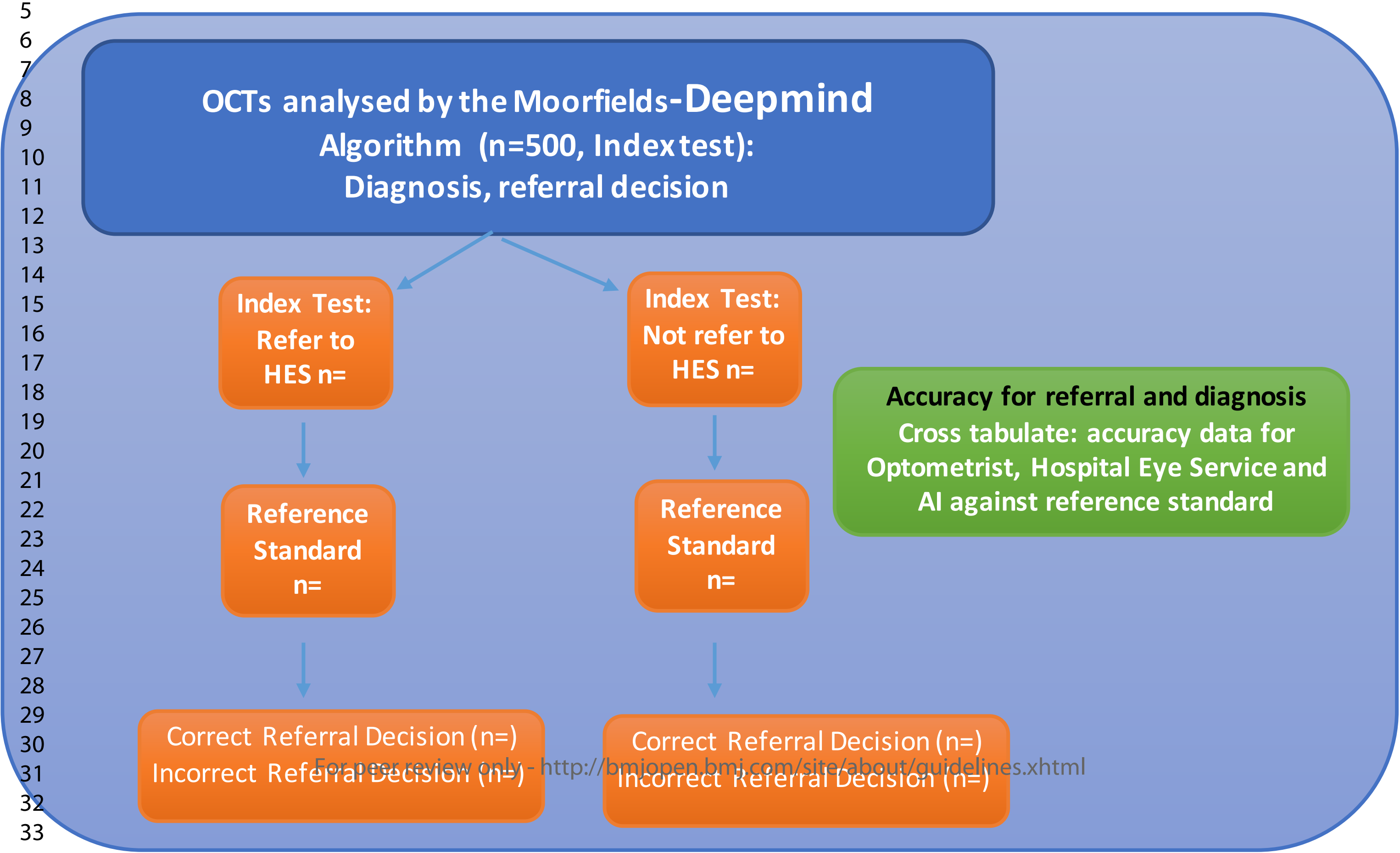

For peer review only - http://bmjopen.bmj.com/site/about/guidelines.xhtml

Appendix

Table of Contents

Appendix 1. Sampling, Recruitment and Analysis Methods

Appendix 2. Participant Information Sheet and Consent Form

Appendix 3. Digital Pathway Decisions

Appendix 4. Statistical Consideration

4.1 Sample Size Calculation

4.2 Statistical analysis

Appendix 5. Case Report Form

Appendix 6. Trial Documents and Subject Records

6.1 CRFs and Source Document Identification

6.2 Confidentiality of Trial Documents and Subject Records

6.3 Procedures for validation and securing of electronic clinical data systems

6.4 Data handling and record keeping

Appendix 7. References

Appendix 1. Sampling, Recruitment and Analysis Methods

Purposive sampling techniques will be applied to recruit participants who are representative of all key patient and professional groups across all sites, including both "power users" and reluctant users. In each setting, the aim will be to interview 15 patients and up to 10 clinicians and other professionals (settings: secondary care clinics; pre-; and post-transitioning optometry practices), depending on the sizes of the clinics selected for inclusion. The interviews will be conducted by a Process Evaluation Specialist, they will be semi-structured and will be designed to address the research objectives outlined above. Probes such as anonymised screenshots from the digital referral platform and illustrative information presentation prototypes from the DeepMind algorithm will be used in interviews to support the exploration of the themes. Interview data will be transcribed and analysed by a qualitative methods expert using inductive Thematic Analysis, with a particular focus on facilitators and barriers to change, and the factors that contribute to successful change. These will include questions around trust in technology and data privacy as well as efficiency and effectiveness and changes in clinician workflow and patient experience. Data gathering and analysis will be interleaved, so that later data gathering is informed by the findings from earlier analysis.

Small-scale ethnographic observations will be conducted in all settings, observing both selected clinician-patient interactions around the diagnostic process (community optometry and HES) and clinician tele-care practices (HES). 3-5 clinician-patient consultations will be observed per setting; debrief interviews with patients will cover the same themes as the interviews with practitioners but be sensitive to the different perspectives of patients and professionals. Detailed field notes will be kept of all observations. This data will also be subjected to thematic analysis, focusing on workflows, variability in workflows, and any problems experienced during the interaction (particularly related to technology use).

Patients will be invited to participate at the time that they receive their appointment letter, so that they have time to consider whether they wish to do so (for informed consent), and to plan their clinic visit time to accommodate a short interview (15 mins approx.) after their appointment. On the day of the visit the investigator will provide the patient information leaflet (PIS; **Appendix 1**) to the patient and go through it highlighting what the purpose of the study is, what it entails if the patient decides to take part and possible advantages and disadvantages and risks of taking part. When the patient has had amble time to read the PIS and ask questions regarding the study, the patient will be asked to sign an informed consent form (ICF; **Appendix 1**). Once the informed consent process is complete a copy of the ICF will be provided to the participant, and the signed form will be filed in the participant's study records. Once the informed consent process is complete, the investigator will record the decision in the case history form.

As this is a cluster randomised clinical trial, randomisation applies at the level of entire community optometry practices. The practices randomised to the intervention arm (tele-ophthalmology) will adopt this pathway for all patient referrals to secondary care as standard practice. Patient-level consent for this study pertains to allowing use of collected data for analysis but participation in the study will not

affect patient-level care. Given the urgent presentation of the patient population we will approach for participation in this study and the fact that patient management will not be influenced by randomisation as described above, a 24-hour minimum period of consideration for patient participation is not warranted. Patients approached for participation will be given the study-specific PIS and adequate time to have any queries addressed by the clinical team before deciding on participation to the study.

Appendix 2. Participant Information Sheet and Consent Form

Patient Information Sheet

Study title: Tele-opHthalmology- enablEd and ARtificial Intelligence-ready referral pathway for coMmunity optomEtry referralS or retinal disease: acluster Randomised Superiority trial with a linked Observational Diagnostic Accuracy Study

Short title: HERMES

Protocol Reference Number: BALK1006

We would like to invite you to take part in our research study. Before you decide we would like you to understand why the research is being done and what it would involve for you. One of our team will go through the information sheet with you and answer any questions you have. We'd suggest this should take about 15 minutes. Talk to others about the study if you wish.

Part 1 tells you the purpose of this study and what will happen to you if you take part. Part 2 gives you more detailed information about the conduct of the study.

Ask us if there is anything that is not clear. Take time to decide whether or not you wish to take part.

Part 1

1. What is the purpose of the study?

Early diagnosis of retinal condition (including Age-related Macular degeneration) is classified using imaging technology called Optical coherence tomography (OCT). This technology is becoming more available in community based optometry practices (high street optometry practices); however, interpreting the imaging scans can require hospital level expertise.
As a result of this, a large proportion of patients with retinal disease are incorrectly referred to hospital based eye clinics for diagnostics and disease management. This has led to an increasing pressure on hospital eye services and can cause delay in access for patients with sight threatening disease.

In this study we will involve patients with suspected retinal condition who have attended an eye examination appointment at a participating community practice. Patients that want to take part will be referred to the hospital eye service via the standard pathway for either: urgent care, routine care or not referred at all depending on the clinical assessment and OCT scan taken by the community optometrist.

The study will seek to show that we can improve patient care by using tele-Ophthalmology technologies to manage the proportion of referrals that do not need to attend hospital eye service for consultations and can be managed safely by community based optometry practices. Half of the optometry practices involved in this study will do the referrals through a 'tele-ophthalmology' process. This means that your eye scans and other clinical information will be reviewed remotely by expert clinicians at corresponding NHS eye hospitals and they will make the referral decision instead of the optometrists.

For peer review only - http://bmjopen.bmj.com/site/about/guidelines.xhtml

Your eye scans may also be processed at a later time by an Artificial Intelligence algorithm that can read the scans and also make a referral decision. The Artificial Intelligence algorithm has been developed previously through the collaboration between Moorfields Eye Hospital and Google/Deepmind. This algorithm will be introduced in the Reading Centre (Moorfields Eye Hospital NHS Trust), all data analysis will be performed within the Reading Centre (Moorfields Eye Hospital NHS Trust) and no research data will be shared or analysed externally to the research team. During this study, the Artificial Intelligence algorithm will analyse the OCT and make a recommendation on whether a referral to the hospital is needed or not; this will then be compared with the referral recommendation made by an expert clinician for your care and will not impact on your care in any way. The actual decision to refer or not will be made by a human expert in every case and not by the algorithm.

Additionally, in a sub-study we want to involve patients with suspicion of retinal disease who are already being referred to the hospital eye services via a tele-ophthalmology platform. The patient's clinical history and OCT scan will be reviewed by experts at the participating hospital eye service and a referral decision will be made remotely. This sub-study is seeking to understand what impact the introduction of tele-ophthalmology has in a real-life setting where tele–ophthalmology is already used to refer patients to the hospital eye services and also in terms of patient care and managing unnecessary referral that are made by the community based practices to the hospital based eye services.

2. Why have I been invited?

You have been invited to take part in this study because you are attending an eye examination appointment at a participating community optometry practice that undergo OCT scans and have been diagnosed with suspicious retinal disease.

3. Do I have to take part?

It is up to you to decide to join the study. We will describe the study and go through this information sheet. If you agree to take part, we will then ask you to sign a consent form. You are free to withdraw at any time without giving a reason. This would not affect the standard of care you receive. If you decide not to take part in the study, you will still have your normal eye examination and an eye scan.

4. What will happen to me if I take part?

If you decide to take part in this study, you (and if applicable a witness) will be asked to sign and date a consent form. The consent form will be signed in the presence of a trained healthcare professional who will also sign and date it. You will be provided a copy of this to keep. After the consent process you will undergo a clinical assessment and have an eye scan (OCT). If you take part in the routine care arm of the study, a referral decision will be made as usual by your community optometrist who will decide whether you will need to be; referred urgently to a hospital eye service,

For peer review only - http://bmjopen.bmj.com/site/about/guidelines.xhtml

routinely or not referred at all. The referral decision will be sent as per standard practice to the corresponding hospital eye service. If you take part in the tele-ophthalmology arm of the study, expert clinicians at the corresponding NHS eye hospital will review your eye scan and other clinical information remotely and will make the referral decision.

You will not have a choice about which arm of the study you will participate in, as the optometry practice will already have been pre-assigned either the routine referral arm or the tele-ophthalmology arm. However this will not influence in any aspect the standard of care you will receive during this visit. You will receive the same care whether you are in the routine care arm or in the tele-ophthalmology arm.
Only your community optometrist or an expert clinician will make a referral decision after reviewing your eye scan and clinical information, however you will not be able to decide who will make the referral decision. Additionally, a computer program (AI) may analyse your eye scan and give its own clinical diagnosis, however this will not influence the referral decision that will be made. By using this computer program we only want to obtain information that will help clinicians to make a better clinical decision and diagnosis in the future for people with retinal disease.

If you decide to take part in the sub-study, you will undergo the consent process as described above. After the consent process you will undergo a clinical assessment and have an eye scan (OCT). Your community optometrist will make a referral decision as usual who will decide whether you need to be referred urgently to a hospital eye service, routinely or not referred at all. After reviewing your eye scan, your community optometrist will send the referral via tele-ophthalmology to the corresponding NHS eye hospital. Expert clinicians at the corresponding NHS eye hospital will review your eye scan and other clinical information remotely and will make the referral decision.

5. What are the possible risks and benefits of taking part?

This study has no invasive testing and no therapeutic interventions; therefore there is minimal risk to patients if they choose to participate in this study. Your eyes may be dilated during your visit for the OCT scan. This will make you sensitive to light, blur your vision and may make it difficult to focus on close up objects for 4-6 hours.

We cannot promise the study will help you, but the information we get from this study will help improve the experience of care of people with retinal disease and improve the referral pathway between community optometry practices and the hospital eye service.

6. What if there is a problem?
Any complaint about the way you have been dealt with during the clinical trial or any possible harm you might suffer will be addressed. The detailed information concerning this is given in Part 2 of this information sheet. If you have any concerns or complaints you should contact your study doctor in the first instance.

7. Will my taking part in the study be kept confidential?
Yes. We will follow ethical and legal practice and all information about you will be handled in confidence. The details are included in Part 2.

For peer review only

For peer review only - http://bmjopen.bmj.com/site/about/guidelines.xhtml

8. Contact Details

Principal investigator

Name: Tel. Number:

Research Project Manager

Name: Tel. Number:

This completes Part 1 of the Information Sheet.

If the information in Part 1 has interested you and you are considering participation, please read the additional information in Part 2 before making any decision.

Part 2

9. What if relevant new information becomes available?

On receiving new information, we might consider it to be in your best interests to withdraw you from the study. If so, we will explain the reasons and arrange for your care to continue. If the study is stopped for any other reason, we will tell you why and arrange your continuing care.

10. What will happen if I don’t want to carry on with the study?

If you withdraw from the study, we will destroy all your identifiable information, but we will need to use the data collected up to your withdrawal.

11. What if there is a problem?

If you have a concern about any aspect of this study, you should ask to speak to the researchers who will do their best to answer your questions …. If you remain unhappy and wish to complain formally, you can do this by contacting the PALS team at ….
If taking part in this research project harms you, there are no special compensation arrangements. If you are harmed due to someone’s negligence, then you may have grounds for legal action but you may have to pay for it. Regardless of this, if you wish to complain about any aspect of the way you have been approached or treated during the course of this study, the normal National Health Service complaints mechanisms will be available to you.

12. Will use of my data meet the GDPR rules?

Yes, all data will be handled in accordance with the General Data Protection Regulations (GDPR) and UK Data Protection Act 2018, The Research Governance

For peer review only - http://bmjopen.bmj.com/site/about/guidelines.xhtml

Framework for Health and Social Care and the conditions for the Research Ethics Committee favorable opinion.

Moorfields Eye Hospital NHS Trust is the Sponsor for this study based in the United Kingdom. We will be using information from you and/or your medical records in order to undertake this study and will act as the data controller for this study. This means that we are responsible for looking after your information and using it properly.

Your rights to access, change or move your information are limited, as we need to manage your information in specific ways in order for the research to be reliable and accurate. If you withdraw from the study, we will keep the information about you that we have already obtained.

You can find out more about how we use your information by visiting www.moorfields.nhs.uk/content/how-we-use-your-information or please contact your research team (study team contact details can be found in your participant information sheet).

(SITE NAME) will use your name, and contact details to contact you about the research study, and make sure that relevant information about the study is recorded for your care, and to oversee the quality of the study. Moorfields Eye Hospital and their study collaborators (which may include Universities) will receive information from your medical record for the purposes of the study but without your name or any other personal details. (SITE NAME) will pass to Moorfields Eye Hospital this information collected from you and/or your medical records. The only people in (SITE NAME) who will have access to information that identifies you will be people who need to contact you to arrange appointments or audit the data collection process. The people who analyse the information will not be able to identify you and will not be able to find out your name or contact details. Data collected for this study, without your name or any other personal details, can be used for future research. If you agree to take part in this study, we will also specifically ask for your permission to use your data for future research. If you don't want your data to be used for any future research, you can still participate in this study.

Moorfields Eye Hospital will keep identifiable information about you from this study without your personal details for 15 years.

13. Will my GP be informed of my involvement?

With your permission, your GP, and other doctors who may be treating you, will be notified that you are taking part in this study.

14. What will happen to the results of the research study?

The results of the study will be available after it finishes and will usually be published in a medical journal or be presented at a scientific conference. The data will be anonymous and none of the patients involved in the trial will be identified in any report or publication.

Should you wish to see the results, or the publication, please ask your study doctor.

15. Who is organising and funding the research?

For peer review only

For peer review only - http://bmjopen.bmj.com/site/about/guidelines.xhtml

The study is organized by Moorfields Eye Hospitals NHS Foundation Trust and funded by the National Institute for Health Research.

16. Who has reviewed the study?

All research in the NHS is looked at by independent group of people, called a Research Ethics Committee, to protect your interests. This study has been reviewed and given favourable opinion by London – Bromley Research Ethics Committee.

17. Further information and contact details

You are encouraged to ask any questions you wish, before, during or after your treatment. If you have any questions about the study, please speak to your study Optometrists or doctor, who will be able to provide you with up to date information about the procedure(s) involved. If you wish to read the research on which this study is based, please ask your study optometrists or doctor. If you require any further information or have any concerns while taking part in the study please contact one of the following people:

Principle Investigator

Name: Tel. Number:

Research Project Manager

Name: Tel. Number:

If you decide you would like to take part then please read and sign the consent form. You will be given a copy of this information sheet and the consent form to keep. A copy of the consent form will be filed in your patient notes, one will be filed with the study records and one may be sent to the Research Sponsor.

You can have more time to think this over if you are at all unsure.

Thank you for taking the time to read this information sheet and to consider this study.

For peer review only

For peer review only - http://bmjopen.bmj.com/site/about/guidelines.xhtml

Appendix 3. Digital Pathway Decisions

The following scenarios can occur in the intervention arm:

1. Community optometrist recommendation: **Refer urgently to HES**—> OCT scan and clinical data are transferred to ‘tele-HES’ and reviewed within 48h remotely by human expert —> Referral Decision is made in ‘tele-HES’ (refer urgently, refer routinely, don’t refer) and fed-back to the community optometry practice to be implemented.
2. Community optometrist recommendation: **Refer routinely to HES**—> OCT scan and clinical data are transferred to ‘tele-HES’ and reviewed within 48h remotely by human expert —> Referral Decision is made in ‘tele-HES’ (refer urgently, refer routinely, don’t refer) and fed-back to the community optometry practice to be implemented.
3. Community optometrist recommendation: **Don’t refer to HES**—> OCT scan and clinical data are transferred to ‘tele-HES’ and reviewed within 48h remotely by human expert —> Referral Decision is made in ‘tele-HES’ (refer urgently, refer routinely, don’t refer) and fed-back to the community optometry practice to be implemented.

Appendix 4. Statistical Consideration

The trial statisticians based at King's Clinical Trial Unit will write the statistical analysis plan before database lock and will perform the analysis using the Stata software (StataCorp, College Station, TX, USA).

4.1 Sample Size Calculation

The primary outcome is the proportion of false positive referrals. Under the current system an audit conducted at Moorfields Eye Hospital NHS Foundation Trust in September 2018 showed that 70 % of retinal referrals were false positive.[1] A pilot study on 40 patients conducted in three optometry practices showed that this could be reduced by 60 %.[1] A 95 % confidence interval computed by the modified Wald Method as advised by Agresti and Coull would extend 44.6% to 73.7%.[2] There is consensus amongst clinicians however that given the savings to the NHS and benefit to patients, slightly smaller differences would be important to detect and we have powered the study to examine a reduction to 40 % false referrals. Whilst smaller differences might yet be important it would seem unethical to power for lower than 40 % based on the observed data and clinical expertise in this area.

Although decisions for patients are made on an individual basis each patient cannot be assumed to generate independent information since they will be clustered within optometry practices. The correlation of information from patients within a cluster (the intracluster correlation) is estimated to be 0.15. We have based this intracluster correlation on previous work conducted in ophthalmology [3, 4] with a clinical outcome similar to this study. Since this is an estimate we have increased the intracluster correlation slightly to allow for the potential that patients within the same optometry practice may be more similarly managed than patients within different practices although clinical consensus is that clinical signs are more likely to impact upon decision making for referral than individual optometrist attitudes. Using nQuery software version 8.3.10, a hierarchical 2-level mixed effects model was used to calculate the required sample size. 24/26 clusters split between the study arms in a 1:1 ratio need to recruit an average of 12/10 patients per clusters (12 patients if 24 clusters, 10 patients if 26 clusters) in order to achieve 89.27% power to detect a difference in the proportion of false positive referrals of 30% (a drop from the current rate of 70% to the clinically relevant rate of 40%). This calculation assumes an intracluster correlation of 0.15 and the test is performed at the 5% significance level.

A total of 288 patients (based on an average of 12 patients recruited at 24 clusters, 144 per study arm) would therefore be needed to complete the data analysis with sufficient statistical power. To allow for an anticipated 15% drop our rate (patients are likely to be elderly and have comorbidity causing motion artefacts and some images may be ungradable), the total sample size is 340 patients (170 per study arm).

The sample size of the RCT and pragmatic sub-study combined will also enable the AI observational diagnostic accuracy study to obtain robust estimates of sensitivity and specificity. All 500 patients (accounting for the anticipated drop-out rate) will be included in the AI study. Classifications will be

made without additional clinical information. Research from our group suggests that the diagnostic accuracy of the Moorfields-DeepMind AI will be as high as 95%.[5] This combined sample of 500 patients with 475 patients being correctly diagnosed would produce a two-sided 95.0% confidence interval with a width of 0.039. The sample from the RCT alone -288 patients with 274 being correctly diagnosed - produce a two-sided 95.0% confidence interval with a width of 0.052. PASS has been used to calculated these widths.

Recruitment plan

On the basis of feedback provided by optometry practices already identified and interested in participating in the study, an average of 3 eligible patients can be approached to consider participation in the study per month per cluster, with a range of 2-5 patients based on the size of the optometry practice. However, it is also expected that 35% of potential patients will decline to participate. Currently, different sites are at different stages of readiness for commencing recruitment and therefore a staggered start to recruitment over 3 months is embedded in the recruitment plan. Based on these conservative estimates a recruitment period of 12 months with a staggered initiation over the first 3 months will be sufficient to approach 521 patients, of whom it is expected that 340 patients will be recruited to the study. A smaller practice only approaching 2 patients per month will require 11 months to recruit 12 patients (accounting for drop out and decline to participate). If the practice was one of the last to start recruitment and so started 3 months into the recruitment window, they would still manage to recruit 10 patients in 9 months (accounting for drop out and decline to participate). A larger practice in the same catchment area will be able to compensate by over-recruiting up to a set maximum of 16 patients. The range of cluster size will thus be 10-16 patients.

The 2 additional randomised community practices allow the potential to increase the number of clusters to 26 if further acceleration of recruitment is required. 26 clusters will be required to recruit 306 patients overall with an average of 10 patients per cluster (and a minimum of 8 patients per cluster) in order to achieve 89.27% power to detect a difference in the proportion of false positive referrals of 30% - using the same parameters as the sample size calculation for the 24 clusters.

Pragmatic Sub-Study

Manchester Eye Hospital and its local area, a site included in the original protocol, has already moved to a tele-ophthalmology referral pathway as part of a commissioning change across the local region. This change in standard care provides a unique opportunity to examine whether tele-ophthalmology works under usual conditions within the NHS. This sub-study will allow us to measure and visualise variation in quality of health care within a local region to inform our inferences from the RCT on how the tele- ophthalmology pathway will perform within a real-life setting.

From this study, key estimate statistics will be calculated including the overall rate of referral to HES, the false positive (referral) and false negative rate against the Reference Standard and the proportion of wrong diagnosis and wrong referral urgency. These shall be compared to the rates found for the intervention arm of the main RCT.

Recruiting 18 patients from each of 12 tele-optometry practices (for a total of 216 patients) will allow the proportion of false positive referrals to be produced with a 95% confidence interval with a width less than 0.187. This was calculated based on confidence intervals for one proportion within a cluster-randomised design with an intracluster correlation of 0.15. A total of 216 patients (based on an average of 18 patients recruited from 12 clusters) would therefore provide a certain degree of precision. To allow for an anticipated 15% drop our rate (patients are likely to be elderly and have comorbidity causing motion artefacts and some images may be ungradable), the total sample size is 254 patients. It is expected that 35% of participants will decline to participate and so 390 patients would need to be approached. These patients can be recruited over a period of 18 months with an average of 3 patients approached by each practice per month.

4.2 Statistical analysis

The primary analysis will be conducted following an intention to treat principle where all randomised patients are analysed in their allocated group whether or not they receive their randomised management plan. Baseline characteristics will be summarised for each management group (standard care or tele-ophthalmology). We will report the number of clusters in each group and the size of clusters. Continuous data will be summarised using means and standard deviations if data appear Gaussian or medians and interquartile ranges. Categorical data will be reported as proportions and percentages.

The primary outcome is the proportion of false referrals. The outcome is measured at the patient level. This will be compared between management groups using logistic regression adjusting for clustered centres. Outcomes will be reported as adjusted odds ratios. We will also report the difference in proportions with a 95 % confidence interval as per the CONSORT extension for cluster randomised controlled trials. We will report false referral rates with 95 % confidence intervals computed by the exact binomial method by diagnosis and by level of urgency. The results will be presented at the cluster level and overall.

Secondary outcomes such as time from referral to review in HES and treatment will be analysed in a similar fashion. The percentage of patients experiencing adverse events in the two groups will be reported with 95 % confidence intervals computed by the exact binomial method.

Loss to follow-up will be examined by study arm. Reasons for missingness may be important and these will be investigated using logistic regression of covariates based on an indicator of missingness. An available case analysis will be reported along with an analysis using imputed data based on best- and worst-case scenarios. Since this is a cluster RCT we will also examine and report missingness by cluster.

No formal interim analysis is planned but reports concerning patient safety will be prepared for review by the Independent Data Monitoring Committee. All tests will be two sided and will be assessed at the 5 % significance level unless otherwise specified. All confidence intervals will be 95 % and two sided. A detailed statistical analysis plan will be agreed with the Trial Steering Group prior to any analysis of locked data. All statistical analysis will be performed using Stata (StataCorp, College Station, TX, USA).

Statisticians analysing the data will be masked to the management group status of the practise and patient.

In the AI diagnostic accuracy study, we will report estimates of sensitivity and specificity of the DeepMind algorithm for referral decisions with 95 % confidence intervals. Our primary analysis will combine urgent and standard referral to HES and compare against no referral to HES but a sensitivity analysis will be conducted to evaluate urgent referrals. The referral outcome (refer routinely, refer urgently, don't refer) will be cross tabulated for the DeepMind algorithm and each of the RCT treatment arms (community optometry and 'tele-HES'), the pragmatic sub-study, and for the Reference Standard.

Appendix 5. Case Report Form

Moorfields Eye Hospital NHS Foundation Trust NHS

HERMES Study
Completing Case Report Forms (CRFs)

Study No: ☐☐ - ☐☐☐

This document has been created to provide guidelines about completing clinical trial case report forms at Moorfields Eye Hospital (MEH). The information has been extracted from the standard operating procedures (SOPs) 'Completing, Correcting & Signing off Case Report Forms (CRF_S07)' that have been developed by the Research & Development department at Moorfields.

1. The CRF must be completed as soon as possible after the patient has been assessed or during the assessment if the CRF is the source data.

2. CRFs must be completed using a black ink ballpoint pen.

3. If the CRF is printed on carbonless duplication paper, a suitable separator must be inserted under the form being completed.

4. Data entry into the CRF must be complete as without omissions. If data are unavailable then 'unknown', 'missing', 'test not done' etc. should be inserted. The ambiguous phrase, 'not available' should be avoided.

5. All entries into the CRF must be accurate, legible and verifiable with the source data in the medical records (unless the CRF is the source data). Data must not be invented – this is fraud.

   N.B. Whenever a subject has been seen by clinical staff for the purposes of a clinical trial, the time, date and reason for visit must always be entered into the subject's corresponding hospital notes. Copies of trial investigations/results that are clinically significant or have an impact on the patient's clinical care must also be filed in the medical notes.

6. Any discrepancies between the CRF and the source data should be explained and the significance noted in the CRF and/or patient's medical records.

7. All CRF data derived from source documents must be transcribed exactly.

8. For laboratory values that fall outside the laboratory's reference range or trial specific range or when a value shows a significant variation from one assessment to the next, this should be commented on and the significance noted in the CRF and/or patient's medical records.

9. The subject's identity should remain confidential at all times and as such the trial subject must only be identified in the CRF using a trial number or code.

10. Entries into the CRF must never be overwritten.

11. Corrections to the CRF must be made as follows:

    - An incorrect entry must be deleted with a single line through the text allowing the incorrect entry to remain legible. Correction fluid must never be used and entries must not be obliterated.
    - The correct data must be entered.
    - The correction must be initialled and dated and an explanation given of the correction, if applicable.

12. The CRF must be signed and dated where indicated, by the chief/principal investigator or designee (for example, research nurse at the end of an assessment) to assert that he/she believes the data is completed and correct.

For peer review only - http://bmjopen.bmj.com/site/about/guidelines.xhtml

Moorfields Eye Hospital, Case Report Form
BALK1006

Moorfields Eye Hospital NHS Foundation Trust NHS

Study No: ☐☐ - ☐☐☐

HERMES Study

PATIENT DEMOGRAPHICS (Community Optometry) **Page 1/2**

ALL FIELDS ARE MANDATORY

**Patient Details**		
Study site	MEH ☐ Manchester ☐ Birmingham ☐<br>Central Middlesex ☐ North West Anglia ☐	
Optometrist site		
Randomisation Cluster number		
Randomisation Arm	Control ☐	Intervention ☐
Age		*(≥18)* ☐☐☐
Sex	Male ☐	Female ☐
Medical history	Yes No<br>Heart attack ☐ ☐<br>COPD ☐ ☐<br>Diabetes ☐ ☐<br>Hypertension ☐ ☐	Yes No<br>TIA/Stroke ☐ ☐<br>Impaired Mobility ☐ ☐<br>Asthma ☐ ☐<br>Other ☐ ☐
If other please specify		
Medication for eye condition	Yes ☐	No ☐
If yes specify glaucoma drops?	Prostaglandins ☐ CA inhibitors ☐	B-blockers ☐
If yes specify AREDS	Yes ☐	No ☐
Smoker?	Ex-smoker ☐ Smoker ☐	Non-smoker ☐
	**Right Eye**	**Left eye**
Ocular history	Yes No<br>Wet AMD ☐ ☐<br>Dry AMD ☐ ☐<br>Central Serous Chorioretinopathy ☐ ☐<br>Central Retinal Vein Occlusion ☐ ☐<br>Branch Retinal Vein Occlusion ☐ ☐<br>Diabetic Macular Oedema ☐ ☐<br>Inherited Eye Disease ☐ ☐<br>Other ☐ ☐	Yes No<br>Wet AMD ☐ ☐<br>Dry AMD ☐ ☐<br>Central Serous Chorioretinopathy ☐ ☐<br>Central Retinal Vein Occlusion ☐ ☐<br>Branch Retinal Vein Occlusion ☐ ☐<br>Diabetic Macular Oedema ☐ ☐<br>Inherited Eye Disease ☐ ☐<br>Other ☐ ☐
If other, specify		

For peer review only

For peer review only - http://bmjopen.bmj.com/site/about/guidelines.xhtml

Moorfields Eye Hospital, Case Report Form
BALK1006

Moorfields Eye Hospital NHS Foundation Trust — NHS

Study No: ☐☐ - ☐☐☐

HERMES Study

PATIENT DEMOGRAPHICS (Community Optometry) **Page 2/2**

ALL FIELDS ARE MANDATORY

Patient Details

		Yes	No		Yes	No
Previous eye procedures	Cataract surgery	☐	☐	Cataract surgery	☐	☐
	Glaucoma surgery	☐	☐	Glaucoma surgery	☐	☐
	Eyelid surgery	☐	☐	Eyelid surgery	☐	☐
	Other	☐	☐	Other	☐	☐
If other specify						

Comments

For peer review only

I can confirm that the patient meets all eligibility criteria for the study and I have completed this form in full and take full responsibility for any missing data.

**Signed:**	**Print:**	**Date:**
		☐☐ / ☐☐ / ☐☐☐☐

Office use only, data entry completed by:

**Signed:**	**Print:**	**Date:**
		☐☐ / ☐☐ / ☐☐☐☐

For peer review only - http://bmjopen.bmj.com/site/about/guidelines.xhtml

Moorfields Eye Hospital, Case Report Form
BALK1006

Moorfields Eye Hospital NHS Foundation Trust NHS

Study No: ☐☐ - ☐☐☐

HERMES Study

BASELINE VISIT (Community Optometry) **Page 1/2**

ALL FIELDS ARE MANDATORY

**Baseline Exam**		
Date of visit	*(dd mm yyyy)* ☐☐ / ☐☐ / ☐☐☐☐	
Years since diagnosis of any retinal disease?	☐☐	
OCT Device	Topcon 3D OCT-2000 ☐ Heidelberg OCT1 ☐ Other ☐	
If other specify		
	**Right Eye**	**Left eye**
Visual acuity (ETDRS)	*(ETDRS letters, range 0-95)* ☐☐	*(ETDRS letters, range 0-95)* ☐☐
Visual acuity (Snellen)	☐ / ☐☐	☐ / ☐☐
Diagnosis by the Optometrist	Yes No<br>Wet AMD ☐ ☐<br>Dry AMD ☐ ☐<br>Central Serous Chorioretinopathy ☐ ☐<br>Central Retinal Vein Occlusion ☐ ☐<br>Branch Retinal Vein Occlusion ☐ ☐<br>Diabetic Macular Oedema ☐ ☐<br>Inherited Eye Disease ☐ ☐<br>Other ☐ ☐	Yes No<br>Wet AMD ☐ ☐<br>Dry AMD ☐ ☐<br>Central Serous Chorioretinopathy ☐ ☐<br>Central Retinal Vein Occlusion ☐ ☐<br>Branch Retinal Vein Occlusion ☐ ☐<br>Diabetic Macular Oedema ☐ ☐<br>Inherited Eye Disease ☐ ☐<br>Other ☐ ☐
If other specify		
Clinical findings	Yes No<br>Macular Haemorrhage ☐ ☐<br>Other RetinalHaemorrhage ☐ ☐<br>Exudates ☐ ☐<br>Disc Swelling ☐ ☐<br>Macular Atrophy ☐ ☐<br>Cotton Wool Spot ☐ ☐<br>Other ☐ ☐	Yes No<br>Macular Haemorrhage ☐ ☐<br>Other RetinalHaemorrhage ☐ ☐<br>Exudates ☐ ☐<br>Disc Swelling ☐ ☐<br>Macular Atrophy ☐ ☐<br>Cotton Wool Spot ☐ ☐<br>Other ☐ ☐
If other specify		

For peer review only

For peer review only - http://bmjopen.bmj.com/site/about/guidelines.xhtml

Moorfields Eye Hospital, Case Report Form
BALK1006

Moorfields Eye Hospital NHS Foundation Trust NHS

Study No: ☐☐ - ☐☐☐

HERMES Study

BASELINE VISIT (Community Optometry) **Page 2/2**

ALL FIELDS ARE MANDATORY		
**Baseline Exam**	**Right Eye**	**Left eye**
Intraocular pressure	*(0-60)* ☐☐	*(0-60)* ☐☐
OCT taken?	Yes ☐ No ☐	Yes ☐ No ☐
OCT qualitative	SRF ☐ IRF ☐ PED ☐ SHRM ☐	SRF ☐ IRF ☐ PED ☐ SHRM ☐
Referral Recommendation by Optometrist (both arms)	Urgent ☐ Routine ☐ No referral ☐	
If not referred, specify reason		

Comments:

For peer review only

I have completed this form in full and take full responsibility for any missing data.

**Signed:**	**Print:**	**Date:**
		☐☐ / ☐☐ / ☐☐☐☐
**Office use only, data entry completed by:**		
**Signed:**	**Print:**	**Date:**
		☐☐ / ☐☐ / ☐☐☐☐

For peer review only - http://bmjopen.bmj.com/site/about/guidelines.xhtml

Moorfields Eye Hospital, Case Report Form
BALK1006

Moorfields Eye Hospital NHS Foundation Trust NHS

Study No: ☐☐ - ☐☐☐

HERMES Study

REFERRALS (Community Optometry) **Page 1/3**

**ALL FIELDS ARE MANDATORY**	
**Referral Details**	
Has referral been generated?	***(control arm)*** Yes ☐ No ☐
If yes please specify date	***(dd mm yyyy)*** ☐☐ / ☐☐ / ☐☐☐☐
Referral reference number	***(control arm)***
Referral system	***(control arm)*** Post ☐ Electronic ☐ Other ☐
If other, please specify	
Have OCT scans been uploaded on eCRF?	***(both arms)*** Yes ☐ No ☐
If no, specify reason	
Has clinical data been uploaded on eCRF?	***(intervention arm)*** Yes ☐ No ☐
If no, specify reason	
Optometrist referral recommendation accepted after HES triage?	***(both arms)*** Yes ☐ No ☐
If yes, eye on which referral decision was based	Right ☐ Left ☐ Both ☐
If no, referral decision after HES triage	Urgent ☐ Routine ☐ No referral ☐
Reason why recommendation was not accepted ***(field to be completed by HES)***	

For peer review only

For peer review only - http://bmjopen.bmj.com/site/about/guidelines.xhtml

Moorfields Eye Hospital, Case Report Form
BALK1006

Moorfields Eye Hospital NHS Foundation Trust NHS

Study No: ☐☐ - ☐☐☐

HERMES Study

REFERRALS (tele-HES) **Page 2/3**

ALL FIELDS ARE MANDATORY		
**Tele - HES Review**		
OCT from referral reviewed by tele-HES?	Yes ☐	No ☐
	**Right Eye**	**Left eye**
OCT from referral *qualitative by tele-HES*	SRF ☐ IRF ☐<br>PED ☐ SHRM ☐	SRF ☐ IRF ☐<br>PED ☐ SHRM ☐
Clinical findings	Yes No<br>Macular Haemorrhage ☐ ☐<br>Other Retinal Haemorrhage ☐ ☐<br>Exudates ☐ ☐<br>Disc Swelling ☐ ☐<br>Macular Atrophy ☐ ☐<br>Cotton Wool Spot ☐ ☐<br>Other ☐ ☐	Yes No<br>Macular Haemorrhage ☐ ☐<br>Other Retinal Haemorrhage ☐ ☐<br>Exudates ☐ ☐<br>Disc Swelling ☐ ☐<br>Macular Atrophy ☐ ☐<br>Cotton Wool Spot ☐ ☐<br>Other ☐ ☐
If other specify		
**Tele-HES Review**	**Right Eye**	**Left eye**
Diagnosis from referring optometrist confirmed by tele-HES	Yes ☐	No ☐
Diagnosis by tele-HES	Yes No<br>Wet AMD ☐ ☐<br>Dry AMD ☐ ☐<br>Central Serous Chorioretinopathy ☐ ☐<br>Central Retinal Vein Occlusion ☐ ☐<br>Branch Retinal Vein Occlusion ☐ ☐<br>Diabetic Macular Oedema ☐ ☐<br>Inherited Eye Disease ☐ ☐<br>Other ☐ ☐	Yes No<br>Wet AMD ☐ ☐<br>Dry AMD ☐ ☐<br>Central Serous Chorioretinopathy ☐ ☐<br>Central Retinal Vein Occlusion ☐ ☐<br>Branch Retinal Vein Occlusion ☐ ☐<br>Diabetic Macular Oedema ☐ ☐<br>Inherited Eye Disease ☐ ☐<br>Other ☐ ☐
If other specify		

For peer review only

For peer review only - http://bmjopen.bmj.com/site/about/guidelines.xhtml

Moorfields Eye Hospital, Case Report Form
BALK1006

Moorfields Eye Hospital NHS Foundation Trust NHS

Study No: ☐☐ - ☐☐☐

HERMES Study

REFERRALS (tele-HES) **Page 3/3**

**ALL FIELDS ARE MANDATORY**			
**Tele - HES Review**			
Referral recommendation by referring optometrist confirmed by tele-HES		Yes ☐	No ☐
If yes, eye on which referral decision was based	Right ☐	Left ☐	Both ☐
Referral Decision by tele-HES	Urgent ☐	Routine ☐	No referral ☐

Comments:

For peer review only

I have completed this form in full and take full responsibility for any missing data.

**Signed:**	**Print:**	**Date:**
		☐☐ / ☐☐ / ☐☐☐☐
**Office use only, data entry completed by:**		
**Signed:**	**Print:**	**Date:**
		☐☐ / ☐☐ / ☐☐☐☐

For peer review only - http://bmjopen.bmj.com/site/about/guidelines.xhtml

Moorfields Eye Hospital NHS Foundation Trust NHS

Study No: ☐☐ - ☐☐☐

HERMES Study

HES First Visit **Page 1/2**

**ALL FIELDS ARE MANDATORY**		
**HES Review**		
Follow up status	Attended ☐ Cancelled ☐ DNA ☐	
Date of consultation	*(dd mm yyyy)* ☐☐/☐☐/☐☐☐☐	
Change in eye medication	Yes ☐ No ☐	
If yes, specify		
Date of first treatment (if applicable)	*(dd mm yyyy)* ☐☐/☐☐/☐☐☐☐	
	**Right Eye**	**Left eye**
Intraocular pressure	*(0-60)* ☐☐	*(0-60)* ☐☐
OCT taken?	Yes ☐ No ☐	Yes ☐ No ☐
OCT qualitative	SRF ☐ IRF ☐<br>PED ☐ SHRM ☐	SRF ☐ IRF ☐<br>PED ☐ SHRM ☐
Visual acuity (ETDRS)	*(ETDRS letters, range 0-95)* ☐☐	*(ETDRS letters, range 0-95)* ☐☐
Visual acuity (Snellen)	☐ / ☐☐	☐ / ☐☐
Clinical findings by HES	Yes No<br>Macular Haemorrhage ☐ ☐<br>Other Retinal Haemorrhage ☐ ☐<br>Exudates ☐ ☐<br>Disc Swelling ☐ ☐<br>Macular Atrophy ☐ ☐<br>Cotton Wool Spot ☐ ☐<br>Other ☐ ☐	Yes No<br>Macular Haemorrhage ☐ ☐<br>Other Retinal Haemorrhage ☐ ☐<br>Exudates ☐ ☐<br>Disc Swelling ☐ ☐<br>Macular Atrophy ☐ ☐<br>Cotton Wool Spot ☐ ☐<br>Other ☐ ☐
If other specify		
Diagnosis by HES	Yes No<br>Wet AMD ☐ ☐<br>Dry AMD ☐ ☐<br>Central Serous Chorioretinopathy ☐ ☐<br>Central Retinal Vein Occlusion ☐ ☐<br>Branch Retinal Vein Occlusion ☐ ☐<br>Diabetic Macular Oedema ☐ ☐<br>Inherited Eye Disease ☐ ☐<br>Other ☐ ☐	Yes No<br>Wet AMD ☐ ☐<br>Dry AMD ☐ ☐<br>Central Serous Chorioretinopathy ☐ ☐<br>Central Retinal Vein Occlusion ☐ ☐<br>Branch Retinal Vein Occlusion ☐ ☐<br>Diabetic Macular Oedema ☐ ☐<br>Inherited Eye Disease ☐ ☐<br>Other ☐ ☐
If other specify		

For peer review only - http://bmjopen.bmj.com/site/about/guidelines.xhtml

Moorfields Eye Hospital, Case Report Form
BALK1006

Moorfields Eye Hospital NHS Foundation Trust NHS

Study No: ☐☐ - ☐☐☐

HERMES Study

HES First Visit **Page 2/2**

ALL FIELDS ARE MANDATORY		
**HES Review**	**Right Eye**	**Left eye**
	Yes No	Yes No
Additional Diagnostic Procedures	OCT - Angio ☐ ☐	OCT - Angio ☐ ☐
	IGCA ☐ ☐	IGCA ☐ ☐
	FA ☐ ☐	FA ☐ ☐
	Optos ☐ ☐	Optos ☐ ☐
	Ultrasound B Scan ☐ ☐	Ultrasound B Scan ☐ ☐
	Visual Field Test ☐ ☐	Visual Field Test ☐ ☐
	Other ☐ ☐	Other ☐ ☐
If other specify		

Comments:

I have completed this form in full and take full responsibility for any missing data.

**Signed:**	**Print:**	**Date:**
		☐☐/☐☐/☐☐☐☐
**Office use only, data entry completed by:**		
**Signed:**	**Print:**	**Date:**
		☐☐/☐☐/☐☐☐☐

For peer review only - http://bmjopen.bmj.com/site/about/guidelines.xhtml

Moorfields Eye Hospital, Case Report Form
BALK1006

Moorfields Eye Hospital NHS Foundation Trust NHS

Study No: ☐☐ - ☐☐☐

HERMES Study
READING CENTRE **Page 1/2**

ALL FIELDS ARE MANDATORY

Reference Standard	Right Eye	Left eye
Diagnosis by the reference standard	Yes No<br>Wet AMD ☐ ☐<br>Dry AMD ☐ ☐<br>Central Serous Chorioretinopathy ☐ ☐<br>Central Retinal Vein Occlusion ☐ ☐<br>Branch Retinal Vein Occlusion ☐ ☐<br>Diabetic Macular Oedema ☐ ☐<br>Inherited Eye Disease ☐ ☐<br>Other ☐ ☐	Yes No<br>Wet AMD ☐ ☐<br>Dry AMD ☐ ☐<br>Central Serous Chorioretinopathy ☐ ☐<br>Central Retinal Vein Occlusion ☐ ☐<br>Branch Retinal Vein Occlusion ☐ ☐<br>Diabetic Macular Oedema ☐ ☐<br>Inherited Eye Disease ☐ ☐<br>Other ☐ ☐
If other specify		
Referral decision by reference standard	Urgent ☐ Routine ☐ No referral ☐	
**Artificial Intelligence Moorfields DeepMind**	**Right Eye**	**Left eye**
Deep-Mind Diagnosis of retinal disease	Yes No<br>Wet AMD ☐ ☐<br>Dry AMD ☐ ☐<br>Central Serous Chorioretinopathy ☐ ☐<br>Central Retinal Vein Occlusion ☐ ☐<br>Branch Retinal Vein Occlusion ☐ ☐<br>Diabetic Macular Oedema ☐ ☐<br>Inherited Eye Disease ☐ ☐<br>Other ☐ ☐	Yes No<br>Wet AMD ☐ ☐<br>Dry AMD ☐ ☐<br>Central Serous Chorioretinopathy ☐ ☐<br>Central Retinal Vein Occlusion ☐ ☐<br>Branch Retinal Vein Occlusion ☐ ☐<br>Diabetic Macular Oedema ☐ ☐<br>Inherited Eye Disease ☐ ☐<br>Other ☐ ☐
If other specify		
Referral decision by Moorfields DeepMind	Urgent ☐ Routine ☐ No referral ☐	

For peer review only - http://bmjopen.bmj.com/site/about/guidelines.xhtml

Moorfields Eye Hospital, Case Report Form
BALK1006

Moorfields Eye Hospital NHS Foundation Trust NHS

Study No: ☐☐-☐☐☐

HERMES Study

READING CENTRE

Page 2/2

**ALL FIELDS ARE MANDATORY**	
**Artificial Intelligence Moorfields-DeepMind**	
Time from receiving the OCT scans and a referral decision (hours)	*(hours)* ☐☐
End-to-end inference speed of technical infrastructure supporting the AI DSS	*(minutes)* ☐☐
Any technical issues	Yes ☐ No ☐
If yes, specify details	

Comments:

I have completed this form in full and take full responsibility for any missing data.

**Signed:**	**Print:**	**Date:**
		☐☐/☐☐/☐☐☐☐
**Office use only, data entry completed by:**		
**Signed:**	**Print:**	**Date:**
		☐☐/☐☐/☐☐☐☐

For peer review only - http://bmjopen.bmj.com/site/about/guidelines.xhtml

Moorfields Eye Hospital, Case Report Form
BALK1006

Moorfields Eye Hospital NHS Foundation Trust NHS

Study No: ☐☐ - ☐☐☐

HERMES Study

APPENDIX 1 – PROTOCOL DEVIATION

ALL FIELDS ARE MANDATORY	
**Deviation Details**	
Deviation date	*(dd mm yyyy)* ☐☐ / ☐☐ / ☐☐☐☐
Type of deviation	
Any outcomes or actions	
Deviation date	*(dd mm yyyy)* ☐☐ / ☐☐ / ☐☐☐☐
Type of deviation	
Any outcomes or actions	
Deviation date	*(dd mm yyyy)* ☐☐ / ☐☐ / ☐☐☐☐
Type of deviation	
Any outcomes or actions	
Deviation date	*(dd mm yyyy)* ☐☐ / ☐☐ / ☐☐☐☐
Type of deviation	
Any outcomes or actions	

For peer review only

I have completed this form in full and take full responsibility for any missing data.

**Signed:**	**Print:**	**Date:**
		☐☐ / ☐☐ / ☐☐☐☐
**Office use only, data entry completed by:**		
**Signed:**	**Print:**	**Date:**
		☐☐ / ☐☐ / ☐☐☐☐

For peer review only - http://bmjopen.bmj.com/site/about/guidelines.xhtml

Study No: ☐☐ - ☐☐☐

HERMES Study

APPENDIX 2 – EARLY STUDY WITHDRAWAL

ALL FIELDS ARE MANDATORY	
**Withdrawal Details**	
Did the patients discontinue the trial prematurely for reasons other than being referred back to hospital care?	Yes ☐ No ☐
Date of premature Study Discontinuation	*(dd mm yyyy)* ☐☐ / ☐☐ / ☐☐☐☐
Primary reason for discontinuation *(tick one box only)*	Patient withdraws consent ☐<br>*If known, state reason:*<br>Patient is non-compliant ☐<br>Patient is lost to follow up ☐<br>Investigator feels that it is in the patient's best interest due to adverse event ☐<br>*Related AE No:*<br>Other reason for discontinuation ☐<br>*If Other specify:*
Does the patient still agree to have their data collected and analysed as part of intent to treat analysis?	Yes ☐ No ☐

I have completed this form in full and take full responsibility for any missing data.

Signed:	Print:	Date:
		☐☐ / ☐☐ / ☐☐☐☐
**Office use only, data entry completed by:**		
**Signed:**	**Print:**	**Date:**
		☐☐ / ☐☐ / ☐☐☐☐

For peer review only - http://bmjopen.bmj.com/site/about/guidelines.xhtml

Appendix 6. Trial Documents and Subject Records

6.1 CRFs and Source Document Identification

We will establish a hub and spoke structure, where each community optometry practice liaises with its local hospital site (Moorfields Eye Hospital NHS Foundation Trust, Birmingham University Hospitals NHS Foundation Trust, Central Middlesex Hospital at London North West University Healthcare NHS Trust, and North West Anglia NHS Foundation Trust) for the day-to-day operation of the trial, through the site coordinator located at each site. 8-10 optometry practices will be located in the catchment area of Moorfields Eye Hospital NHS Foundation Trust (4-5 control and 4-5 intervention), 4-6 in the catchment area of North West London NHS Foundation Trust (Central Middlesex Hospital) (2-3 control and 2-3 intervention), 4-6 in the catchment area of North West Anglia NHS Foundation Trust (2-3 control and 2-3 intervention) and 4-6 in the catchment area of Birmingham University Hospitals NHS Foundation Trust (2-3 control and 2-3 intervention). All sites, including community optometry and hospital sites transfer data (OCT and clinical data) to Moorfields Reading Centre. The digital referral platform will be used in the 12 intervention optometry practices and the 4 HES; OCTs and clinical data from patients in the intervention optometry practices will be transferred to HES via a digital referral platform for remote review ('tele-HES') by local human experts.

Both the control arm and interventional arm will use the trial database to complete the eCRF and securely upload OCT scans. The interventional arm also transfers OCT's to the patient's hub hospital via a secure tele-ophthalmology platform. The scans and data will then be matched with the relevant trial data in the eCRF database.

For the AI Diagnostic Accuracy study, the pseudonymised OCT scans will be securely transmitted from the Moorfields Reading Centre to a secure Google Cloud Healthcare DICOM store over an encrypted connection, where it will be analysed by the DeepMind algorithm. Results from this analysis will be logged in the eCRF database. The study's use of cloud computing infrastructure adheres to January 2018 guidance from NHS Digital regarding cloud computing for health and social care. All data will be handled in accordance with the Data Protection Act 2018.

6.2 Confidentiality of Trial Documents and Subject Records

Identifiable patient data will not be accessed outside the care team without prior consent at any stage of the project. The OCT scans will be pseudoanonymised and no personal data will be included on the scans. No personal identifiers such as the patients name will be sent to the Sponsor and a unique identification code will be assigned to each OCT scan. This log of subject codes will be kept at each research site but not shared with the Sponsor.

The eCRF will not bear the subject's name or other personal identifiable data. A trial number will be used for identification on the CRFs. A separate log file which links the study ID and the patient's details, screening log and recruitment information will be kept on a protected NHS computer at hub sites. The key log will be kept at the recruitment site and will not be shared with the Sponsor. It will be

the responsibility of the chief investigator or delegated trial member to ensure the accuracy of all data recorded on the CRFs. CRFs will be completed and signed off by the Chief Investigator or delegated/authorised individual as outlined in the delegation log, the completed CRFs will be checked for accuracy and completion by the trial co-ordinator prior to data entry.

6.3 Procedures for validation and securing of electronic clinical data systems

The eCRF will be developed by the Moorfields Eye Hospital database development team. The front end will use a bespoke web application and the back end (data storage) will be hosted on Moorfields Eye Hospital Research Database SQL servers. All servers are backed up daily and with multiple restore points every day and backup copies exist in more than one All MEH clinical trial databases are part of the MEH disaster recovery strategy and have a 5 day Recovery Time Objective.

6.4 Data handling and record keeping

With respect to data handling, the senior data manager in Moorfields Eye Hospital CRF will independently ask the IT applications team to run missing data query and perform range check, logic check and data quality checks of the Electronic Database on a monthly basis.
Data queries will be sent to trial co-ordinators for clarification and confirmation whenever picked up. After all data queries are resolved and all errors are corrected, the database will then be locked with the agreement of King's CTU statistician and data will be exported by the applications manager and sent to trial statistician for data analysis. Pre-existing mechanisms for data transfer between Moorfields Eye Hospital CRF and King's CTU will be utilised.

Active project data is stored in the dedicated secure Reading Centre drive with appropriate back up arrangements. Access to the drive is restricted only to Reading Centre staff with permission and access will be monitored, granted, revoked on a per user basis. This means that only individuals with prior authorisation can access the data

Appendix 7. References

1 Kortuem K, Fasler K, Charnley A, et al. Implementation of medical retina virtual clinics in a tertiary eye care referral centre. *British Journal of Ophthalmology* 2018;102(10):1391-1395.

2 Agresti A and Coull B. Approximate is Better than “Exact” for Interval Estimation of Binomial Proportions. *The American Statistician* 1998;52(2):119-126.

3 Day A, Burr J, Bunce C, et al. Randomised, single-masked non-inferiority trial of femtosecond laser-assisted versus manual phacoemulsification cataract surgery for adults with visually significant cataract: the FACT trial protocol. *BMJ Open* 2015;5(11):p.e010381.

4 Theodossiades J, Murdoch I, and Cousens S. Glaucoma case finding: a cluster-randomised intervention trial. *Eye* 2004;18(5):483-490.

5 De Fauw J, Ledsam J, Romera-Paredes B, et al. Clinically applicable deep learning for diagnosis and referral in retinal disease. *Nature Medicine* 2018;24(9):1342-1350.

BMJ Open

Tele-ophthalmology-enabled and Artificial Intelligence-ready referral pathway for community optometry referrals of retinal disease (HERMES): A Cluster Randomised Superiority Trial with a linked Diagnostic Accuracy Study HERMES Study Report 1: Study Protocol

Journal:	*BMJ Open*
Manuscript ID	bmjopen-2021-055845.R1
Article Type:	Protocol
Date Submitted by the Author:	26-Nov-2021
Complete List of Authors:	Han, Ji Eun; University of Birmingham Institute of Inflammation and Ageing,<br>Liu, Xiaoxuan; University Hospitals Birmingham NHS Foundation Trust,<br>Bunce, Catey; Royal Marsden Hospital NHS Trust, RM CTU<br>Douiri, Abdel; King's College London, School of Population Health & Environmental Sciences<br>Vale, Luke; Newcastle University, Health Economics Group, Institute of Health and Society<br>Blandford, Ann; University College London, UCLIC<br>Lawrenson, John; City University of London, Dvision of Optometry and Visual Science<br>Hussain, Rima; Moorfields Eye Hospital NHS Foundation Trust, NIHR Biomedical Research Centre; UCL, Institute of Ophthalmology<br>Grimaldi, Gabriela; Moorfields Eye Hospital NHS Foundation Trust, NIHR Biomedical Research Centre; UCL, Institute of Ophthalmology<br>Learoyd, Annastazia; King's College London, School of Population Health & Environmental Sciences<br>Kernohan, Ashleigh; Newcastle University, Population Health Sciences Institute<br>Dinah, Christiana; London North West Healthcare NHS Trust, Ophthalmology<br>Minos, Evangelos; North West Anglia NHS Foundation Trust<br>Sim, Dawn; Moorfields Eye Hospital NHS Foundation Trust,<br>Aslam, Tariq; University of Manchester,<br>Patel, Praveen; Moorfields Eye Hospital NHS Foundation Trust, NIHR Biomedical Research Centre; UCL, Institute of Ophthalmology<br>Denniston, Alastair; University Hospitals Birmingham NHS Foundation Trust<br>Keane, Pearse; Moorfields Eye Hospital NHS Foundation Trust; University College London, Institute of Ophthalmology<br>Balaskas, Konstantinos; Moorfields Eye Hospital NHS Foundation Trust, Moorfields Ophthalmic Reading Centre and Artificial Intelligence Lab; University College London, Institute of Ophthalmology
**Primary Subject Heading**:	Ophthalmology

Secondary Subject Heading:	Health services research, Health informatics, Health economics, Evidence based practice
Keywords:	OPHTHALMOLOGY, Medical retina < OPHTHALMOLOGY, HEALTH SERVICES ADMINISTRATION & MANAGEMENT, HEALTH ECONOMICS, Telemedicine < BIOTECHNOLOGY & BIOINFORMATICS

Tele-ophthalmology-enabled and Artificial Intelligence-ready referral pathway for community optometry referrals of retinal disease (HERMES): A Cluster Randomised Superiority Trial with a linked Diagnostic Accuracy Study

HERMES Study Report 1: Study Protocol

For peer review only

Authors
Ji Eun Diana Han, Xiaoxuan Liu, Catey Bunce, Abdel Douiri, Luke Vale, Ann Blandford, John Lawrenson, Rima Hussain, Gabriela Grimaldi, Annastazia E Learoyd, Ashleigh Kernohan, Christiana Dinah, Evangelos Minos, Dawn Sim, Tariq Aslam, Praveen J Patel, Alastair K Denniston, Pearse A Keane, Konstantinos Balaskas

Affiliations
NIHR Biomedical Research Centre at Moorfields Eye Hospital NHS Foundation Trust and UCL Institute of Ophthalmology, London. (Pearse A Keane, Praveen J Patel, Dawn Sim, Rima Hussain, Gabriela Grimaldi)
University College London (Pearse Keane, Ann Blandford, Gabriela Grimaldi)
King's College London (Abdel Douiri, Annastazia E Learoyd)
City, University of London (John Lawrenson)
University of Newcastle (Luke Vale, Ashleigh Kernohan)
Manchester University NHS Foundation Trust (Tariq Aslam)
University Hospitals Birmingham NHS Foundation Trust (Alastair K Denniston, Ji Eun Diana Han, Xiaoxuan Liu)
Central Middlesex Hospital, London North West University Healthcare NHS Trust (Christiana Dinah)
North West Anglia NHS Foundation Trust (Evangelos Minos)
Royal Marsden NHS Foundation Trust (Catey Bunce)

Corresponding Author:
Konstantinos Balaskas
Moorfields Ophthalmic Reading Centre and Clinical Artificial Intelligence Lab, NIHR Biomedical Research Centre at Moorfields Eye Hospital NHS Foundation Trust and UCL Institute of Ophthalmology
162 City Road
London
EC1V 2PD
k.balaskas@nhs.net

Keywords: Telemedicine, tele-ophthalmology, artificial intelligence, machine learning, ophthalmology, retinal disease, randomised controlled trial, diagnostic accuracy

For peer review only

ABSTRACT

Introduction

Recent years have witnessed an upsurge of demand in eye care services in the UK. With a large proportion of patients referred to Hospital Eye Services (HES) for diagnostics and disease management, the referral process results in unnecessary referrals from erroneous diagnoses, and delays in access to appropriate treatment. A potential solution is a tele-ophthalmology digital referral pathway linking community optometry and HES.

Methods and analysis

The HERMES study is a cluster randomised clinical trial (C-RCT) for evaluating the effectiveness of a tele-ophthalmology referral pathway between community optometry and HES for retinal diseases. Nested within HERMES is a diagnostic accuracy study, which assesses the accuracy of an artificial intelligence (AI) decision support system (DSS) for automated diagnosis and referral recommendation. A post-implementation, observational sub-study, a within-trial economic evaluation and discrete choice experiment will assess the feasibility of implementation of both digital technologies within a real-life setting. Patients with a suspicion of retinal disease, undergoing eye examination and optical coherence tomography (OCT) scans, will be recruited across 24 optometry practices in the UK. Optometry practices will be randomised to standard care or tele-ophthalmology. The primary outcome is the proportion of false positive referrals (unnecessary HES visits) in the current referral pathway compared to the tele-ophthalmology referral pathway. OCT scans will be interpreted by the AI DSS which provides a diagnosis and referral decision and the primary outcome for the AI diagnostic study is diagnostic accuracy of the referral decision made by the Moorfields-DeepMind AI system. Secondary outcomes relate to inappropriate referral rate, cost-effectiveness analyses and human-computer interaction analyses.

Ethics and dissemination

Ethical approval was obtained from the London - Bromley Research Ethics Committee (REC 20/LO/1299). Findings will be reported through academic journals in ophthalmology, health services research and HCI.

Trial registration number: ISRCTN18106677 (Protocol version 1.1)

STRENGTHS AND LIMITATIONS OF THIS STUDY

- The HERMES study is a prospective, multi-centre implementation science study assessing clinical utility, cost effectiveness and human-computer interaction of Tele-Medicine and Artificial Intelligence Decision Support Systems in eye care referral pathways.
- The HERMES study incorporates three intertwined implementation science designs for digital eye care: an interventional, cluster randomised controlled trial of tele-medicine, an observational post-implementation study of tele-medicine and a prospective diagnostic accuracy study of Artificial Intelligence decision support systems.
- The study includes an embedded comprehensive economic evaluation, coupled with a Human-Computer Interaction analysis, generating evidence of enablers and barriers to real-life adoption of digital pathways.
- One limitation of the study is that the assessed care pathways pertain to the UK healthcare setting and may not be directly generalisable to other health systems, although reflecting a global trend towards digital transformation of healthcare.

For peer review only

BACKGROUND

Ophthalmology outpatient attendances account for 10% of all outpatient activity in the United Kingdom (UK), more than any other individual medical specialty.[1, 2] Modern ophthalmic practice in the UK is faced by the challenges of an ageing population, increasing prevalence of degenerative disease, and emergent treatments that are revolutionary but dependent on timely diagnosis. This represents a huge strain on diagnostic services and adversely impacts on timely access to care. Concurrently, there have been exponential increases in computing power and artificial intelligence, expansions in the strength and ubiquity of communications technologies, and developments in imaging capabilities, including in the community optometry setting.[3]

In the UK, primary care for ophthalmology is delivered by community-based optometry practices (High Street opticians). A large proportion of patients diagnosed with a suspicion of retinal disease, including common conditions such as neovascular ('wet') age-related macular degeneration are referred to Hospital Eye Services (HES) for diagnostics and disease management.[4, 5] The referral process results in unnecessary referrals (which can cause inconvenience and distress for patients), erroneous diagnoses, misclassification in terms of urgency, duplication of imaging tests and delays in access to treatment.

An increase of 30% in eye clinic attendances has been observed within the last 5 years throughout the UK.[6] Further increases are likely because of the increasing availability of imaging technology, and especially Optical Coherence Tomography (OCT), which is becoming ubiquitous in community optometry practices.[7] OCT is a non-invasive imaging modality that uses light to generate micrometre-resolution three-dimensional images of the retina and provides the best way to diagnose a number of common retinal pathologies including wet AMD.

This study focuses on two potentially complementary digital technologies that have the potential to revolutionise the interface between community optometrists and hospital-based eye clinics: the tele-ophthalmology platform and the Moorfields-DeepMind Artificial Intelligence Decision Support System (DSS). The technologies will be assessed through two complementary and linked quantitative studies:

1. Cluster Superiority Randomised Trial (RCT) of tele-ophthalmology
2. Prospective diagnostic accuracy study of Artificial Intelligence (Machine Learning) Diagnosis Support (the Moorfields-DeepMind algorithm)

1. Cluster Superiority Randomised Trial (RCT) of Tele-ophthalmology Pathway

Tele-medicine in Ophthalmology can help face this challenge of provision of optimal and expert care to people attending for routine eye tests in community optometry practices, through a digital referral pathway relying on a tele-ophthalmology link between community optometry and HES. This could optimise the referral process by allowing remote review of imaging and clinical data captured at the community level, by human experts based in HES. We will perform a cluster randomised superiority trial to assess the impact on service delivery metrics (such as proportion of unnecessary referrals and time from referral to treatment for urgent maculopathies) of a digital link between community

For peer review only

optometry practices and HES using a tele-ophthalmology platform. We will use a device-agnostic, tele-ophthalmology platform to enable a digital referral pathway of patients with a suspicion of retinal disease. The pilot data produced by our research team has demonstrated the potential of tele-ophthalmology to drastically improve the efficiency of the referral pathway between community optometry and HES while reducing unnecessary referrals to HES[8] and hence we propose a randomised trial powered to demonstrate superiority of the digital referral pathway against standard care.

2. Diagnostic Accuracy Study of Artificial Intelligence Diagnosis Support System

Artificial Intelligence Decision Support Systems (AI DSS) have recently been developed and shown to have good diagnostic accuracy against human experts in interpreting ocular imaging tests, such as OCT scan.[9] The collaboration between Moorfields Eye Hospital and Google DeepMind produced the arguably most advanced Deep Learning decision support system in Ophthalmology, capable of interpreting OCT scans, providing diagnosis for retinal disease and suggesting urgency of referral. In silico analysis using retrospectively collected data has validated the tool against human experts for the diagnosis of retinal disease and referral recommendations and it has been shown to be non-inferior.[9] The algorithm is uniquely and independently used in Moorfields Eye Hospital for research purposes. Thus, while such work has demonstrated promising results, a prospective study is required to demonstrate its value in practice.

We will perform a prospective study to assess diagnostic (referral) accuracy of the Moorfields-DeepMind AI DSS when applied on the OCT scans collected in the context of the tele-ophthalmology RCT. This will allow maximum utilisation of collected data from the trial and will provide estimates and confidence intervals of diagnostic (referral) accuracy. All cases included in the RCT will be reviewed by the Moorfields-DeepMind AI DSS within 48 hours of obtaining the OCT scans and a referral decision (refer routinely, refer urgently, don't refer) will be made by the algorithm for each case and recorded. The referral decisions made by the AI DSS will not be implemented in practice, yet data of the time required to obtain these decisions and any technical issues encountered with its use will be captured. These data will be incorporated into the implementation science models, including Human-Computer Interaction analysis, value-based economic evaluation, and discrete choice experiment, to identify the potential opportunities and gaps in advancing the adoption of the Moorfields-DeepMind AI DSS.

3. Impact of the Covid-19 pandemic: Post-implementation Observational Pragmatic Sub-Study

NHS services underwent rapid and significant adjustments across the board in response to extreme challenges presented by the Covid-19 pandemic. Changes to healthcare services driven by necessity are not always underpinned by a robust evidence base for efficiency and safety. In Ophthalmology, as a response to the need for social distancing and minimising unnecessary hospital visits, tele-ophthalmology pathways were commissioned recently in some areas of England using digital link to facilitate referrals between community optometry and Hospital Eye Services. Greater Manchester was an early adopter of this approach and a majority of the optometry practices in that area are now referring to NHS eye units via a tele-ophthalmology link. This local change in standard care provides a

unique opportunity to examine whether tele-ophthalmology works under usual conditions within the NHS. This sub-study will allow us to record and measure variation in quality of health care within a local region to inform our inferences from the RCT on how the tele-ophthalmology pathway will perform within a real-life setting. We will thus perform a pragmatic, observational, post-implementation study involving community optometry practices in the Greater Manchester area. This will also serve as a safety analysis allowing us to identify potential safety signals of the tele-ophthalmology pathways and adding granularity to the economic and qualitative evaluations of the RCT.

Trial aims and objectives

There are two complementary aims (aims 1&2) pertaining to the linked quantitative studies assessing the two digital technologies ('tele-ophthalmology' and the 'Moorfields-DeepMind' AI). The qualitative research element (aim 3) using Human-Computer Interaction (HCI) methodology will run across both studies to provide evidence on implementation **(Box 1)**.

For peer review only

Box 1. Aims and objectives of the HERMES study

Aims	Objectives
1. To assess the effectiveness and efficiency of a digital referral pathway between community optometry and Hospital Eye Services for referral of retinal disease enabled by a device-agnostic, tele-ophthalmology platform (superiority C-RCT).	1. Primary Objective: To compare the proportion of referrals classified as unnecessary (cases that can be safely managed without a HES consultation) between current standard care and tele-ophthalmology digital referral pathway.<br>2. Secondary Objectives:<br>● To estimate the relative efficiency of the tele-ophthalmology digital pathway compared with standard care in both within trial-based evaluation.<br>● To compare the rate of inappropriate referrals (defined as wrong diagnosis or wrong level of urgency) between standard care and the tele-ophthalmology digital pathway.<br>● To capture the number of uncommon/complex retinal referrals to secondary care and the proportion that can be safely triaged through the tele-ophthalmology platform.<br>● To compare time from referral to review and/or treatment in HES for urgent referrals (such as Wet AMD and Retinal Vein Occlusions) between standard care and tele-ophthalmology digital pathway.<br>● To assess the number of false negatives (number of patients that would have benefited from a HES consultation but were deemed suitable for continued care in the community) (Safety assessment)
2. To estimate the diagnostic (referral) accuracy and assess the 'real-life' performance of an Artificial Intelligence Decision Support System (the Moorfields-DeepMind AI) in the context of referral pathways between community optometry and HES.	1. To estimate the diagnostic (referral) accuracy of the Moorfields-DeepMind AI for recommending referral to HES from community optometry practices.<br>2. To estimate the diagnostic accuracy of the Moorfields-DeepMind AI for the diagnosis of retinal disease.<br>3. To assess the cost-effectiveness of the introduction of the DeepMind algorithm in the referral pathway between community optometry and HES.<br>4. To assess the technical feasibility of utilising the Moorfields-DeepMind AI for real-time analysis of retinal OCT scan images.<br>5. To assess real-time operational performance of the Moorfields-DeepMind AI in the tele-ophthalmology referral pathway.
3. To assess patient and healthcare professional acceptability as well as the barriers and enablers for the adoption of these digital technologies in the context of referral pathways between community optometry and HES through a Human-Computer Interaction approach.	1. To understand current workflows and practices of staff and patients in community optometry and HES so as to identify key user requirements for tele-ophthalmology tools from the perspectives of both practitioners and patients (working with care settings with diverse established practices).<br>2. To oversee the deployment of a digital referral platform at selected participating sites to ensure acceptability and acceptance by all user groups, and to understand the adoption process.<br>3. To identify factors that shape professionals' and patients' attitudes to, and trust in, the Moorfields-DeepMind AI, and how

	to present information in ways that instil appropriate confidence.<br>4. To observe workflows and practices of staff and patients in community optometry practices and HES with already established tele-ophthalmology pathways, aiding identification of technical, logistical and human factors affecting implementation of tele-ophthalmology in real-life (pragmatic sub-study).
4. To estimate the effectiveness and efficiency of a digital referral pathway between community optometry (High Street Opticians) and the Hospital Eyes Services for referral of retinal diseases enabled by a tele-ophthalmology platform in a real-life, observational post-implementation sub-study.	1. To compare the proportion of referrals classified as unnecessary (cases that can be safely managed without a HES consultation) against Reference Standard and the intervention arm of the RCT.<br>2. To compare the rate of inappropriate referrals (defined as wrong diagnosis or wrong level of urgency) against the Reference Standard and the intervention arm of the RCT.<br>3. To assess the number of false negatives (number of patients that would have benefitted from a HES consultation but were deemed suitable to continued care in the community) (Safety assessment).<br>4. To compare time from referral to review and/or treatment in HES for urgent referrals (such as Wet AMD and Retinal Vein Occlusions) between post-implementation real-life tele-ophthalmology digital pathway and the intervention arm of the RCT.<br>5. To estimate the relative efficiency of the real-life tele-ophthalmology digital pathway compared with the RCT tele-ophthalmology pathway.

For peer review only

METHODS AND ANALYSIS

Study Design

Superiority C-RCT of Tele-Ophthalmology Pathway

An interventional superiority cluster randomised trial (RCT) will be performed comparing standard practice for referral of suspicious retinal disease with tele-ophthalmology between community optometry and HES. This part of the study will be reported according to the CONSORT extension for C-RCTs.[10]

Diagnostic Accuracy Study of Artificial Intelligence Diagnosis Support System

A prospective study will be conducted using the data of the above RCT to assess the diagnostic (referral) accuracy of an advanced AI DSS (the Moorfields-DeepMind Algorithm) for the automated diagnosis and referral recommendation for retinal disease. OCT scans transferred to the Moorfields Reading Centre in the course of the study will be assessed by the DeepMind algorithm in 'real-time' and its referral recommendations will be recorded and analysed for diagnostic (referral) accuracy and compared against the performance of human experts in the standard care and tele-ophthalmology arms of the RCT. This part of the study will be reported according to the STARD 2015 statement,[11] or STARD-AI.[12] A within-trial based economic evaluation will estimate the efficiency of alternative referral models for retinal disease. A Human-Computer Interaction (HCI) analysis using qualitative methods will assess feasibility of implementation of both digital technologies.

Observational, post-implementations, pragmatic sub-study

Community Optometry practices in the Greater Manchester area will continue to refer patients with suspicious retinal disease to HES using the locally established tele-ophthalmology digital pathway. Referral recommendations will be compared against a reference standard provided by Moorfields Reading Centre to inform the assessment of real-life effectiveness and efficiency of the tele-ophthalmology referral pathway.

Setting

For the C-RCT and diagnostic accuracy study, patients will be recruited at 24 optometry practices (clusters) in the catchment areas of 4 HES sites: Moorfields Eye Hospital NHS Foundation Trust (8-10 practices), Birmingham University Hospitals NHS Foundation Trust (4-6 practices), Central Middlesex Hospital at London North West University Healthcare NHS Trust (4-6 practices) and North West Anglia NHS Foundation Trust (4-6 practices). 12 clusters (each cluster is an optometry practice) will be randomised to standard care and 12 clusters to the intervention (tele-ophthalmology). This selection of sites includes urban, sub-urban and rural locations within the UK allowing the inferences made from this study to be applicable to more of the UK population. 2 additional optometry practices (clusters) will be randomised (1:1) in a reserve capacity in case of a cluster drop-out or in order to accelerate the recruitment process. (**Appendix 1**)

For peer review only

For the pragmatic, observational, post-implementation sub-study, patients will be recruited at 12 optometry practices (clusters) in the catchment area of Manchester University NHS Foundation Trust. These practices have adopted a tele-ophthalmology referral pathway as standard practices.

The enrollment start date is September 2020 with anticipated primary completion date in August 2023. Eligible practices need to have OCT devices and the activity volume and track record of referral to HES that will allow achieving the per practice recruitment target.

All OCT scans and clinical vignettes from each case will be transferred to the Moorfields Reading Centre that will provide the reference standard (diagnosis and referral recommendation). All suitable OCT scans will be processed by the DeepMind algorithm at the Moorfields Reading Centre in 'real-time' for the AI diagnostic accuracy study.

Participants

Adults (≥18 years) attending for an eye examination at the participating community optometry practices who undergo an OCT scan will be considered for participation in the study. Only people with a suspicion of retinal disease in the opinion of the community optometrist will be recruited in the RCT and diagnostic accuracy study. As entire optometry practices (clusters) will be randomised into standard care or tele-ophthalmology, patients who are approached and agree to take part in the study will consent **(Appendix 2)** to data collection and analysis - there will not be patient-level randomisation. The patient-level inclusion criteria are outlined in **Box 2.**

Box 2. Patient-level selection criteria

Inclusion Criteria

- Able to give consent and understand the study
- Able to cooperate by following study specific instructions
- Adults (≥18 years) attending the involved community optometry practices who underwent an OCT scan
- Individuals who at the opinion of the community optometrist have any suspicion of a retinal condition (including atrophic ("dry") AMD, wet AMD, diabetic retinopathy, macular oedema, macular holes, epiretinal membranes, central serous chorio-retinopathy, genetic eye disease)
- Macular OCT scan performed at community optometry

Exclusion Criteria

- Individuals with any non-retinal ocular co-morbidities in either eye other than cataract
- Individuals with media opacities, inability to position or fixate or any other reason that prevents acquisition of good quality OCT scans (at the discretion of the community optometrist)

Allocation to trial groups

Simple randomisation will be performed for involved optometry practices into the intervention and control arms. Randomisation will be performed with the unit of allocation being the cluster rather than the individual and allocation concealment will be at the cluster level. Optometry practices will be randomised 1:1 to standard care or tele-ophthalmology stratified by the hospital site. Optometry practices are committed to the allocated study arm for the duration of the recruitment period or until they have recruited the minimum of the per cluster recruitment range (10 patients).

Superiority C-RCT of Tele-Ophthalmology Pathway

Community optometry practices will be randomised to either continue with standard care for referral of retinal disease to hospital-based eye clinics or move to the tele-ophthalmology digital referral pathway (Figure 1).

Intervention arm for Superiority C-RCT: Digital Pathway The intervention pathway is the tele-ophthalmology model for referral of patients with suspicion of retinal disease from community optometry to HES using a digital referral platform. Patients who attend at participating community optometry practices will undergo a clinical assessment and OCT scan.

Figure 1. Superiority cluster randomised trial arms.

Participants' OCT scan and clinical information will be transferred via the digital referral platform to corresponding HES. In each case, human expert clinicians based in HES (ophthalmologists or specialist optometrists with a minimum of two years' experience of independent practice in the context of HES retinal clinics) will make a referral decision remotely ('tele-HES') after review of OCT scan and clinical information on the digital referral platform. A referral recommendation by the community optometrist will also be recorded but not acted upon, to measure the proportion of correct/incorrect referrals in each arm. **(Appendix 3)**.

The remote review of OCT scans and clinical data at 'tele-HES' will be performed by expert clinicians (medics or specialist optometrists) experienced in retinal clinics (minimum of two years' experience of independent practice in the context of retinal clinics in HES) based at Moorfields Eye Hospital, Central Middlesex Hospital, North West Anglia NHS Foundation Trust Hospitals or Queen Elizabeth Hospital Birmingham with access to senior advice by Consultant Ophthalmologists specialising in retinal disease.

Control arm for Superiority C-RCT: Standard Practice

The control pathway is standard practice for referral of patients with suspicion of retinal disease from community optometry to HES. Patients who attend a participating community optometry practice will undergo a clinical assessment and OCT scan. Patients with a suspicion of any retinal disease in the opinion of the community optometrist will be included in the study and will receive a referral decision (refer urgently to HES; refer routinely to HES; don't refer to HES) by the community optometrist. All OCT scans and a clinical vignette from each case will be transferred to the Moorfields Reading Centre that will provide the reference standard for referral recommendations.

For peer review only

Diagnostic Accuracy Study

All referred and non-referred cases (from the standard care and tele-ophthalmology arm above; **Figure 2**) will be included in the AI diagnostic study. All suitable OCT scans will be transferred prospectively on a weekly basis to the Moorfields Ophthalmic Reading Centre. OCT scans will be processed by the Moorfields-DeepMind AI system and end-to-end timing of the process will be captured for each case. For each case the Moorfields-DeepMind AI will provide a:

1. Diagnosis
2. Decision to refer or not
3. Urgency of referral (routine or urgent)

Figure 2. Diagnostic accuracy study arms.

Safety Net

In cases where 'tele-HES' decision is 'don't refer', the patient will be provided with additional information and alerts for clinical symptoms that should prompt a visit directly to the A&E department of the corresponding secondary care site (Moorfields Eye Hospital, Central Middlesex Hospital, North West Anglia NHS Foundation Trust Hospitals or Queen Elizabeth Hospital Birmingham). Additionally, in cases where a disagreement is found between the decision by the community optometrist and the one made in 'tele-HES', patients will be offered a follow-up appointment at the community optometry practice within 4 weeks.

Reference Standard

OCT scans from the standard care and tele-ophthalmology arms will be transferred to the Moorfields Ophthalmic Reading Centre. The reference standard will be provided by the expert Ophthalmic Reading Centre for the C-RCT, diagnostic accuracy study, and the Pragmatic sub-study. The reference standard will be the referral decisions and disease diagnosis made at the Reading Centre on the basis of review of images and clinical history and will apply to the RCT, the AI Diagnostic Accuracy study and the Post-implementation, Pragmatic sub-study. Specifically, for each patient, the OCT scan (including b-scans and colour fundus image) and a clinical vignette including visual acuity, age, symptoms, ocular and systemic history will be reviewed by two expert graders. The process to be followed is double-grading with adjudication by a senior retinal specialist at the Moorfields Reading Centre.

For peer review only

Outcome Measures

The outcomes for the superiority C-RCT and diagnostic accuracy study are outlined in **Box 3.**

Box 3. Study outcomes

**Superiority C-RCT**	**Diagnostic Accuracy Study**	**Pragmatic Sub Study**
Primary Outcome C-RCT: Proportion of false positive referrals in the current referral pathway and the tele-ophthalmology referral pathway.	Primary outcome diagnostic accuracy study: Diagnostic accuracy of the referral decision made by the Moorfields-DeepMind AI (refer to HES, do not refer to HES) against the Reference Standard (Moorfields Reading Centre).	
Secondary outcomes C-RCT:<br>1. Proportion of wrong diagnosis and wrong referral urgency in standard and tele-ophthalmology pathways against the reference standard<br>2. Proportion of false negative referrals, as well as sensitivity and specificity in standard and tele-ophthalmology pathways against the reference standard<br>3. Time from referral to consultation for urgent and routine referrals in standard and tele-ophthalmology pathways<br>4. Time from referral to treatment for urgent maculopathies in standard and tele-ophthalmology pathways<br>5. Number of uncommon referrals (rare disease) that can be safely triaged in the tele-ophthalmology pathway<br>6. Within trial cost-effectiveness and cost-consequences of the tele-ophthalmology	Secondary outcomes diagnostic accuracy study:<br>1. Sensitivity and specificity of Moorfields-DeepMind AI for the diagnosis of retinal disease<br>2. Sensitivity and specificity of Moorfields-DeepMind AI for referral urgency<br>3. Proportion of false positive referrals in the standard and tele-ophthalmology pathways when human assessors are replaced by the AI DSS<br>4. Proportion of wrong diagnosis and wrong referral urgency in the standard and tele-ophthalmology pathways when human assessors are replaced by AI DSS<br>5. Uptime and end-to-end inference speed of technical infrastructure supporting the AI DSS<br>6. Average time of end-to-end output (referral recommendation) by the AI DSS	Secondary outcomes of pragmatic sub study:<br>1. Proportion of false positive referrals in the tele-ophthalmology referral pathway against the Reference Standard and the intervention arm in the main RCT.<br>2. Proportion of wrong diagnosis and wrong referral urgency in the tele-ophthalmology pathway compared against the Reference Standard and the intervention arm in the main RCT study<br>3. Proportion of false negative referrals compared against the Reference Standard and the intervention arm in the main RCT study<br>4. Time from referral to review and/or treatment in HES for urgent referral) in the post-implementation real-life tele-ophthalmology digital pathway

digital pathway compared with standard care<br>7. Modelled cost-consequences and net benefits of alternative diagnostic and referral strategies	7. Cost-consequences and net benefits of AI-enabled digital referral pathway	

Patient and Public Involvement

Patient and public members were consulted prior to the trial protocol design on perception of tele-ophthalmology and issues of data privacy, impersonal care, trust in the technology, confidence in the quality of care provided through digital means were explored. A patient and public involvement group based at Moorfields Eye Hospital was consulted during the trial design to advise on barriers to recruitment, issues around geographical spread of study sites and patient information material. After study commencement, the PPI group is planned to meet yearly and an end-of-study debrief is planned with all PPI contributors, which will include discussions of the prioritisation and dissemination of study results both to the public as well as relevant healthcare professionals.

For peer review only

DATA ANALYSIS PLAN

Statistical Analysis

In the Superiority C-RCT of tele-ophthalmology pathway study, the primary analysis will be conducted following an intention-to-treat principle where all randomised patients are analysed in their allocated group whether or not they receive their randomised management plan. The primary outcome is the proportion of false referrals, measured at the patient level. This will be compared between management groups using logistic regression adjusting for clustered centres. Outcomes will be reported as adjusted odds ratios.

In the AI diagnostic accuracy study, we will report estimates of sensitivity and specificity of the DeepMind algorithm for referral decisions with 95% confidence intervals. Our primary analysis will combine urgent and standard referral to HES and compare against no referral to HES but a sensitivity analysis will be conducted to evaluate urgent referrals. The full statistical consideration and analysis is provided in **Appendix 4.**

Implementation Science Study Components

Focus of Analysis

The primary focus for analysis will be on facilitators and barriers to implementation of the tele-ophthalmology system and the introduction of Artificial Intelligence Decision Support across clinical contexts, along with accounts of how it changes workflow and patient experience. Evaluation will be formative, so as to inform future implementations and also to contextualise the analyses of clinical effectiveness and cost effectiveness. For the AI DSS, questions to be included in interviews will involve whether the AI is to be used as decision aid (e.g., as a filter for disease/no disease) or as a completely independent decision making tool, issues around trust in the technology, perceptions of medicolegal concerns (who is responsible for the decisions?), the optimal place in the care pathway for positioning the AI (high street optician or hospital-based eye services or both), concerns such as de-skilling of practitioners (as diagnostic decisions may be devolved to AI), reduced employment opportunities, the need for a 'safety net'/quality check to oversee and 'sanity check' the performance of the AI system, and impersonal care for patients, and perceived benefits such as more efficient and appropriate care, greater confidence in the process, etc. We will also particularly focus on the question of ‘interpretability’ of AI DSS and the ‘black box’ phenomenon and whether it influences trust and potential uptake of this technology. The ‘interpretability’ of AI DSS is a major factor in technology uptake and may influence the direction of AI DSS developers towards more interpretable technologies.

The data collected from sites with established tele-ophthalmology pathways (Greater Manchester) will be particularly valuable for identifying barriers to implementation in a real-life context that wouldn’t be picked up in the controlled environment of the RCT such as technical, staffing, training and human factors. Such barriers can be consequential with respect to patient safety as they have the potential to lead to delays in clinical review or missed cases and therefore the post-implementation sub-study

For peer review only

offers an opportunity to explore potential safety signals of the tele-ophthalmology pathways not typically observable in the context of RCT trials.

Human-Computer Interaction (HCI) Analysis

As noted above, the aims of the HCI analyses are to assess the barriers and enablers for the adoption of the proposed digital technologies in the context of referral pathways between community optometry and HES through a Human-Computer Interaction approach.

In order to capture patient and staff perspectives of tele-ophthalmology models of care as well as AI DSS, we will take a qualitative approach, conducting interviews and observations in both community optometry and HES. A full account on sampling, recruitment and analysis methods is provided in **Appendix 1**. We will compare people's expectations (what they believe they will want and use) with their experiences when they have access to the relevant technology. In order to compare expectations against experiences, we will gather data in a variety of settings over the course of the project:

- *In the first six months* of the project, the focus will be on understanding the adoption process and factors that contribute to success in adoption. Longitudinal data will be gathered at three selected sites: two optometry practices and the Birmingham HES. Data gathering will focus on expectations and current work practices before implementation; barriers, facilitators and experiences during implementation; and perceptions and practices post-implementation.
- *Over the subsequent 12 months* (months 7-18), similar data gathering and analysis methods will be adopted in two optometry practices that are already experienced in using tele-ophthalmology (sites that have already adopted tele-ophthalmology in the Greater Manchester area); two practices that are not using tele-ophthalmology and have no immediate plans to transition (control sites for the quantitative studies described above); and a second HES (Moorfields Eye Hospital).The focus will be on understanding workflows, practices and user requirements, including facilitators and barriers to adoption; and identifying factors that shape attitudes to the AI DSS, and how to present information to instill appropriate confidence.

Economic Analysis

Cost-Consequence Analysis

The economic evaluation will comprise a within trial cost-consequence analysis (CCA) directly comparing the tele-ophthalmology pathway with the current referral pathway. This analysis highlights the choices and trade-offs between the modalities of care provision without an explicit synthesis of data into a single measure of efficiency. The results for the cost-consequence analysis will be presented as a balance sheet, which will include point estimates and appropriate measures of variance. From an NHS perspective, costs such as hospital visits, medications and community GP visits will be costed. Unit costs for resource use will be derived from published sources e.g. NHS Reference Costs and Unit Costs of Health and Social Care.[13] When considering the addition of the community optometry

For peer review only

perspective, the costs to purchase and maintain an OCT scanner will be considered. The costs of acquisition will be derived from market prices and converted into a cost per patient using standard economic methodology.[14] In addition to this, the costs of the Moorfields-DeepMind algorithm will be considered within trial analysis. We will base this cost on advice from the algorithm owners as well as consideration of analogous algorithms. We expect there to be considerable uncertainty around the price to the NHS as a market price is not available. Therefore, we will explore the impact on efficiency of a range of prices. This will help decision-makers consider the maximum price they might be willing to pay for this algorithm given the benefits it may provide. In addition to the costs of running the algorithm in terms of hardware, software and staff, required will be considered. This will be based upon its use within the study and advice from members of the study. A sensitivity analysis will be carried out to explore how the adoption of different perspectives (i.e. who is bearing the costs) will affect the cost effectiveness of the intervention. Outcomes which may be included in this CCA are; false positives, false negatives, unnecessary hospital visits and duration of the time spent with an untreated macular disorder. These will be compared to the costs of provision and of the intervention and with the results of the Discrete Choice Experiment (DCE) described below. Costs that will be included will be those that fall on the NHS and community optometry practices. Deterministic sensitivity analysis e.g. variations in unit costs, will also be conducted. The consequences for each of the comparators will be based upon a further consideration of outcomes (for example necessary referrals missed, correct referrals, individuals correctly not referred). The likelihood of these different outcomes (given as percentages) will be described.

To include insights derived from the pragmatic post-implementation study, the economic evaluation will also provide the following additional elements:

1. The Manchester sub-study group will inform estimates of the cost of the intervention, as delivered in a 'real world' application which may be more realistic than those estimated from a trial setting.
2. The Manchester sub-study group will be used to inform an exploratory analysis. In this the costs and consequences of the real life sub-study group will be compared to the results from the trial group to identify if there is any meaningful difference between the two sets of data and identify what the driving factors are. As a safety analysis will be carried out as part of a sub-study, the cost and consequences of any unexpected adverse events that are recorded will be included in the cost consequence analysis. If any safety events become apparent during the design of the Discrete Choice Experiment then these may be used as the basis of different attributes and levels in the study design.

Discrete Choice Experiment (DCE)

In addition to the CCA economic evaluation a Discrete Choice Experiment (DCE) will be carried out to assess preferences of the general public about the tele-ophthalmology pathway. A DCE is an attribute-based survey method for measuring benefits. It offers participants at least two alternative choices which vary across several attributes of interest. These can include several attributes of how the intervention is provided and its effect on health and other outcomes. Each of these attributes can vary over a range

For peer review only - http://bmjopen.bmj.com/site/about/guidelines.xhtml

of levels. The choice of DCE attributes for this intervention will be informed from existing literature on macular disease and provision of eye care service. The output of the qualitative study will also be examined for any attributes which could affect the preferences of the users of the service. The DCE will also be used to value in monetary terms the relative importance of the different consequences included in the CCA. To do this it will use methods previously successfully used in other NIHR funded studies.[15, 16] The results of this monetary value will be used to inform a further cost benefit analysis.

Cost Benefit Analysis

The results of the DCE will be used to value the outcomes described in the cost-consequence analysis. Outcomes will be expressed as a net monetary benefit by combining the differences in each outcome by the willingness to pay for a unit change in that outcome. These values are known as willingness to pay (WTP) values and will be derived from the DCE described above. Cost will be included as part of the attributes that participants choose between so that participants can express their WTP values for a described outcome. The cost of the providing the outcome will be derived from the unit costs described earlier. A net benefit in monetary terms for these outcomes will be derived be subtracting the cost of the outcome from the WTP value (including a negative net benefit).

The values for the cost attribute will be based on pilot work and reviews of prior studies in this area for example Burr et al. (2012) valued an intervention to monitor ocular hypertension to prevent glaucoma using a DCE. The range of values was between £15-70 (GBP 2012). Similarly, Shih et al. (2007) assessed the WTP for a diabetic retinopathy screening service and reported a narrower range of between $4-$24 (USD 2007).[17] After the attributes have been established then the piloting stages will occur. A survey company will be utilised to gain a large enough sample (www.researchnow.co.uk). The participants will be offered a small incentive (£1-£2) to complete the survey. The overall sample will be representative as closely as possibly for factors such as age, sex and ethnic background for the UK population. Optimal sample size requirements for the limited dependent variable models estimated in DCEs depend on knowledge of the true choice probabilities, which are not known prior to undertaking this research. However, previous DCE studies have shown that robust choice models can be estimated from sample sizes between 50-100 respondents. As such, a small pilot sample of 100 participants will be used as a sample to monitor the rate of completion and to carry out preliminary analysis and change any parts of the survey that are necessary. After the preliminary analysis is carried out, then a further sample of 300 participants will be surveyed which will be sample size comparable to other HTAs in this area. The results of the DCE will be analysed using conditional logit regression analysis, which will measure the direction and strength of the participant preferences. Sub-group analysis will also be carried out to see if factors such as age, sex or ethnic background have any effect on the resulting preferences. Probabilistic and deterministic sensitivity analyses will be carried out to vary parameter uncertainty for both the costs and the effects. Results of the probabilistic sensitivity analysis will be presented as point estimates of net-benefits, plots of costs and benefits net benefit curves, which show the likelihood of each intervention being most likely to have the highest net benefit.

For peer review only

Dissemination of Results

Findings will be reported through academic journals in ophthalmology, health services research and HCI; some will focus on findings from the HCI studies, and some will relate findings to those of the parallel studies covering other themes. PPI workshops will be organised in Birmingham and London before the main study period, with a focus on designing the study adapting the model from an earlier project (the “before” study is reported by Furniss et al., 2016).[18]

ETHICS AND DISSEMINATION

The research project will adhere to the UK Framework for Health and Social Care research. Ethics approval has been obtained for this project. No particular challenges are expected given the low risk nature of the intervention of the RCT, the safety net arrangements for cases not referred to HES from community optometry, the observational design of the AI diagnostic accuracy study, and the relatively low personal sensitivity of the topics to be investigated in the HCI studies.

With respect to confidentiality of patient records and case report forms (CRF; Appendix 5), identifiable patient data will not be accessed outside the care team without prior consent at any stage of the project. The OCT scans will be pseudoanonymised and no personal data will be included on the scans. No personal identifiers such as the patients name will be sent to the Sponsor and a unique identification code will be assigned to each OCT scan. This log of subject codes will be kept at each research site but not shared with the Sponsor. Regarding data handling, the senior data manager in Moorfields Eye Hospital CRF will independently ask the IT applications team to run missing data query and perform range check, logic check and data quality checks of the Electronic Database on a monthly basis. Active project data is stored in the dedicated secure Reading Centre drive with appropriate back up arrangements. Access to the drive is restricted only to Reading Centre staff with permission and access will be monitored, granted, revoked on a per user basis. This means that only individuals with prior authorisation can access the data**.** Further details on the handling of trial documents and subject records are found in Appendix 6.

Planned outputs:

- High-impact peer-reviewed publications in Ophthalmology and Health Service Research journals

- Presentations in conferences, including the Royal College of Ophthalmologists, the Association for Research in Vision and Ophthalmology, the American Academy of Ophthalmology conference, The College of Optometrists

- The output of this program has the potential to influence the healthcare landscape for eye care by validating digital care pathways for patients with retinal disease. The outcomes of this research will be communicated to NHS England and NHSx to inform policy on the role of digital technologies, including tele-medicine and AI DSS.

The Research team and the Sponsor will actively approach and engage key parties such as the

For peer review only

College of Optometrists, stakeholders in community optometry and Clinical Commissioning Groups.

A detailed engagement plan will be formulated to disseminate the results of this research in order to inform policy decisions for optimising patient care.

AUTHORS' CONTRIBUTIONS

Ji Eun Diana Han and Xiaoxuan Liu collected methodological data and drafted the manuscript

Catey Bunce, Abdel Douiri and Annastazia E Learoyd provided intellectual input in study methodology and statistical analysis planning and critical review of the manuscript

Luke Vale and Ashleigh Kernohan provided intellectual input in study methodology and economic evaluation planning and critical review of the manuscript

Ann Blandford provided intellectual input in study methodology and human-computer interaction analysis planning and critical review of the manuscript

John Lawrenson, Rima Hussain, Gabriela Grimaldi, Christiana Dinah, Evangelos Minos, Dawn Sim, Tariq Aslam, Praveen J Patel, Alastair K Denniston, Pearse A Keane provided intellectual input in study methodology and design, contributed to drafting and critical review of the manuscript

Konstantinos Balaskas conceived the study, initiated the project, provided intellectual input in study methodology and design and critical review of the manuscript

FUNDING

The project is funded by an NIHR Health Technology Assessment grant (HTA - Project: 18/182 HTA Digital Technologies to Improve Health and Care).

ACKNOWLEDGMENT

The HERMES Research Team members would wish to extend their gratitude to the members of the HERMES Trial Advisory Committees for their expert advice and guidance and their valuable contribution to study planning and delivery.

Trial Steering Committee:

Chair: Professor Richard Gale, York University Hospitals NHS Foundation Trust

Members:

Professor Irene Stratton, Gloucestershire Hospitals NHS Foundation Trust

Dr Bushra Mushtaq, Sandwell and West Birmingham NHS Foundation Trust

Dr Gabriella De Salvo, Southampton University Hospitals NHS Foundation Trust

Mrs Geraldine Hoad, the Macular Society

For peer review only

Data Monitoring Committee:

Chair: Dr Louise Downey, Hull University Teaching Hospitals NHS Foundation Trust

Members:

Dr Gabriela Czanner, Liverpool John Moores University

Dr Markus Groppe, Buckinghamshire Healthcare NHS Trust

Competing Interests Statement

Dr Pearse A Keane has previously been consultant for Google DeepMind

For peer review only

REFERENCES

1 The Royal College of Ophthalmologists, 2021. *The Way Forward*. [online] Rcophth.ac.uk. Available at: <https://www.rcophth.ac.uk/wp-content/uploads/2015/10/RCOphth-The-Way-Forward-Executive-Summary-300117.pdf> [Accessed 9 June 2021].

2 NHS Digital. 2020. *Hospital Outpatient Activity 2019-20: Main Specialty*. [online] Available at: <https://digital.nhs.uk/data-and-information/publications/statistical/hospital-outpatient-activity/2019-20> [Accessed 9 June 2021].

3 Dabasia P, Edgar D, Garway-Heath D, et al. A survey of current and anticipated use of standard and specialist equipment by UK optometrists. *Ophthalmic and Physiological Optics 2014*;34(5):592-613.

4 Looker H, Nyangoma S, Cromie D, et al. Rates of referable eye disease in the Scottish National Diabetic Retinopathy Screening Programme. *British Journal of Ophthalmology 2014;*98(6):790-795.

5 Konstantakopoulou E, Harper R, Edgar D, et al. Clinical safety of a minor eye conditions scheme in England delivered by community optometrists. *BMJ Open Ophthalmology* 2018;3(1):p.e000125.

6 MacEwen C. 2016. *Increasing demand on hospital eye services risks patients losing vision - The Royal College of Ophthalmologists*. [online] The Royal College of Ophthalmologists. Available at: <https://www.rcophth.ac.uk/2016/03/increasing-demand-on-hospital-eye-services-risks-patients-losing-vision/> [Accessed 9 June 2021].

7 McCormick E. 2017. *OCT rollout in every Specsavers announced*. [online] Aop.org.uk. Available at: <https://www.aop.org.uk/ot/industry/high-street/2017/05/22/oct-rollout-in-every-specsavers-announced> [Accessed 9 June 2021].

8 Kortuem K, Fasler K, Charnley A, et al. Implementation of medical retina virtual clinics in a tertiary eye care referral centre. *British Journal of Ophthalmology* 2018;102(10):1391-1395.

9 De Fauw J, Ledsam J, Romera-Paredes B, et al. Clinically applicable deep learning for diagnosis and referral in retinal disease. *Nature Medicine* 2018;24(9):1342-1350.

10 Campbell M, Piaggio G, Elbourne D, et al. Consort 2010 statement: extension to cluster randomised trials. *BMJ* 2012;345(sep04 1):pp.e5661-e5661.

11 Bossuyt P, Reitsma J, Bruns D, et al. STARD 2015: an updated list of essential items for reporting diagnostic accuracy studies. *BMJ* 2015:p.h5527.

12 Sounderajah V, Ashrafian H, Aggarwal R, et al. Developing specific reporting guidelines for diagnostic accuracy studies assessing AI interventions: The STARD-AI Steering Group. *Nature Medicine* 2020;26(6):807-808.

13 Curtis L and Burns A. *Unit Costs of Health and Social Care 2019*. Canterbury: Personal Social Services Research Unit, University of Kent 2019:p.176.

14 Drummond M. *Methods for the economic evaluation of health care programmes*. Oxford: Oxford University Press 2005.

15 Burr J, Botello-Pinzon P, Takwoingi Y, et al. Surveillance for ocular hypertension: an evidence synthesis and economic evaluation. *Health Technology Assessment* 2012;16(29).

16 McCormack K, Wake B, Perez J, et al. Laparoscopic surgery for inguinal hernia repair: systematic review of effectiveness and economic evaluation. *Health Technology Assessment* 2005;9(14).

17 Shih H, Chou P, Chen S, et al. A Community-based Study of the Willingness to Pay Associated with Screening for Diabetic Retinopathy among Type 2 Diabetes in Kinmen, Taiwan. *Journal of Epidemiology* 2007;17(6):186-193.

18 Furniss D, Iacovides I, Lyons I, et al. Patient and public involvement in patient safety research: a workshop to review patient information, minimise psychological risk and inform research. *Research Involvement and Engagement* 2016;2(1).

For peer review only

CONSORT Tele-Ophthalmology Superiority Cluster Randomised Trial

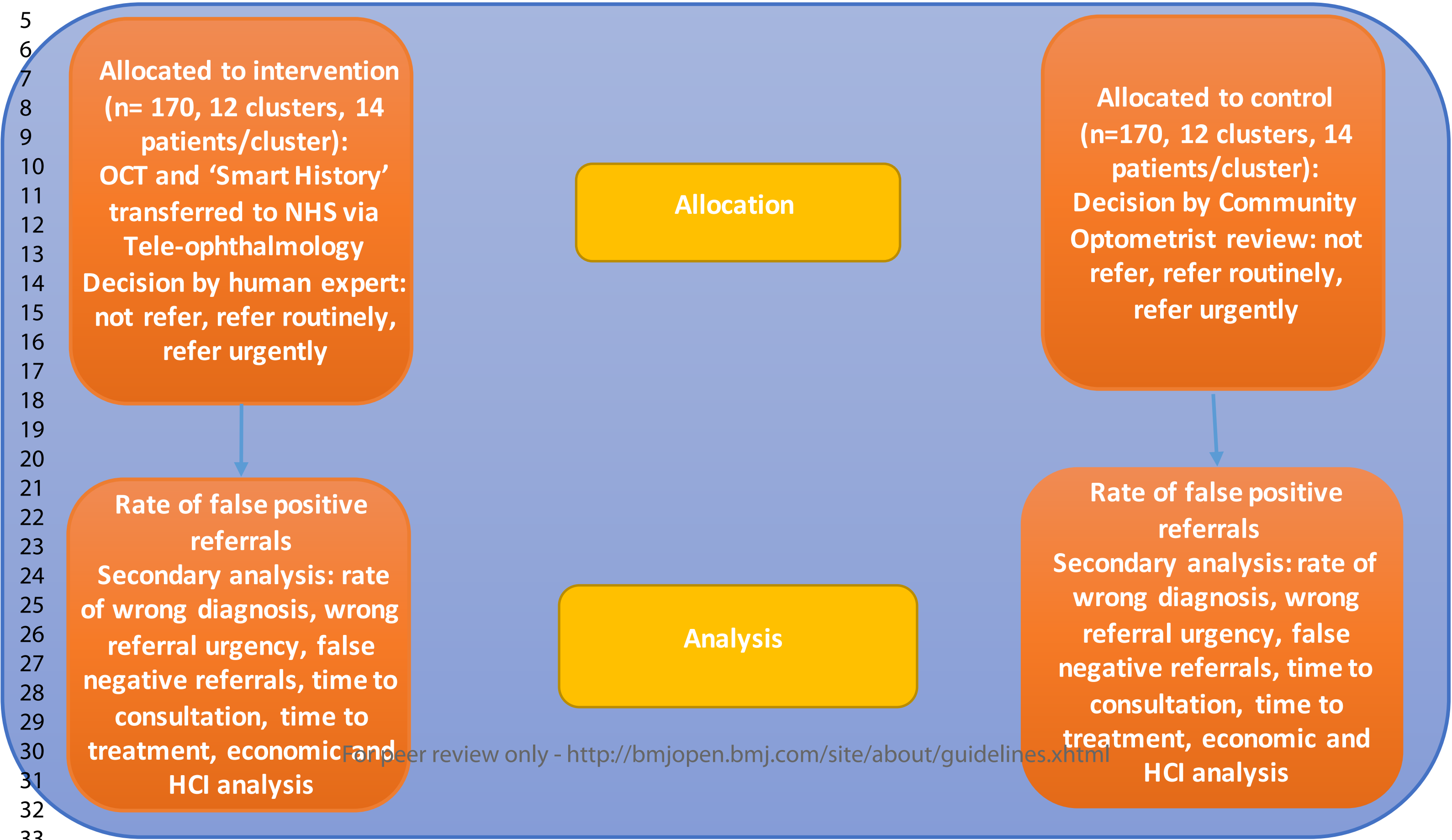

For peer review only - http://bmjopen.bmj.com/site/about/guidelines.xhtml

STARD Artificial Intelligence Observational Prospective Diagnostic Study

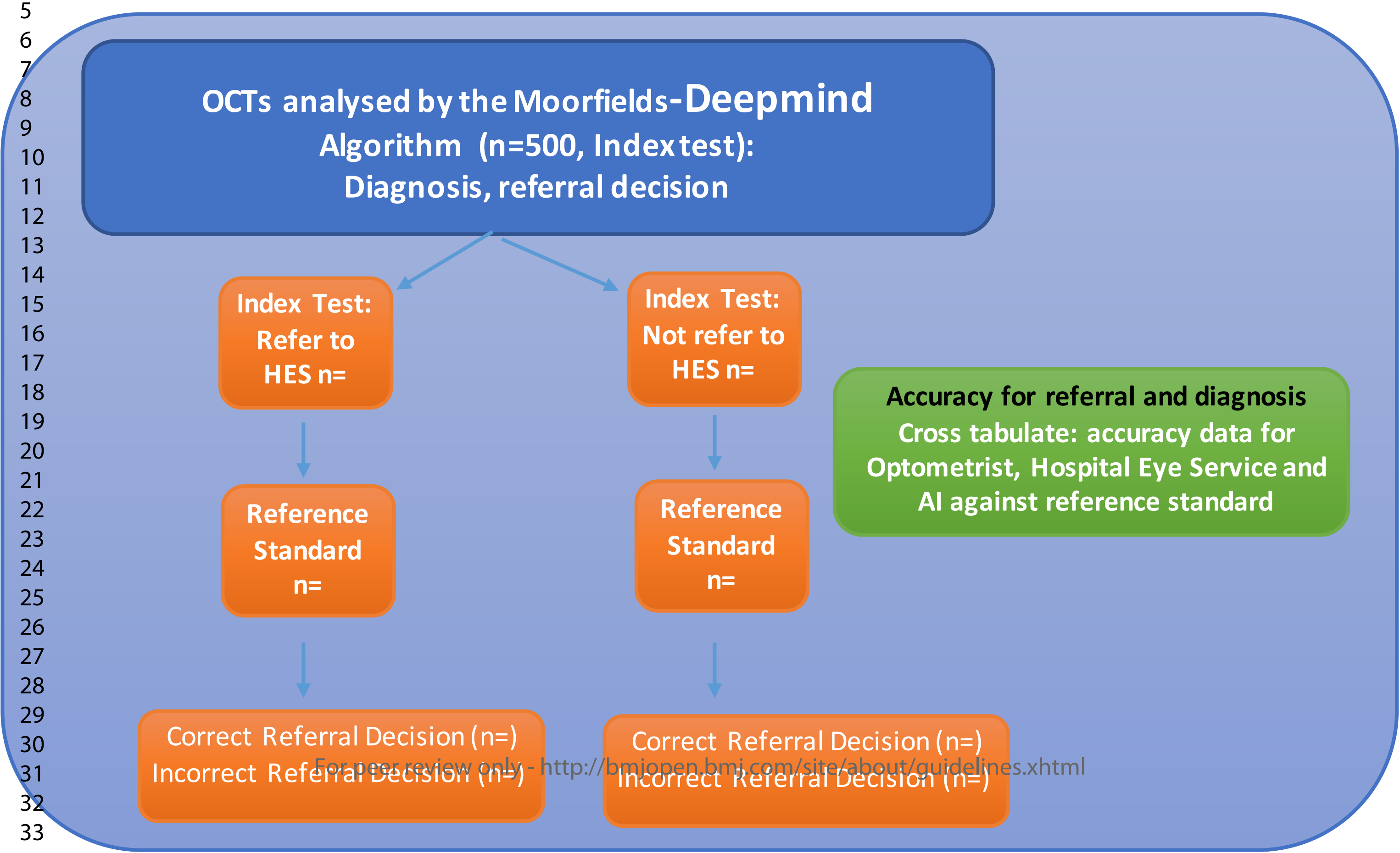

For peer review only - http://bmjopen.bmj.com/site/about/guidelines.xhtml

Appendix

Table of Contents

Appendix 1. Sampling, Recruitment and Analysis Methods
Appendix 2. Participant Information Sheet and Consent Form
Appendix 3. Digital Pathway Decisions
Appendix 4. Statistical Consideration
4.1 Sample Size Calculation
Recruitment plan
Pragmatic Sub-Study
4.2 Statistical analysis
Appendix 5. Case Report Form
Appendix 6. Trial Documents and Subject Records
6.1 CRFs and Source Document Identification
6.2 Confidentiality of Trial Documents and Subject Records
6.3 Procedures for validation and securing of electronic clinical data systems
6.4 Data handling and record keeping
Appendix 7. References

Appendix 1. Sampling, Recruitment and Analysis Methods

Purposive sampling techniques will be applied to recruit participants who are representative of all key patient and professional groups across all sites, including both “power users” and reluctant users. In each setting, the aim will be to interview 15 patients and up to 10 clinicians and other professionals (settings: secondary care clinics; pre-; and post-transitioning optometry practices), depending on the sizes of the clinics selected for inclusion. The interviews will be conducted by a Process Evaluation Specialist, they will be semi-structured and will be designed to address the research objectives outlined above. Probes such as anonymised screenshots from the digital referral platform and illustrative information presentation prototypes from the DeepMind algorithm will be used in interviews to support the exploration of the themes. Interview data will be transcribed and analysed by a qualitative methods expert using inductive Thematic Analysis, with a particular focus on facilitators and barriers to change, and the factors that contribute to successful change. These will include questions around trust in technology and data privacy as well as efficiency and effectiveness and changes in clinician workflow and patient experience. Data gathering and analysis will be interleaved, so that later data gathering is informed by the findings from earlier analysis.

Small-scale ethnographic observations will be conducted in all settings, observing both selected clinician-patient interactions around the diagnostic process (community optometry and HES) and clinician tele-care practices (HES). 3-5 clinician-patient consultations will be observed per setting; debrief interviews with patients will cover the same themes as the interviews with practitioners but be sensitive to the different perspectives of patients and professionals. Detailed field notes will be kept of all observations. This data will also be subjected to thematic analysis, focusing on workflows, variability in workflows, and any problems experienced during the interaction (particularly related to technology use).

Patients will be invited to participate at the time that they receive their appointment letter, so that they have time to consider whether they wish to do so (for informed consent), and to plan their clinic visit time to accommodate a short interview (15 mins approx.) after their appointment. On the day of the visit the investigator will provide the patient information leaflet (PIS; **Appendix 1**) to the patient and go through it highlighting what the purpose of the study is, what it entails if the patient decides to take part and possible advantages and disadvantages and risks of taking part. When the patient has had amble time to read the PIS and ask questions regarding the study, the patient will be asked to sign an informed consent form (ICF; **Appendix 1**). Once the informed consent process is complete a copy of the ICF will be provided to the participant, and the signed form will be filed in the participant’s study records. Once the informed consent process is complete, the investigator will record the decision in the case history form.

As this is a cluster randomised clinical trial, randomisation applies at the level of entire community optometry practices. The practices randomised to the intervention arm (tele-ophthalmology) will adopt this pathway for all patient referrals to secondary care as standard practice. Patient-level consent for this study pertains to allowing use of collected data for analysis but participation in the study will not

affect patient-level care. Given the urgent presentation of the patient population we will approach for participation in this study and the fact that patient management will not be influenced by randomisation as described above, a 24-hour minimum period of consideration for patient participation is not warranted. Patients approached for participation will be given the study-specific PIS and adequate time to have any queries addressed by the clinical team before deciding on participation to the study.

Appendix 2. Participant Information Sheet and Consent Form

Patient Information Sheet

Study title: Tele-op**H**thalmology- enabl**E**d and A**R**tificial Intelligence-ready referral pathway for co**M**munity optom**E**try referral**S** or retinal disease: acluster Randomised Superiority trial with a linked Observational Diagnostic Accuracy Study

Short title: HERMES

Protocol Reference Number: BALK1006

We would like to invite you to take part in our research study. Before you decide we would like you to understand why the research is being done and what it would involve for you. One of our team will go through the information sheet with you and answer any questions you have. We'd suggest this should take about 15 minutes. Talk to others about the study if you wish.

Part 1 tells you the purpose of this study and what will happen to you if you take part. Part 2 gives you more detailed information about the conduct of the study.

Ask us if there is anything that is not clear. Take time to decide whether or not you wish to take part.

Part 1

1. What is the purpose of the study?

Early diagnosis of retinal condition (including Age-related Macular degeneration) is classified using imaging technology called Optical coherence tomography (OCT). This technology is becoming more available in community based optometry practices (high street optometry practices); however, interpreting the imaging scans can require hospital level expertise.
As a result of this, a large proportion of patients with retinal disease are incorrectly referred to hospital based eye clinics for diagnostics and disease management. This has led to an increasing pressure on hospital eye services and can cause delay in access for patients with sight threatening disease.

In this study we will involve patients with suspected retinal condition who have attended an eye examination appointment at a participating community practice. Patients that want to take part will be referred to the hospital eye service via the standard pathway for either: urgent care, routine care or not referred at all depending on the clinical assessment and OCT scan taken by the community optometrist.

The study will seek to show that we can improve patient care by using tele-Ophthalmology technologies to manage the proportion of referrals that do not need to attend hospital eye service for consultations and can be managed safely by community based optometry practices. Half of the optometry practices involved in this study will do the referrals through a 'tele-ophthalmology' process. This means that your eye scans and other clinical information will be reviewed remotely by expert clinicians at corresponding NHS eye hospitals and they will make the referral decision instead of the optometrists.

For peer review only - http://bmjopen.bmj.com/site/about/guidelines.xhtml

Your eye scans may also be processed at a later time by an Artificial Intelligence algorithm that can read the scans and also make a referral decision. The Artificial Intelligence algorithm has been developed previously through the collaboration between Moorfields Eye Hospital and Google/Deepmind. This algorithm will be introduced in the Reading Centre (Moorfields Eye Hospital NHS Trust), all data analysis will be performed within the Reading Centre (Moorfields Eye Hospital NHS Trust) and no research data will be shared or analysed externally to the research team. During this study, the Artificial Intelligence algorithm will analyse the OCT and make a recommendation on whether a referral to the hospital is needed or not; this will then be compared with the referral recommendation made by an expert clinician for your care and will not impact on your care in any way. The actual decision to refer or not will be made by a human expert in every case and not by the algorithm.

Additionally, in a sub-study we want to involve patients with suspicion of retinal disease who are already being referred to the hospital eye services via a tele-ophthalmology platform. The patient's clinical history and OCT scan will be reviewed by experts at the participating hospital eye service and a referral decision will be made remotely. This sub-study is seeking to understand what impact the introduction of tele-ophthalmology has in a real-life setting where tele–ophthalmology is already used to refer patients to the hospital eye services and also in terms of patient care and managing unnecessary referral that are made by the community based practices to the hospital based eye services.

2. Why have I been invited?

You have been invited to take part in this study because you are attending an eye examination appointment at a participating community optometry practice that undergo OCT scans and have been diagnosed with suspicious retinal disease.

3. Do I have to take part?

It is up to you to decide to join the study. We will describe the study and go through this information sheet. If you agree to take part, we will then ask you to sign a consent form. You are free to withdraw at any time without giving a reason. This would not affect the standard of care you receive. If you decide not to take part in the study, you will still have your normal eye examination and an eye scan.

4. What will happen to me if I take part?

If you decide to take part in this study, you (and if applicable a witness) will be asked to sign and date a consent form. The consent form will be signed in the presence of a trained healthcare professional who will also sign and date it. You will be provided a copy of this to keep. After the consent process you will undergo a clinical assessment and have an eye scan (OCT). If you take part in the routine care arm of the study, a referral decision will be made as usual by your community optometrist who will decide whether you will need to be; referred urgently to a hospital eye service,

For peer review only - http://bmjopen.bmj.com/site/about/guidelines.xhtml

routinely or not referred at all. The referral decision will be sent as per standard practice to the corresponding hospital eye service. If you take part in the tele-ophthalmology arm of the study, expert clinicians at the corresponding NHS eye hospital will review your eye scan and other clinical information remotely and will make the referral decision.

You will not have a choice about which arm of the study you will participate in, as the optometry practice will already have been pre-assigned either the routine referral arm or the tele-ophthalmology arm. However this will not influence in any aspect the standard of care you will receive during this visit. You will receive the same care whether you are in the routine care arm or in the tele-ophthalmology arm.
Only your community optometrist or an expert clinician will make a referral decision after reviewing your eye scan and clinical information, however you will not be able to decide who will make the referral decision. Additionally, a computer program (AI) may analyse your eye scan and give its own clinical diagnosis, however this will not influence the referral decision that will be made. By using this computer program we only want to obtain information that will help clinicians to make a better clinical decision and diagnosis in the future for people with retinal disease.

If you decide to take part in the sub-study, you will undergo the consent process as described above. After the consent process you will undergo a clinical assessment and have an eye scan (OCT). Your community optometrist will make a referral decision as usual who will decide whether you need to be referred urgently to a hospital eye service, routinely or not referred at all. After reviewing your eye scan, your community optometrist will send the referral via tele-ophthalmology to the corresponding NHS eye hospital. Expert clinicians at the corresponding NHS eye hospital will review your eye scan and other clinical information remotely and will make the referral decision.

5. What are the possible risks and benefits of taking part?

This study has no invasive testing and no therapeutic interventions; therefore there is minimal risk to patients if they choose to participate in this study. Your eyes may be dilated during your visit for the OCT scan. This will make you sensitive to light, blur your vision and may make it difficult to focus on close up objects for 4-6 hours.

We cannot promise the study will help you, but the information we get from this study will help improve the experience of care of people with retinal disease and improve the referral pathway between community optometry practices and the hospital eye service.

6. What if there is a problem?
Any complaint about the way you have been dealt with during the clinical trial or any possible harm you might suffer will be addressed. The detailed information concerning this is given in Part 2 of this information sheet. If you have any concerns or complaints you should contact your study doctor in the first instance.

7. Will my taking part in the study be kept confidential?
Yes. We will follow ethical and legal practice and all information about you will be handled in confidence. The details are included in Part 2.

For peer review only - http://bmjopen.bmj.com/site/about/guidelines.xhtml

8. Contact Details

Principal investigator

Name: Tel. Number:

Research Project Manager

Name: Tel. Number:

This completes Part 1 of the Information Sheet.

If the information in Part 1 has interested you and you are considering participation, please read the additional information in Part 2 before making any decision.

Part 2

9. What if relevant new information becomes available?

On receiving new information, we might consider it to be in your best interests to withdraw you from the study. If so, we will explain the reasons and arrange for your care to continue. If the study is stopped for any other reason, we will tell you why and arrange your continuing care.

10. What will happen if I don't want to carry on with the study?

If you withdraw from the study, we will destroy all your identifiable information, but we will need to use the data collected up to your withdrawal.

11. What if there is a problem?

If you have a concern about any aspect of this study, you should ask to speak to the researchers who will do their best to answer your questions …. If you remain unhappy and wish to complain formally, you can do this by contacting the PALS team at ….

If taking part in this research project harms you, there are no special compensation arrangements. If you are harmed due to someone's negligence, then you may have grounds for legal action but you may have to pay for it. Regardless of this, if you wish to complain about any aspect of the way you have been approached or treated during the course of this study, the normal National Health Service complaints mechanisms will be available to you.

12. Will use of my data meet the GDPR rules?

Yes, all data will be handled in accordance with the General Data Protection Regulations (GDPR) and UK Data Protection Act 2018, The Research Governance

For peer review only

For peer review only - http://bmjopen.bmj.com/site/about/guidelines.xhtml

Framework for Health and Social Care and the conditions for the Research Ethics Committee favorable opinion.

Moorfields Eye Hospital NHS Trust is the Sponsor for this study based in the United Kingdom. We will be using information from you and/or your medical records in order to undertake this study and will act as the data controller for this study. This means that we are responsible for looking after your information and using it properly.

Your rights to access, change or move your information are limited, as we need to manage your information in specific ways in order for the research to be reliable and accurate. If you withdraw from the study, we will keep the information about you that we have already obtained.

You can find out more about how we use your information by visiting www.moorfields.nhs.uk/content/how-we-use-your-information or please contact your research team (study team contact details can be found in your participant information sheet).

(SITE NAME) will use your name, and contact details to contact you about the research study, and make sure that relevant information about the study is recorded for your care, and to oversee the quality of the study. Moorfields Eye Hospital and their study collaborators (which may include Universities) will receive information from your medical record for the purposes of the study but without your name or any other personal details. (SITE NAME) will pass to Moorfields Eye Hospital this information collected from you and/or your medical records. The only people in (SITE NAME) who will have access to information that identifies you will be people who need to contact you to arrange appointments or audit the data collection process. The people who analyse the information will not be able to identify you and will not be able to find out your name or contact details. Data collected for this study, without your name or any other personal details, can be used for future research. If you agree to take part in this study, we will also specifically ask for your permission to use your data for future research. If you don't want your data to be used for any future research, you can still participate in this study.

Moorfields Eye Hospital will keep identifiable information about you from this study without your personal details for 15 years.

13. Will my GP be informed of my involvement?

With your permission, your GP, and other doctors who may be treating you, will be notified that you are taking part in this study.

14. What will happen to the results of the research study?

The results of the study will be available after it finishes and will usually be published in a medical journal or be presented at a scientific conference. The data will be anonymous and none of the patients involved in the trial will be identified in any report or publication.

Should you wish to see the results, or the publication, please ask your study doctor.

15. Who is organising and funding the research?

For peer review only - http://bmjopen.bmj.com/site/about/guidelines.xhtml

The study is organized by Moorfields Eye Hospitals NHS Foundation Trust and funded by the National Institute for Health Research.

16. Who has reviewed the study?

All research in the NHS is looked at by independent group of people, called a Research Ethics Committee, to protect your interests. This study has been reviewed and given favourable opinion by London – Bromley Research Ethics Committee.

17. Further information and contact details

You are encouraged to ask any questions you wish, before, during or after your treatment. If you have any questions about the study, please speak to your study Optometrists or doctor, who will be able to provide you with up to date information about the procedure(s) involved. If you wish to read the research on which this study is based, please ask your study optometrists or doctor. If you require any further information or have any concerns while taking part in the study please contact one of the following people:

Principle Investigator

Name: Tel. Number:

Research Project Manager

Name: Tel. Number:

If you decide you would like to take part then please read and sign the consent form. You will be given a copy of this information sheet and the consent form to keep. A copy of the consent form will be filed in your patient notes, one will be filed with the study records and one may be sent to the Research Sponsor.

You can have more time to think this over if you are at all unsure.

Thank you for taking the time to read this information sheet and to consider this study.

For peer review only

For peer review only - http://bmjopen.bmj.com/site/about/guidelines.xhtml

Appendix 3. Digital Pathway Decisions

The following scenarios can occur in the intervention arm:

1. Community optometrist recommendation: **Refer urgently to HES**—> OCT scan and clinical data are transferred to 'tele-HES' and reviewed within 48h remotely by human expert —> Referral Decision is made in 'tele-HES' (refer urgently, refer routinely, don't refer) and fed-back to the community optometry practice to be implemented.
2. Community optometrist recommendation: **Refer routinely to HES**—> OCT scan and clinical data are transferred to 'tele-HES' and reviewed within 48h remotely by human expert —> Referral Decision is made in 'tele-HES' (refer urgently, refer routinely, don't refer) and fed-back to the community optometry practice to be implemented.
3. Community optometrist recommendation: **Don't refer to HES**—> OCT scan and clinical data are transferred to 'tele-HES' and reviewed within 48h remotely by human expert —> Referral Decision is made in 'tele-HES' (refer urgently, refer routinely, don't refer) and fed-back to the community optometry practice to be implemented.

Appendix 4. Statistical Consideration

The trial statisticians based at King's Clinical Trial Unit will write the statistical analysis plan before database lock and will perform the analysis using the Stata software (StataCorp, College Station, TX, USA).

4.1 Sample Size Calculation

The primary outcome is the proportion of false positive referrals. Under the current system an audit conducted at Moorfields Eye Hospital NHS Foundation Trust in September 2018 showed that 70 % of retinal referrals were false positive.[1] A pilot study on 40 patients conducted in three optometry practices showed that this could be reduced by 60 %.[1] A 95 % confidence interval computed by the modified Wald Method as advised by Agresti and Coull would extend 44.6% to 73.7%.[2] There is consensus amongst clinicians however that given the savings to the NHS and benefit to patients, slightly smaller differences would be important to detect and we have powered the study to examine a reduction to 40 % false referrals. Whilst smaller differences might yet be important it would seem unethical to power for lower than 40 % based on the observed data and clinical expertise in this area.

Although decisions for patients are made on an individual basis each patient cannot be assumed to generate independent information since they will be clustered within optometry practices. The correlation of information from patients within a cluster (the intracluster correlation) is estimated to be 0.15. We have based this intracluster correlation on previous work conducted in ophthalmology [3, 4] with a clinical outcome similar to this study. Since this is an estimate we have increased the intracluster correlation slightly to allow for the potential that patients within the same optometry practice may be more similarly managed than patients within different practices although clinical consensus is that clinical signs are more likely to impact upon decision making for referral than individual optometrist attitudes. Using nQuery software version 8.3.10, a hierarchical 2-level mixed effects model was used to calculate the required sample size. 24/26 clusters split between the study arms in a 1:1 ratio need to recruit an average of 12/10 patients per clusters (12 patients if 24 clusters, 10 patients if 26 clusters) in order to achieve 89.27% power to detect a difference in the proportion of false positive referrals of 30% (a drop from the current rate of 70% to the clinically relevant rate of 40%). This calculation assumes an intracluster correlation of 0.15 and the test is performed at the 5% significance level.

A total of 288 patients (based on an average of 12 patients recruited at 24 clusters, 144 per study arm) would therefore be needed to complete the data analysis with sufficient statistical power. To allow for an anticipated 15% drop our rate (patients are likely to be elderly and have comorbidity causing motion artefacts and some images may be ungradable), the total sample size is 340 patients (170 per study arm).

The sample size of the RCT and pragmatic sub-study combined will also enable the AI observational diagnostic accuracy study to obtain robust estimates of sensitivity and specificity. All 500 patients (accounting for the anticipated drop-out rate) will be included in the AI study. Classifications will be

made without additional clinical information. Research from our group suggests that the diagnostic accuracy of the Moorfields-DeepMind AI will be as high as 95%.[5] This combined sample of 500 patients with 475 patients being correctly diagnosed would produce a two-sided 95.0% confidence interval with a width of 0.039. The sample from the RCT alone -288 patients with 274 being correctly diagnosed - produce a two-sided 95.0% confidence interval with a width of 0.052. PASS has been used to calculated these widths.

Recruitment plan

On the basis of feedback provided by optometry practices already identified and interested in participating in the study, an average of 3 eligible patients can be approached to consider participation in the study per month per cluster, with a range of 2-5 patients based on the size of the optometry practice. However, it is also expected that 35% of potential patients will decline to participate. Currently, different sites are at different stages of readiness for commencing recruitment and therefore a staggered start to recruitment over 3 months is embedded in the recruitment plan. Based on these conservative estimates a recruitment period of 12 months with a staggered initiation over the first 3 months will be sufficient to approach 521 patients, of whom it is expected that 340 patients will be recruited to the study. A smaller practice only approaching 2 patients per month will require 11 months to recruit 12 patients (accounting for drop out and decline to participate). If the practice was one of the last to start recruitment and so started 3 months into the recruitment window, they would still manage to recruit 10 patients in 9 months (accounting for drop out and decline to participate). A larger practice in the same catchment area will be able to compensate by over-recruiting up to a set maximum of 16 patients. The range of cluster size will thus be 10-16 patients.

The 2 additional randomised community practices allow the potential to increase the number of clusters to 26 if further acceleration of recruitment is required. 26 clusters will be required to recruit 306 patients overall with an average of 10 patients per cluster (and a minimum of 8 patients per cluster) in order to achieve 89.27% power to detect a difference in the proportion of false positive referrals of 30% - using the same parameters as the sample size calculation for the 24 clusters.

Pragmatic Sub-Study

Manchester Eye Hospital and its local area, a site included in the original protocol, has already moved to a tele-ophthalmology referral pathway as part of a commissioning change across the local region. This change in standard care provides a unique opportunity to examine whether tele-ophthalmology works under usual conditions within the NHS. This sub-study will allow us to measure and visualise variation in quality of health care within a local region to inform our inferences from the RCT on how the tele- ophthalmology pathway will perform within a real-life setting.

From this study, key estimate statistics will be calculated including the overall rate of referral to HES, the false positive (referral) and false negative rate against the Reference Standard and the proportion of wrong diagnosis and wrong referral urgency. These shall be compared to the rates found for the intervention arm of the main RCT.

Recruiting 18 patients from each of 12 tele-optometry practices (for a total of 216 patients) will allow the proportion of false positive referrals to be produced with a 95% confidence interval with a width less than 0.187. This was calculated based on confidence intervals for one proportion within a cluster-randomised design with an intracluster correlation of 0.15. A total of 216 patients (based on an average of 18 patients recruited from 12 clusters) would therefore provide a certain degree of precision. To allow for an anticipated 15% drop our rate (patients are likely to be elderly and have comorbidity causing motion artefacts and some images may be ungradable), the total sample size is 254 patients. It is expected that 35% of participants will decline to participate and so 390 patients would need to be approached. These patients can be recruited over a period of 18 months with an average of 3 patients approached by each practice per month.

4.2 Statistical analysis

The primary analysis will be conducted following an intention to treat principle where all randomised patients are analysed in their allocated group whether or not they receive their randomised management plan. Baseline characteristics will be summarised for each management group (standard care or tele-ophthalmology). We will report the number of clusters in each group and the size of clusters. Continuous data will be summarised using means and standard deviations if data appear Gaussian or medians and interquartile ranges. Categorical data will be reported as proportions and percentages.

The primary outcome is the proportion of false referrals. The outcome is measured at the patient level. This will be compared between management groups using logistic regression adjusting for clustered centres. Outcomes will be reported as adjusted odds ratios. We will also report the difference in proportions with a 95 % confidence interval as per the CONSORT extension for cluster randomised controlled trials. We will report false referral rates with 95 % confidence intervals computed by the exact binomial method by diagnosis and by level of urgency. The results will be presented at the cluster level and overall.

Secondary outcomes such as time from referral to review in HES and treatment will be analysed in a similar fashion. The percentage of patients experiencing adverse events in the two groups will be reported with 95 % confidence intervals computed by the exact binomial method.

Loss to follow-up will be examined by study arm. Reasons for missingness may be important and these will be investigated using logistic regression of covariates based on an indicator of missingness. An available case analysis will be reported along with an analysis using imputed data based on best- and worst-case scenarios. Since this is a cluster RCT we will also examine and report missingness by cluster.

No formal interim analysis is planned but reports concerning patient safety will be prepared for review by the Independent Data Monitoring Committee. All tests will be two sided and will be assessed at the 5 % significance level unless otherwise specified. All confidence intervals will be 95 % and two sided. A detailed statistical analysis plan will be agreed with the Trial Steering Group prior to any analysis of locked data. All statistical analysis will be performed using Stata (StataCorp, College Station, TX, USA).

Statisticians analysing the data will be masked to the management group status of the practise and patient.

In the AI diagnostic accuracy study, we will report estimates of sensitivity and specificity of the DeepMind algorithm for referral decisions with 95 % confidence intervals. Our primary analysis will combine urgent and standard referral to HES and compare against no referral to HES but a sensitivity analysis will be conducted to evaluate urgent referrals. The referral outcome (refer routinely, refer urgently, don't refer) will be cross tabulated for the DeepMind algorithm and each of the RCT treatment arms (community optometry and 'tele-HES'), the pragmatic sub-study, and for the Reference Standard.

Appendix 5. Case Report Form

Moorfields Eye Hospital, Case Report Form
BALK1006

Moorfields Eye Hospital NHS Foundation Trust NHS

Study No: ☐☐-☐☐☐

HERMES Study
Completing Case Report Forms (CRFs)

This document has been created to provide guidelines about completing clinical trial case report forms at Moorfields Eye Hospital (MEH). The information has been extracted from the standard operating procedures (SOPs) 'Completing, Correcting & Signing off Case Report Forms (CRF_S07)' that have been developed by the Research & Development department at Moorfields.

1. The CRF must be completed as soon as possible after the patient has been assessed or during the assessment if the CRF is the source data.

2. CRFs must be completed using a black ink ballpoint pen.

3. If the CRF is printed on carbonless duplication paper, a suitable separator must be inserted under the form being completed.

4. Data entry into the CRF must be complete as without omissions. If data are unavailable then 'unknown', 'missing', 'test not done' etc. should be inserted. The ambiguous phrase, 'not available' should be avoided.

5. All entries into the CRF must be accurate, legible and verifiable with the source data in the medical records (unless the CRF is the source data). Data must not be invented – this is fraud.

   N.B. Whenever a subject has been seen by clinical staff for the purposes of a clinical trial, the time, date and reason for visit must always be entered into the subject's corresponding hospital notes. Copies of trial investigations/results that are clinically significant or have an impact on the patient's clinical care must also be filed in the medical notes.

6. Any discrepancies between the CRF and the source data should be explained and the significance noted in the CRF and/or patient's medical records.

7. All CRF data derived from source documents must be transcribed exactly.

8. For laboratory values that fall outside the laboratory's reference range or trial specific range or when a value shows a significant variation from one assessment to the next, this should be commented on and the significance noted in the CRF and/or patient's medical records.

9. The subject's identity should remain confidential at all times and as such the trial subject must only be identified in the CRF using a trial number or code.

10. Entries into the CRF must never be overwritten.

11. Corrections to the CRF must be made as follows:

    - An incorrect entry must be deleted with a single line through the text allowing the incorrect entry to remain legible. Correction fluid must never be used and entries must not be obliterated.
    - The correct data must be entered.
    - The correction must be initialled and dated and an explanation given of the correction, if applicable.

12. The CRF must be signed and dated where indicated, by the chief/principal investigator or designee (for example, research nurse at the end of an assessment) to assert that he/she believes the data is completed and correct.

For peer review only - http://bmjopen.bmj.com/site/about/guidelines.xhtml

Moorfields Eye Hospital, Case Report Form
BALK1006

Moorfields Eye Hospital NHS Foundation Trust NHS

Study No: ☐☐ - ☐☐☐

HERMES Study

PATIENT DEMOGRAPHICS (Community Optometry) **Page 1/2**

**ALL FIELDS ARE MANDATORY**		
**Patient Details**		
Study site	MEH ☐ Manchester ☐ Birmingham ☐<br>Central Middlesex ☐ North West Anglia ☐	
Optometrist site		
Randomisation Cluster number		
Randomisation Arm	Control ☐	Intervention ☐
Age		(≥18) ☐☐☐
Sex	Male ☐	Female ☐
Medical history	Yes No<br>Heart attack ☐ ☐<br>COPD ☐ ☐<br>Diabetes ☐ ☐<br>Hypertension ☐ ☐	Yes No<br>TIA/Stroke ☐ ☐<br>Impaired Mobility ☐ ☐<br>Asthma ☐ ☐<br>Other ☐ ☐
If other please specify		
Medication for eye condition	Yes ☐	No ☐
If yes specify glaucoma drops?	Prostaglandins ☐ CA inhibitors ☐	B-blockers ☐
If yes specify AREDS	Yes ☐	No ☐
Smoker?	Ex-smoker ☐ Smoker ☐	Non-smoker ☐
	**Right Eye**	**Left eye**
Ocular history	Yes No<br>Wet AMD ☐ ☐<br>Dry AMD ☐ ☐<br>Central Serous Chorioretinopathy ☐ ☐<br>Central Retinal Vein Occlusion ☐ ☐<br>Branch Retinal Vein Occlusion ☐ ☐<br>Diabetic Macular Oedema ☐ ☐<br>Inherited Eye Disease ☐ ☐<br>Other ☐ ☐	Yes No<br>Wet AMD ☐ ☐<br>Dry AMD ☐ ☐<br>Central Serous Chorioretinopathy ☐ ☐<br>Central Retinal Vein Occlusion ☐ ☐<br>Branch Retinal Vein Occlusion ☐ ☐<br>Diabetic Macular Oedema ☐ ☐<br>Inherited Eye Disease ☐ ☐<br>Other ☐ ☐
If other, specify		

For peer review only - http://bmjopen.bmj.com/site/about/guidelines.xhtml

Moorfields Eye Hospital, Case Report Form
BALK1006

Moorfields Eye Hospital NHS Foundation Trust NHS

Study No: ☐☐ - ☐☐☐

HERMES Study

PATIENT DEMOGRAPHICS (Community Optometry) **Page 2/2**

ALL FIELDS ARE MANDATORY

Patient Details

		Yes	No		Yes	No
Previous eye procedures	Cataract surgery	☐	☐	Cataract surgery	☐	☐
	Glaucoma surgery	☐	☐	Glaucoma surgery	☐	☐
	Eyelid surgery	☐	☐	Eyelid surgery	☐	☐
	Other	☐	☐	Other	☐	☐
If other specify						

Comments

For peer review only

I can confirm that the patient meets all eligibility criteria for the study and I have completed this form in full and take full responsibility for any missing data.

Signed:	Print:	Date:
		☐☐/☐☐/☐☐☐☐

Office use only, data entry completed by:

Signed:	Print:	Date:
		☐☐/☐☐/☐☐☐☐

For peer review only - http://bmjopen.bmj.com/site/about/guidelines.xhtml

Moorfields Eye Hospital, Case Report Form
BALK1006

Moorfields Eye Hospital NHS Foundation Trust NHS

Study No: ☐☐ - ☐☐☐

HERMES Study

BASELINE VISIT (Community Optometry) **Page 1/2**

**ALL FIELDS ARE MANDATORY**		
**Baseline Exam**		
Date of visit	*(dd mm yyyy)* ☐☐ / ☐☐ / ☐☐☐☐	
Years since diagnosis of any retinal disease?	☐☐	
OCT Device	Topcon 3D OCT-2000 ☐ Heidelberg OCT1 ☐ Other ☐	
If other specify		
	**Right Eye**	**Left eye**
Visual acuity (ETDRS)	*(ETDRS letters, range 0-95)* ☐☐	*(ETDRS letters, range 0-95)* ☐☐
Visual acuity (Snellen)	☐ / ☐☐	☐ / ☐☐
Diagnosis by the Optometrist	Yes No<br>Wet AMD ☐ ☐<br>Dry AMD ☐ ☐<br>Central Serous Chorioretinopathy ☐ ☐<br>Central Retinal Vein Occlusion ☐ ☐<br>Branch Retinal Vein Occlusion ☐ ☐<br>Diabetic Macular Oedema ☐ ☐<br>Inherited Eye Disease ☐ ☐<br>Other ☐ ☐	Yes No<br>Wet AMD ☐ ☐<br>Dry AMD ☐ ☐<br>Central Serous Chorioretinopathy ☐ ☐<br>Central Retinal Vein Occlusion ☐ ☐<br>Branch Retinal Vein Occlusion ☐ ☐<br>Diabetic Macular Oedema ☐ ☐<br>Inherited Eye Disease ☐ ☐<br>Other ☐ ☐
If other specify		
Clinical findings	Yes No<br>Macular Haemorrhage ☐ ☐<br>Other RetinalHaemorrhage ☐ ☐<br>Exudates ☐ ☐<br>Disc Swelling ☐ ☐<br>Macular Atrophy ☐ ☐<br>Cotton Wool Spot ☐ ☐<br>Other ☐ ☐	Yes No<br>Macular Haemorrhage ☐ ☐<br>Other RetinalHaemorrhage ☐ ☐<br>Exudates ☐ ☐<br>Disc Swelling ☐ ☐<br>Macular Atrophy ☐ ☐<br>Cotton Wool Spot ☐ ☐<br>Other ☐ ☐
If other specify		

For peer review only

For peer review only - http://bmjopen.bmj.com/site/about/guidelines.xhtml

Moorfields Eye Hospital, Case Report Form
BALK1006

Moorfields Eye Hospital NHS Foundation Trust NHS

Study No: ☐☐ - ☐☐☐

HERMES Study

BASELINE VISIT (Community Optometry) **Page 2/2**

ALL FIELDS ARE MANDATORY					
**Baseline Exam**	**Right Eye**		**Left eye**		
Intraocular pressure	*(0-60)* ☐☐		*(0-60)* ☐☐		
OCT taken?	Yes ☐	No ☐	Yes ☐	No ☐	
OCT qualitative	SRF ☐ PED ☐	IRF ☐ SHRM ☐	SRF ☐ PED ☐	IRF ☐ SHRM ☐	
Referral Recommendation by Optometrist (both arms)	Urgent ☐	Routine ☐	No referral ☐		
If not referred, specify reason					

Comments:

I have completed this form in full and take full responsibility for any missing data.

**Signed:**	**Print:**	**Date:**
		☐☐ / ☐☐ / ☐☐☐☐
**Office use only, data entry completed by:**		
**Signed:**	**Print:**	**Date:**
		☐☐ / ☐☐ / ☐☐☐☐

For peer review only - http://bmjopen.bmj.com/site/about/guidelines.xhtml

For peer review only

Study No: ☐☐ - ☐☐☐

HERMES Study

REFERRALS (Community Optometry) **Page 1/3**

**ALL FIELDS ARE MANDATORY**	
**Referral Details**	
Has referral been generated?	*(control arm)* Yes ☐ No ☐
If yes please specify date	*(dd mm yyyy)* ☐☐ / ☐☐ / ☐☐☐☐
Referral reference number	*(control arm)*
Referral system	*(control arm)* Post ☐ Electronic ☐ Other ☐
If other, please specify	
Have OCT scans been uploaded on eCRF?	*(both arms)* Yes ☐ No ☐
If no, specify reason	
Has clinical data been uploaded on eCRF?	*(intervention arm)* Yes ☐ No ☐
If no, specify reason	
Optometrist referral recommendation accepted after HES triage?	*(both arms)* Yes ☐ No ☐
If yes, eye on which referral decision was based	Right ☐ Left ☐ Both ☐
If no, referral decision after HES triage	Urgent ☐ Routine ☐ No referral ☐
Reason why recommendation was not accepted ***(field to be completed by HES)***	

For peer review only

For peer review only - http://bmjopen.bmj.com/site/about/guidelines.xhtml

Moorfields Eye Hospital, Case Report Form
BALK1006

Moorfields Eye Hospital NHS Foundation Trust NHS

Study No: ☐☐-☐☐☐

HERMES Study

REFERRALS (tele-HES) **Page 2/3**

**ALL FIELDS ARE MANDATORY**		
**Tele - HES Review**		
OCT from referral reviewed by tele-HES?	Yes ☐	No ☐
	**Right Eye**	**Left eye**
OCT from referral *qualitative by tele-HES*	SRF ☐ IRF ☐<br>PED ☐ SHRM ☐	SRF ☐ IRF ☐<br>PED ☐ SHRM ☐
Clinical findings	Yes No<br>Macular Haemorrhage ☐ ☐<br>Other Retinal Haemorrhage ☐ ☐<br>Exudates ☐ ☐<br>Disc Swelling ☐ ☐<br>Macular Atrophy ☐ ☐<br>Cotton Wool Spot ☐ ☐<br>Other ☐ ☐	Yes No<br>Macular Haemorrhage ☐ ☐<br>Other Retinal Haemorrhage ☐ ☐<br>Exudates ☐ ☐<br>Disc Swelling ☐ ☐<br>Macular Atrophy ☐ ☐<br>Cotton Wool Spot ☐ ☐<br>Other ☐ ☐
If other specify		
**Tele-HES Review**	**Right Eye**	**Left eye**
Diagnosis from referring optometrist confirmed by tele-HES	Yes ☐	No ☐
Diagnosis by tele-HES	Yes No<br>Wet AMD ☐ ☐<br>Dry AMD ☐ ☐<br>Central Serous Chorioretinopathy ☐ ☐<br>Central Retinal Vein Occlusion ☐ ☐<br>Branch Retinal Vein Occlusion ☐ ☐<br>Diabetic Macular Oedema ☐ ☐<br>Inherited Eye Disease ☐ ☐<br>Other ☐ ☐	Yes No<br>Wet AMD ☐ ☐<br>Dry AMD ☐ ☐<br>Central Serous Chorioretinopathy ☐ ☐<br>Central Retinal Vein Occlusion ☐ ☐<br>Branch Retinal Vein Occlusion ☐ ☐<br>Diabetic Macular Oedema ☐ ☐<br>Inherited Eye Disease ☐ ☐<br>Other ☐ ☐
If other specify		

For peer review only - http://bmjopen.bmj.com/site/about/guidelines.xhtml

Moorfields Eye Hospital, Case Report Form
BALK1006

Moorfields Eye Hospital NHS Foundation Trust NHS

Study No: ☐☐ - ☐☐☐

HERMES Study

REFERRALS (tele-HES) **Page 3/3**

**ALL FIELDS ARE MANDATORY**	
**Tele - HES Review**	
Referral recommendation by referring optometrist confirmed by tele-HES	Yes ☐ No ☐
If yes, eye on which referral decision was based	Right ☐ Left ☐ Both ☐
Referral Decision by tele-HES	Urgent ☐ Routine ☐ No referral ☐

Comments:

For peer review only

I have completed this form in full and take full responsibility for any missing data.

**Signed:**	**Print:**	**Date:**
		☐☐ / ☐☐ / ☐☐☐☐
**Office use only, data entry completed by:**		
**Signed:**	**Print:**	**Date:**
		☐☐ / ☐☐ / ☐☐☐☐

For peer review only - http://bmjopen.bmj.com/site/about/guidelines.xhtml

Moorfields Eye Hospital, Case Report Form
BALK1006

Moorfields Eye Hospital NHS Foundation Trust NHS

Study No: ☐☐-☐☐☐

HERMES Study

HES First Visit **Page 1/2**

**ALL FIELDS ARE MANDATORY**				
**HES Review**				
Follow up status	Attended ☐	Cancelled ☐	DNA ☐	
Date of consultation	***(dd mm yyyy)*** ☐☐/☐☐/☐☐☐☐			
Change in eye medication	Yes ☐	No ☐		
If yes, specify				
Date of first treatment (if applicable)	***(dd mm yyyy)*** ☐☐/☐☐/☐☐☐☐			

	**Right Eye**	**Left eye**
Intraocular pressure	***(0-60)*** ☐☐	***(0-60)*** ☐☐
OCT taken?	Yes ☐ No ☐	Yes ☐ No ☐
OCT qualitative	SRF ☐ IRF ☐<br>PED ☐ SHRM ☐	SRF ☐ IRF ☐<br>PED ☐ SHRM ☐
Visual acuity (ETDRS)	***(ETDRS letters, range 0-95)*** ☐☐	***(ETDRS letters, range 0-95)*** ☐☐
Visual acuity (Snellen)	☐ / ☐☐	☐ / ☐☐

Clinical findings by HES	Right Eye Yes	Right Eye No	Left Eye Yes	Left Eye No
Macular Haemorrhage	☐	☐	☐	☐
Other Retinal Haemorrhage	☐	☐	☐	☐
Exudates	☐	☐	☐	☐
Disc Swelling	☐	☐	☐	☐
Macular Atrophy	☐	☐	☐	☐
Cotton Wool Spot	☐	☐	☐	☐
Other	☐	☐	☐	☐
If other specify				

Diagnosis by HES	Right Eye Yes	Right Eye No	Left Eye Yes	Left Eye No
Wet AMD	☐	☐	☐	☐
Dry AMD	☐	☐	☐	☐
Central Serous Chorioretinopathy	☐	☐	☐	☐
Central Retinal Vein Occlusion	☐	☐	☐	☐
Branch Retinal Vein Occlusion	☐	☐	☐	☐
Diabetic Macular Oedema	☐	☐	☐	☐
Inherited Eye Disease	☐	☐	☐	☐
Other	☐	☐	☐	☐
If other specify				

For peer review only

For peer review only - http://bmjopen.bmj.com/site/about/guidelines.xhtml

Moorfields Eye Hospital, Case Report Form
BALK1006

Moorfields Eye Hospital NHS Foundation Trust NHS

Study No: ☐☐ - ☐☐☐

HERMES Study
HES First Visit

Page 2/2

**ALL FIELDS ARE MANDATORY**		
**HES Review**	**Right Eye**	**Left eye**
	Yes / No	Yes / No
Additional Diagnostic Procedures	OCT - Angio ☐ ☐	OCT - Angio ☐ ☐
	IGCA ☐ ☐	IGCA ☐ ☐
	FA ☐ ☐	FA ☐ ☐
	Optos ☐ ☐	Optos ☐ ☐
	Ultrasound B Scan ☐ ☐	Ultrasound B Scan ☐ ☐
	Visual Field Test ☐ ☐	Visual Field Test ☐ ☐
	Other ☐ ☐	Other ☐ ☐
If other specify		

Comments:

I have completed this form in full and take full responsibility for any missing data.

**Signed:**	**Print:**	**Date:**
		☐☐ / ☐☐ / ☐☐☐☐
**Office use only, data entry completed by:**		
**Signed:**	**Print:**	**Date:**
		☐☐ / ☐☐ / ☐☐☐☐

For peer review only - http://bmjopen.bmj.com/site/about/guidelines.xhtml

Moorfields Eye Hospital, Case Report Form
BALK1006

Moorfields Eye Hospital NHS Foundation Trust NHS

Study No: ☐☐-☐☐☐

HERMES Study
READING CENTRE

Page 1/2

**ALL FIELDS ARE MANDATORY**					
**Reference Standard**	**Right Eye**			**Left eye**	
Diagnosis by the reference standard		Yes	No	Yes	No
	Wet AMD	☐	☐	☐	☐
	Dry AMD	☐	☐	☐	☐
	Central Serous Chorioretinopathy	☐	☐	☐	☐
	Central Retinal Vein Occlusion	☐	☐	☐	☐
	Branch Retinal Vein Occlusion	☐	☐	☐	☐
	Diabetic Macular Oedema	☐	☐	☐	☐
	Inherited Eye Disease	☐	☐	☐	☐
	Other	☐	☐	☐	☐
If other specify					
Referral decision by reference standard	Urgent ☐	Routine ☐		No referral ☐	
**Artificial Intelligence Moorfields DeepMind**	**Right Eye**			**Left eye**	
Deep-Mind Diagnosis of retinal disease		Yes	No	Yes	No
	Wet AMD	☐	☐	☐	☐
	Dry AMD	☐	☐	☐	☐
	Central Serous Chorioretinopathy	☐	☐	☐	☐
	Central Retinal Vein Occlusion	☐	☐	☐	☐
	Branch Retinal Vein Occlusion	☐	☐	☐	☐
	Diabetic Macular Oedema	☐	☐	☐	☐
	Inherited Eye Disease	☐	☐	☐	☐
	Other	☐	☐	☐	☐
If other specify					
Referral decision by Moorfields DeepMind	Urgent ☐	Routine ☐		No referral ☐	

For peer review only

For peer review only - http://bmjopen.bmj.com/site/about/guidelines.xhtml

Moorfields Eye Hospital, Case Report Form
BALK1006

Moorfields Eye Hospital NHS Foundation Trust NHS

Study No: ☐☐ - ☐☐☐

HERMES Study

READING CENTRE

Page 2/2

**ALL FIELDS ARE MANDATORY**	
**Artificial Intelligence Moorfields-DeepMind**	
Time from receiving the OCT scans and a referral decision (hours)	*(hours)* ☐☐
End-to-end inference speed of technical infrastructure supporting the AI DSS	*(minutes)* ☐☐
Any technical issues	Yes ☐ No ☐
If yes, specify details	

Comments:

I have completed this form in full and take full responsibility for any missing data.

**Signed:**	**Print:**	**Date:**
		☐☐/☐☐/☐☐☐☐
**Office use only, data entry completed by:**		
**Signed:**	**Print:**	**Date:**
		☐☐/☐☐/☐☐☐☐

For peer review only - http://bmjopen.bmj.com/site/about/guidelines.xhtml

Moorfields Eye Hospital, Case Report Form
BALK1006

Moorfields Eye Hospital NHS Foundation Trust NHS

Study No: ☐☐ - ☐☐☐

HERMES Study

APPENDIX 1 – PROTOCOL DEVIATION

ALL FIELDS ARE MANDATORY	
**Deviation Details**	
Deviation date	*(dd mm yyyy)* ☐☐ / ☐☐ / ☐☐☐☐
Type of deviation	
Any outcomes or actions	
Deviation date	*(dd mm yyyy)* ☐☐ / ☐☐ / ☐☐☐☐
Type of deviation	
Any outcomes or actions	
Deviation date	*(dd mm yyyy)* ☐☐ / ☐☐ / ☐☐☐☐
Type of deviation	
Any outcomes or actions	
Deviation date	*(dd mm yyyy)* ☐☐ / ☐☐ / ☐☐☐☐
Type of deviation	
Any outcomes or actions	

For peer review only

I have completed this form in full and take full responsibility for any missing data.

**Signed:**	**Print:**	**Date:**
		☐☐ / ☐☐ / ☐☐☐☐
**Office use only, data entry completed by:**		
**Signed:**	**Print:**	**Date:**
		☐☐ / ☐☐ / ☐☐☐☐

For peer review only - http://bmjopen.bmj.com/site/about/guidelines.xhtml

Moorfields Eye Hospital, Case Report Form
BALK1006

Moorfields Eye Hospital NHS Foundation Trust NHS

Study No: ☐☐ - ☐☐☐

HERMES Study

APPENDIX 2 – EARLY STUDY WITHDRAWAL

ALL FIELDS ARE MANDATORY	
**Withdrawal Details**	
Did the patients discontinue the trial prematurely for reasons other than being referred back to hospital care?	Yes ☐ No ☐
Date of premature Study Discontinuation	*(dd mm yyyy)* ☐☐ / ☐☐ / ☐☐☐☐
Primary reason for discontinuation *(tick one box only)*	Patient withdraws consent ☐<br>*If known, state reason:*<br><br>Patient is non-compliant ☐<br>Patient is lost to follow up ☐<br>Investigator feels that it is in the patient's best interest due to adverse event ☐<br>*Related AE No:*<br><br>Other reason for discontinuation ☐<br>*If Other specify:*
Does the patient still agree to have their data collected and analysed as part of intent to treat analysis?	Yes ☐ No ☐

I have completed this form in full and take full responsibility for any missing data.

**Signed:**	**Print:**	**Date:**
		☐☐ / ☐☐ / ☐☐☐☐
**Office use only, data entry completed by:**		
**Signed:**	**Print:**	**Date:**
		☐☐ / ☐☐ / ☐☐☐☐

For peer review only - http://bmjopen.bmj.com/site/about/guidelines.xhtml

Appendix 6. Trial Documents and Subject Records

6.1 CRFs and Source Document Identification

We will establish a hub and spoke structure, where each community optometry practice liaises with its local hospital site (Moorfields Eye Hospital NHS Foundation Trust, Birmingham University Hospitals NHS Foundation Trust, Central Middlesex Hospital at London North West University Healthcare NHS Trust, and North West Anglia NHS Foundation Trust) for the day-to-day operation of the trial, through the site coordinator located at each site. 8-10 optometry practices will be located in the catchment area of Moorfields Eye Hospital NHS Foundation Trust (4-5 control and 4-5 intervention), 4-6 in the catchment area of North West London NHS Foundation Trust (Central Middlesex Hospital) (2-3 control and 2-3 intervention), 4-6 in the catchment area of North West Anglia NHS Foundation Trust (2-3 control and 2-3 intervention) and 4-6 in the catchment area of Birmingham University Hospitals NHS Foundation Trust (2-3 control and 2-3 intervention). All sites, including community optometry and hospital sites transfer data (OCT and clinical data) to Moorfields Reading Centre. The digital referral platform will be used in the 12 intervention optometry practices and the 4 HES; OCTs and clinical data from patients in the intervention optometry practices will be transferred to HES via a digital referral platform for remote review ('tele-HES') by local human experts.

Both the control arm and interventional arm will use the trial database to complete the eCRF and securely upload OCT scans. The interventional arm also transfers OCT's to the patient's hub hospital via a secure tele-ophthalmology platform. The scans and data will then be matched with the relevant trial data in the eCRF database.

For the AI Diagnostic Accuracy study, the pseudonymised OCT scans will be securely transmitted from the Moorfields Reading Centre to a secure Google Cloud Healthcare DICOM store over an encrypted connection, where it will be analysed by the DeepMind algorithm. Results from this analysis will be logged in the eCRF database. The study's use of cloud computing infrastructure adheres to January 2018 guidance from NHS Digital regarding cloud computing for health and social care. All data will be handled in accordance with the Data Protection Act 2018.

6.2 Confidentiality of Trial Documents and Subject Records

The eCRF will not bear the subject's name or other personal identifiable data. A trial number will be used for identification on the CRFs. A separate log file which links the study ID and the patient's details, screening log and recruitment information will be kept on a protected NHS computer at hub sites. The key log will be kept at the recruitment site and will not be shared with the Sponsor. It will be the responsibility of the chief investigator or delegated trial member to ensure the accuracy of all data recorded on the CRFs. CRFs will be completed and signed off by the Chief Investigator or delegated/authorised individual as outlined in the delegation log, the completed CRFs will be checked for accuracy and completion by the trial co-ordinator prior to data entry.

6.3 Procedures for validation and securing of electronic clinical data systems

The eCRF will be developed by the Moorfields Eye Hospital database development team. The front end will use a bespoke web application and the back end (data storage) will be hosted on Moorfields Eye Hospital Research Database SQL servers. All servers are backed up daily and with multiple restore points every day and backup copies exist in more than one All MEH clinical trial databases are part of the MEH disaster recovery strategy and have a 5 day Recovery Time Objective.

6.4 Data handling and record keeping

Data queries will be sent to trial co-ordinators for clarification and confirmation whenever picked up. After all data queries are resolved and all errors are corrected, the database will then be locked with the agreement of King's CTU statistician and data will be exported by the applications manager and sent to trial statistician for data analysis. Pre-existing mechanisms for data transfer between Moorfields Eye Hospital CRF and King's CTU will be utilised.

Appendix 7. References

1 Kortuem K, Fasler K, Charnley A, et al. Implementation of medical retina virtual clinics in a tertiary eye care referral centre. *British Journal of Ophthalmology* 2018;102(10):1391-1395.

2 Agresti A and Coull B. Approximate is Better than “Exact” for Interval Estimation of Binomial Proportions. *The American Statistician* 1998;52(2):119-126.

3 Day A, Burr J, Bunce C, et al. Randomised, single-masked non-inferiority trial of femtosecond laser-assisted versus manual phacoemulsification cataract surgery for adults with visually significant cataract: the FACT trial protocol. *BMJ Open* 2015;5(11):p.e010381.

4 Theodossiades J, Murdoch I, and Cousens S. Glaucoma case finding: a cluster-randomised intervention trial. *Eye* 2004;18(5):483-490.

5 De Fauw J, Ledsam J, Romera-Paredes B, et al. Clinically applicable deep learning for diagnosis and referral in retinal disease. *Nature Medicine* 2018;24(9):1342-1350.

SPIRIT
STANDARD PROTOCOL ITEMS: RECOMMENDATIONS FOR INTERVENTIONAL TRIALS

SPIRIT 2013 Checklist: Recommended items to address in a clinical trial protocol and related documents*

Section/item	Item No	Description	Addressed on page number
**Administrative information**			
Title	1	Descriptive title identifying the study design, population, interventions, and, if applicable, trial acronym	1
Trial registration	2a	Trial identifier and registry name. If not yet registered, name of intended registry	2
	2b	All items from the World Health Organization Trial Registration Data Set	
Protocol version	3	Date and version identifier	2
Funding	4	Sources and types of financial, material, and other support	20
Roles and responsibilities	5a	Names, affiliations, and roles of protocol contributors	1
	5b	Name and contact information for the trial sponsor	
	5c	Role of study sponsor and funders, if any, in study design; collection, management, analysis, and interpretation of data; writing of the report; and the decision to submit the report for publication, including whether they will have ultimate authority over any of these activities	
	5d	Composition, roles, and responsibilities of the coordinating centre, steering committee, endpoint adjudication committee, data management team, and other individuals or groups overseeing the trial, if applicable (see Item 21a for data monitoring committee)	

For peer review only - http://bmjopen.bmj.com/site/about/guidelines.xhtml

**Introduction**			
Background and rationale	6a	Description of research question and justification for undertaking the trial, including summary of relevant studies (published and unpublished) examining benefits and harms for each intervention	4-6
	6b	Explanation for choice of comparators	4-6
Objectives	7	Specific objectives or hypotheses	6-8
Trial design	8	Description of trial design including type of trial (eg, parallel group, crossover, factorial, single group), allocation ratio, and framework (eg, superiority, equivalence, noninferiority, exploratory)	9
**Methods: Participants, interventions, and outcomes**			
Study setting	9	Description of study settings (eg, community clinic, academic hospital) and list of countries where data will be collected. Reference to where list of study sites can be obtained	9-10
Eligibility criteria	10	Inclusion and exclusion criteria for participants. If applicable, eligibility criteria for study centres and individuals who will perform the interventions (eg, surgeons, psychotherapists)	10
Interventions	11a	Interventions for each group with sufficient detail to allow replication, including how and when they will be administered	11-12
	11b	Criteria for discontinuing or modifying allocated interventions for a given trial participant (eg, drug dose change in response to harms, participant request, or improving/worsening disease)	12
	11c	Strategies to improve adherence to intervention protocols, and any procedures for monitoring adherence (eg, drug tablet return, laboratory tests)	
	11d	Relevant concomitant care and interventions that are permitted or prohibited during the trial	
Outcomes	12	Primary, secondary, and other outcomes, including the specific measurement variable (eg, systolic blood pressure), analysis metric (eg, change from baseline, final value, time to event), method of aggregation (eg, median, proportion), and time point for each outcome. Explanation of the clinical relevance of chosen efficacy and harm outcomes is strongly recommended	12-14
Participant timeline	13	Time schedule of enrolment, interventions (including any run-ins and washouts), assessments, and visits for participants. A schematic diagram is highly recommended (see Figure)	11-12, Figure 1, Figure 2

For peer review only - http://bmjopen.bmj.com/site/about/guidelines.xhtml

Sample size	14	Estimated number of participants needed to achieve study objectives and how it was determined, including clinical and statistical assumptions supporting any sample size calculations	Figure 1, 2, Appendix 4
Recruitment	15	Strategies for achieving adequate participant enrolment to reach target sample size	13, Appendix 1
**Methods: Assignment of interventions (for controlled trials)**			
Allocation:			
Sequence generation	16a	Method of generating the allocation sequence (eg, computer-generated random numbers), and list of any factors for stratification. To reduce predictability of a random sequence, details of any planned restriction (eg, blocking) should be provided in a separate document that is unavailable to those who enrol participants or assign interventions	11
Allocation concealment mechanism	16b	Mechanism of implementing the allocation sequence (eg, central telephone; sequentially numbered, opaque, sealed envelopes), describing any steps to conceal the sequence until interventions are assigned	
Implementation	16c	Who will generate the allocation sequence, who will enrol participants, and who will assign participants to interventions	11
Blinding (masking)	17a	Who will be blinded after assignment to interventions (eg, trial participants, care providers, outcome assessors, data analysts), and how	11
	17b	If blinded, circumstances under which unblinding is permissible, and procedure for revealing a participant’s allocated intervention during the trial	
**Methods: Data collection, management, and analysis**			
Data collection methods	18a	Plans for assessment and collection of outcome, baseline, and other trial data, including any related processes to promote data quality (eg, duplicate measurements, training of assessors) and a description of study instruments (eg, questionnaires, laboratory tests) along with their reliability and validity, if known. Reference to where data collection forms can be found, if not in the protocol	12, Appendix 1
	18b	Plans to promote participant retention and complete follow-up, including list of any outcome data to be collected for participants who discontinue or deviate from intervention protocols	

Data management	19	Plans for data entry, coding, security, and storage, including any related processes to promote data quality (eg, double data entry; range checks for data values). Reference to where details of data management procedures can be found, if not in the protocol	19, Appendix 6
Statistical methods	20a	Statistical methods for analysing primary and secondary outcomes. Reference to where other details of the statistical analysis plan can be found, if not in the protocol	15, Appendix 4
	20b	Methods for any additional analyses (eg, subgroup and adjusted analyses)	15-18
	20c	Definition of analysis population relating to protocol non-adherence (eg, as randomised analysis), and any statistical methods to handle missing data (eg, multiple imputation)	
**Methods: Monitoring**			
Data monitoring	21a	Composition of data monitoring committee (DMC); summary of its role and reporting structure; statement of whether it is independent from the sponsor and competing interests; and reference to where further details about its charter can be found, if not in the protocol. Alternatively, an explanation of why a DMC is not needed	19
	21b	Description of any interim analyses and stopping guidelines, including who will have access to these interim results and make the final decision to terminate the trial	
Harms	22	Plans for collecting, assessing, reporting, and managing solicited and spontaneously reported adverse events and other unintended effects of trial interventions or trial conduct	Appendix 2
Auditing	23	Frequency and procedures for auditing trial conduct, if any, and whether the process will be independent from investigators and the sponsor	
**Ethics and dissemination**			
Research ethics approval	24	Plans for seeking research ethics committee/institutional review board (REC/IRB) approval	19
Protocol amendments	25	Plans for communicating important protocol modifications (eg, changes to eligibility criteria, outcomes, analyses) to relevant parties (eg, investigators, REC/IRBs, trial participants, trial registries, journals, regulators)	

For peer review only - http://bmjopen.bmj.com/site/about/guidelines.xhtml

Consent or assent	26a	Who will obtain informed consent or assent from potential trial participants or authorised surrogates, and how (see Item 32)	Appendix 4
	26b	Additional consent provisions for collection and use of participant data and biological specimens in ancillary studies, if applicable	
Confidentiality	27	How personal information about potential and enrolled participants will be collected, shared, and maintained in order to protect confidentiality before, during, and after the trial	19
Declaration of interests	28	Financial and other competing interests for principal investigators for the overall trial and each study site	
Access to data	29	Statement of who will have access to the final trial dataset, and disclosure of contractual agreements that limit such access for investigators	19
Ancillary and post-trial care	30	Provisions, if any, for ancillary and post-trial care, and for compensation to those who suffer harm from trial participation	
Dissemination policy	31a	Plans for investigators and sponsor to communicate trial results to participants, healthcare professionals, the public, and other relevant groups (eg, via publication, reporting in results databases, or other data sharing arrangements), including any publication restrictions	19
	31b	Authorship eligibility guidelines and any intended use of professional writers	
	31c	Plans, if any, for granting public access to the full protocol, participant-level dataset, and statistical code	
**Appendices**			
Informed consent materials	32	Model consent form and other related documentation given to participants and authorised surrogates	Appendix 2
Biological specimens	33	Plans for collection, laboratory evaluation, and storage of biological specimens for genetic or molecular analysis in the current trial and for future use in ancillary studies, if applicable	Not applicable

*It is strongly recommended that this checklist be read in conjunction with the SPIRIT 2013 Explanation & Elaboration for important clarification on the items. Amendments to the protocol should be tracked and dated. The SPIRIT checklist is copyrighted by the SPIRIT Group under the Creative Commons "Attribution-NonCommercial-NoDerivs 3.0 Unported" license.

For peer review only - http://bmjopen.bmj.com/site/about/guidelines.xhtml
