# Supplementary material for: Teleophthalmology-enabled and artificial intelligence-ready referral pathway for community optometry referrals of retinal disease (HERMES): a Cluster Randomised Superiority Trial with a linked Diagnostic Accuracy Study—HERMES study report 1—study protocol
Source: BMJ Open. 2022 Feb 1;12(2):e055845. doi: 10.1136/bmjopen-2021-055845 (PMC8808461; doi:10.1136/bmjopen-2021-055845)
Supplement: Reviewer comments [file bmjopen-2021-055845.reviewer_comments.pdf]

PEER REVIEW HISTORY

BMJ Open publishes all reviews undertaken for accepted manuscripts. Reviewers are asked to complete a checklist review form (**http://bmjopen.bmj.com/site/about/resources/checklist.pdf**) and are provided with free text boxes to elaborate on their assessment. These free text comments are reproduced below.

ARTICLE DETAILS

**TITLE (PROVISIONAL)**	Tele-ophthalmology-enabled and Artificial Intelligence-ready referral pathway for community optometry referrals of retinal disease (HERMES): A Cluster Randomised Superiority Trial with a linked Diagnostic Accuracy Study HERMES Study Report 1: Study Protocol
**AUTHORS**	Han, Ji Eun; Liu, Xiaoxuan; Bunce, Catey; Douiri, Abdel; Vale, Luke; Blandford, Ann; Lawrenson, John; Hussain, Rima; Grimaldi, Gabriela; Learoyd, Annastazia; Kernohan, Ashleigh; Dinah, Christiana; Minos, Evangelos; Sim, Dawn; Aslam, Tariq; Patel, Praveen; Denniston, Alastair; Keane, Pearse; Balaskas, Konstantinos

This article was not externally reviewed at BMJ Open. Protocols that have been independently assessed prior to submission to BMJ Open are usually fast-tracked to publication on the grounds that further substantial changes will not be possible. This independent assessment will usually be external, independent review for both a substantial grant award from a non-commercial or government funder and ethics approval.
